# Epigenetics of Alzheimer’s Disease

**DOI:** 10.3390/biom11020195

**Published:** 2021-01-30

**Authors:** Matea Nikolac Perkovic, Alja Videtic Paska, Marcela Konjevod, Katarina Kouter, Dubravka Svob Strac, Gordana Nedic Erjavec, Nela Pivac

**Affiliations:** 1 Laboratory for Molecular Neuropsychiatry, Division of Molecular Medicine, Ruder Boskovic Institute, HR-10000 Zagreb, Croatia; mnikolac@irb.hr (M.N.P.); mkonjev@irb.hr (M.K.); dsvob@irb.hr (D.S.S.); gnedic@irb.hr (G.N.E.); 2 Medical Center for Molecular Biology, Institute of Biochemistry and Molecular Genetics, Faculty of Medicine, University of Ljubljana, SI-1000 Ljubljana, Slovenia; alja.videtic@mf.uni-lj.si (A.V.P.); katarina.kouter@mf.uni-lj.si (K.K.)

**Keywords:** epigenetics, mitoepigenetics, DNA methylation, DNA hydroxymethylation, miRNA, histone modifications, Alzheimer’s disease

## Abstract

There are currently no validated biomarkers which can be used to accurately diagnose Alzheimer’s disease (AD) or to distinguish it from other dementia-causing neuropathologies. Moreover, to date, only symptomatic treatments exist for this progressive neurodegenerative disorder. In the search for new, more reliable biomarkers and potential therapeutic options, epigenetic modifications have emerged as important players in the pathogenesis of AD. The aim of the article was to provide a brief overview of the current knowledge regarding the role of epigenetics (including mitoepigenetics) in AD, and the possibility of applying these advances for future AD therapy. Extensive research has suggested an important role of DNA methylation and hydroxymethylation, histone posttranslational modifications, and non-coding RNA regulation (with the emphasis on microRNAs) in the course and development of AD. Recent studies also indicated mitochondrial DNA (mtDNA) as an interesting biomarker of AD, since dysfunctions in the mitochondria and lower mtDNA copy number have been associated with AD pathophysiology. The current evidence suggests that epigenetic changes can be successfully detected, not only in the central nervous system, but also in the cerebrospinal fluid and on the periphery, contributing further to their potential as both biomarkers and therapeutic targets in AD.

## 1. Introduction

**Figure 1:**
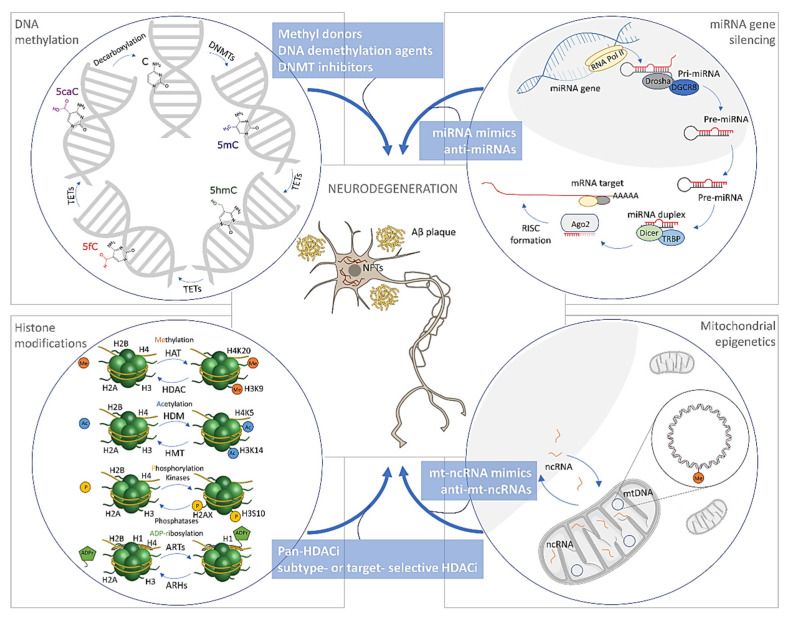
Epigenetic mechanisms in Alzheimer’s disease (AD) and possible treatment opportunities. 5caC—5-carboxyl cytosine; 5fC—5-formylcytosine; 5hmC—5-hydroxymethyl cytosine; 5mC—5-methylcytosine; Aβ—amyloid β; Ac—acetylation; Ago2—argonaute 2; ARHs—ADP-ribose hydrolases; ARTs—ADP-ribose transferases; C—cytosine; DGCR8—microprocessor complex subunit DGCR8; Dicer—endoribonuclease; DNMT—DNA methyltransferase; Drosha—ribonuclease III; H2, H3, H4—histones; HAT—histone acetyltransferase; HDAC—histone deacetylase; HDACi—HDAC inhibitors; K—lysine residues; Me—methylation; mt—mitochondrial; miRNA—microRNA; ncRNA—non-coding RNA; NFT—Neurofibrillary tangle; RISC—RNA-induced silencing complex; TET—ten-eleven translocation; TRBP—RISC-loading complex subunit TARBP2.

The field of epigenetics has significantly evolved since the early 1940s, when this concept was introduced by the British embryologist Conrad Waddington [1]. However, our understanding of the roles that epigenetic mechanisms play in various disease processes is still in its infancy. Epigenetic alterations have been implicated in the pathogenesis of different human diseases, including Alzheimer’s disease (AD). Regarding epigenetic alternations involved in AD pathogenesis, the research focus is directed towards DNA methylation and hydroxymethylation, histone posttranslational modifications, and non-coding RNA regulation (Figure 1). In addition, as in the case of nuclear DNA, mitochondrial gene expression is also regulated by epigenetic mechanisms, particularly DNA methylation and non-coding RNAs (Figure 1), resulting in a growing interest for exploring the association of mitochondrial epigenetics (mitoepigenetics) with AD.

This article gives a brief overview of recent findings regarding the role of epigenetics, including mitoepigenetics, in AD and the possibility of applying current knowledge in advancing AD therapy in the future. In our search, we focused only on original research articles exploring epigenetic alterations in AD, which included human samples and were published in English. Given that the aim of the paper was to present the epigenetic alterations associated with the AD, which is a human-specific disease, and present their biomarker potential, we decided to focus on studies that included human samples. We also considered all the studies that combined the research on human samples with animal and cell models. Articles that focused only on animal and/or cell models have not been taken into consideration; however, we took them into account when trying to elucidate the mechanism of action of individual epigenetic alterations. We also eliminated articles that did not include an adequate control group and that did not specify the number of included subjects per group.

## 2. Alzheimer’s Disease

AD is a predominant dementia-producing neurodegenerative disorder characterized by progressive memory loss and cognitive impairments with deficits in executive, language, and/or visuospatial functions, behavioral changes, and consequently death [2]. Since its initial description by Alois Alzheimer in 1906, understanding of the disease progression and clinical manifestations has improved, but the underlying etiology remains uncertain. AD is a slow and irreversible, but progressive, complex, and multifactorial neurodegenerative disorder, which represents the most common cause of dementia in older populations, and a major health problem worldwide [3]. The risk of developing AD significantly increases after 65 years of age and reaches up to 30% for individuals beyond age of 85 [4]. Since aging is the main risk factor for neurodegenerative diseases, the prevalence of AD increases with human longevity [5]. In 2015, there were around 46.8 million people with dementia worldwide, with AD contributing to 60–70% of cases [6], and this number is estimated to double every 20 years, reaching 74.7 million in 2030 and 131.5 million in 2050 [7]. According to the report from the Alzheimer’s Association in 2017, AD has affected around 6.08 million people in the United States [8], while AD prevalence in Europe was estimated at 5.05%, with 3.31% in men and 7.13% in women [9].

The etiology of AD is still not clear, however, neuroinflammation, extracellular plaques, and intracellular neurofibrillary tangles are the key pathological markers of the disease [10]. Extracellular plaques are made out of accumulated insoluble amyloid beta (Aβ) filaments which are derived from amyloid precursor protein (APP) by β-secretase 1 (BACE-1) and γ-secretase cleavage, while neurofibrillary tangles consist primarily of hyperphosphorylated protein tau. The main hallmarks of the disease are cortical and hippocampal neuronal losses, as well as the progressive degeneration of key neurotransmitter pathways [11].

Around 95% of hospitalized patients with AD have the sporadic form of the disease known as the late-onset Alzheimer’s disease (LOAD) [12]. The pathology of LOAD is multi-factorial and a result of biological, genetic, epigenetic, and environmental factors interacting with each other. An estimated heritability of LOAD is 60% to 80% [13,14], with apolipoprotein E (APOE) genotype as the strongest genetic risk factor. The APOE, a major lipid carrier in the central nervous system (CNS), possesses three variants, E2, E3, and E4, with APOE4 increasing the risk of developing LOAD. Aside from APOE, more than 20 LOAD risk genes involved in the lipid metabolism, innate immunity, and endocytosis have been identified by the genome wide association studies (GWAS) [14,15]. While LOAD manifests after the age of 65, AD that develops before the age of 65 is called the early onset AD (EOAD). Within the clinical picture of EOAD, a very rare autosomal dominant form of AD also exists [13]. This form of AD is associated with mutations in genes coding for APP, presenilin-1 (PSEN1), and PSEN2 [13]. However, those mutations explain only a small portion (5–10%) of cases, leaving the majority of autosomal-dominant pedigrees genetically unexplained [16].

Despite the overall research progress and modern diagnostic techniques, it is quite difficult to differentiate AD from the other dementia-causing neuropathologies. The diagnosis is based on the confirmation of memory loss and cognitive difficulties using neurological tests such as the Montreal Cognitive Assessment (MoCA) [17] and Mini-Mental Status Examination (MMSE) [18]. Some biomarkers can help to evaluate and mark the progress of AD pathology. Those include reduced Aβ levels in the cerebrospinal fluid (CSF) and detection of Aβ deposits or tau deposition in the brain by PET imaging [19,20,21]. The only definitive diagnosis of AD can be made post-mortem, with detection of the characteristic amyloid plaques and neurofibrillary tangles in the brains of affected patients. The biggest problem in AD is accurate diagnosis, estimated to be around 77%, as it relies on the clinical criteria and presence of the symptoms [22]. In the search for new, more reliable biomarkers, epigenetic modifications have emerged as important players in the pathogenesis of AD, with potential implications for the treatment of this currently incurable disease.

## 3. Epigenetic Alterations in Alzheimer’s Disease

### 3.1. DNA Methylation/Hydroxymethylation in Alzheimer’s Disease

Out of all known mechanisms of epigenetic regulation, the one being most studied and best understood is DNA methylation. In the process of DNA methylation, a single methyl group is added to the 5th atom of the cytosine ring [23]. The reaction is catalyzed by the DNA methyltransferase (DNMT) family of enzymes (Figure 1), and the donor of the methyl group is S-adenosyl methionine [24]. Additional to cytosine, adenine can also be methylated, but majority of DNA methylation occurs on cytosines, which are followed by guanine bases, forming stretches of DNA enriched in cytosine-phosphate-guanine motif, called CpG islands. There are approximately 28 million CpGs in the human genome, comprising 45,000 CpG islands [25]. CpG islands are usually not methylated and can serve as binding sites for transcription factors [26]. Because CpGs are often located in regulatory regions, DNA methylation can affect gene expression. While the addition of the methyl group does not disturb base pairing, it affects its biophysical properties. Due to the presence of the methyl group, the binding of transcriptional activators to DNA might be hampered [27]. Similarly, members of a protein family known as methyl-CpG binding domain proteins (MBDs) preferentially bind to methylated CpGs, serving as an anchor to which additional proteins can bind, leading to decreased gene expression [28]. DNA methylation plays a crucial role during embryogenesis. Later in life, it acts as an important mediator of environmental stimuli, helping cells adapt to various conditions [29]. DNA methylation is highly tissue-specific, facilitating various cells in our body to maintain their identity and adapt to stimuli. Some members of the DNA methylatrasferase family maintain a pattern of methylation during cell division (DNMT1), while others methylate previously unmethylated cytosines—de novo methylation of cytosines (DNMT3A and DNMT3B) [24]. DNA hydroxymethylation was first described in 1972 [30], but it was not until 2009 that it was identified as an oxidation metabolite of DNA methylation [31,32]. Active demethylation is regulated by enzymes of the ten-eleven translocation (TET) family, which catalyze 5-methylcytosine (5mC) conversion to 5-hydroxymethycytosine (5hmC) and two subsequent oxidative derivates, 5-formylcytosine and 5-carboxylcytosine (Figure 1). 5hmCs are often localized in gene bodies and the untranslated region [33]. Compared to methylation, levels of 5hmC are lower (roughly 10% of methylation levels), with higher percentage of 5hmC being observed in CNS [34]. Animal studies suggest its importance during neurodevelopment as levels of 5hmC in mice embryos increase in the absence of demethylation, making it a stable cell state [35]. 5hmC as such serves a dual role, not only as an intermediate of active demethylation, but as an important mark of epigenetic regulation as well. Similarly, as with DNA methylation, numerous environmental factors such as medications and pollutants can affect levels of 5hmC [36].

DNA methylation is crucial in maintaining basic cellular processes and synaptic plasticity in CNS, affecting cognitive functions [37]. Likewise, DNA hydroxymethylation represents an important factor during brain neurodevelopment and shows increased levels in CNS, suggesting the importance of its degeneration as well. The disturbance in both DNA methylation and DNA hydroxymethylation patterns has been associated with numerous disease states including neuropathologies [38]. For the purpose of this review, we focused on studies examining the effect of DNA methylation and DNA hydroxymethylation in AD patients (Table 1 and Table 2), while findings gathered using only animal or cell models were excluded. Our search in PubMed resulted in 29 articles that met all our inclusion and exclusion criteria (Table 1 and Table 2).

#### 3.1.1. DNA Methylation in Alzheimer’s Disease

Recent advances in sequencing technologies have allowed for a large pool of studies investigating DNA methylation in AD. DNA methylation of several specific genes has been investigated using candidate gene approach, with *APOE* being the most commonly studied gene. So far, inconsistent data have been reported, with DNA methylation being decreased in AD [39,40,41,42], while some studies found no differences in DNA methylation levels [43,44]. Additional genes of interest have been studied as well, including genes coding for brain-derived neurotrophic factor (*BDNF*) [45], glycogen synthase kinase 3 beta (*GSK3β)* [46], triggering receptor expressed on myeloid cells 2 (*TREM2*) [47], and ankyrin 1 (*ANK1*) [48]. Genomic studies demonstrated that no specific gene could be pinpointed as the sole carrier of AD pathology, but that a combination of various genetic variants and non-genetic factors increase the risk of AD. Since a similar situation applies for epigenetics, in our review we mainly inspected studies which analyzed DNA methylation on a genome-wide basis, enabling a broader view of the AD pathology (details in Table 1).

**Table 1:** Overview of studies investigating genome-wide DNA methylation in AD.

Epigenetic Mechanism	Effect	Gene/Target Pathway Involved	Study Model ^1^	Tissue/Study Design	Main Results	Ref.
5mC	↓	Neurogenesis, neurodevelopment, amyloid neuropathies	AD (*n* = 31), Moderate AD (*n* = 32), Ctrl (*n* = 38)	CNS (PFC)/NGS	Identification of 1224 DMRs (enhancer regions) in AD, including enhancers in the *DSCAML1* gene that targets *BACE1.*	[49]
5mC	↓↑	*WNT5B, ANK1, ARID5B*	AD (*n* = 96/104)	CNS (ECx)/methylation array technology	Experiment-wide significant increase of 5mC in *WNT5B* (single CpG). Increased levels of 5mC in *ANK1* (two probes). Decreased levels of 5mC in *ARID5B* (six probes).	[50]
5mC	↑	*ANKRD30B, ANK1* Cell adhesion, immunity	AD (*n* = 24), Neurotypical Ctrl (*n* = 49)	CNS (HPC, ECx, HPC, DLPFC, CB)/methylation array technology	Identification of 858 DMCs. Correlation between 5mC and gene expression levels.	[51]
5mC	↓	*B3GALT4, ZADH2* Cell survival, inflammation response	AD (*n* = 45), Ctrl (*n* = 39)	Blood/methylation array technology	Differential methylation of 477 DMCs, majority hypomethylated. Hypomethylation of *B3GALT4* and *ZADH2* associated with memory performance, and CSF levels of Aβ and tau.	[52]
5mC	↑	*HOXA3, GSTP1, CXXC1-3, BIN1*	AD (*n* = 18), Ctrl (*n* = 14)	CNS (FCx)/methylation array technology	Identification of 504 DMCs and 237 DMRs. Increased 5mC in pyramidal layer neurons in AD cases. 5mc pattern associated with oxidative stress.	[53]
5mC	↓	*KIAA0566*	NFT pathology stages I–VI (*n* = 17), Middle-aged cases (*n* = 3)	CNS (LC)/methylation array technology	Decreased levels of 5mC in *KIAA0566* in NFT pathology AD cases.	[54]
5mC	↑	*HOXA* gene cluster	Late-stage AD (*n* = 44), Middle stage AD (*n* = 43); Ctrl (*n* = 60)	CNS (PFC, STG)/methylation array technology	Identification of 208 DMCs in a 48kb *HOXA* gene cluster.	[55]
5mC	↓↑	*BRCA1*	AD cases (*n* = 30), Ctrl (*n* = 30)	CNS (ITG, CB, HPC, Ecx)/methylation array technology	Differential methylation of 8 DMRs in AD. Decreased levels of 5mC in *BRCA1* in AD. *BRCA1* 5mC correlated with *APOE* e4 allele status.	[56]
5mC	↑	Neuregulin receptor complex signaling pathway	AD cases (*n* = 10), Ctrl (*n* = 10)	CNS (TCx)/methylation array technology	Differential methylation of 161CpG positions associated with miRNA genes.	[57]
5mC	↑	Neuron function and development, cholesterol/lipid metabolism	AD cases (*n* = 34), Ctrl (*n* = 34)	CNS (STG)/methylation array technology	Identification of 479 DMRs, majority hypermethylated. Overlap of hypermethylated DMRs and histone trimethylation marks.	[58]
5mC	↑	*ANK1*	Cohorts (*n* = 117/144/62)	CNS (Ecx, STG, PFC)/methylation array technology	Increased levels of 5mC in *ANK1*, associated with Braak stage. Strong correlation of top 100 DMCs between the cohorts.	[59]
5mC	↓↑	*ANK1, BIN1, RHBDF2*	Cohorts (*n* = 708/117)	CNS (DLPFC)/methylation array technology	Identification of 71 DMC associated AD pathology burden. Validation of 11 DMRs in an independent set.	[60]
5mC	↑	Molecular functions associated with transcription, membrane transport, and protein metabolism	AD (*n* = 12), Ctrl (*n* = 12)	CNS (FCx)/methylation array technology	Identification of 948 DMCs in AD.	[61]
5mC	↓	*AS3MT, TBX15, WT1*	AD with psychosis (*n* = 29), AD without psychosis (*n* = 18)	CNS (PFC, Ecx, STG)/methylation array technology and pyrosequencing	Decreased levels of *ASM3T* 5mC (previously associated with SZ). Decreased levels of *TBX1* and *WT1* 5mC (both previously associated with AD).	[62]
5mC	↓	5mC cell subtype localization	Early-AD (*n* = 5), Late-AD (*n* = 5), Ctrl (*n* = 5)	CNS (ITG)/immunohistochemistry	Decreased localization of extranuclear 5mC marks in neurofilament-positive pyramidal neurons and decreased localization of nuclear 5mC marks in astrocytes in AD cases compared to controls.	[63]
5mC	↑	None stated	EOAD and LOAD (*n* = 29), Ctrl (*n* = 29)	CNS (MFG, MTG)/immunohistochemistry	Increased levels of 5mC in MFG and MTG of AD patients. Positive correlation of 5mC with 5hmC and AD markers (Aβ, tau, and ubiquitin loads).	[64]
5mC	↑	None stated	AD (*n* = 7), Preclinical AD (*n* = 5), Ctrl (*n* = 5)	CNS (HPC/PHG, CB) mimunohistochemistry	Increased levels of 5mC in HPG of both AD patients and preclinical AD cases compared to control group subjects.	[65]
5mC	↓	None stated	AD (*n* = 10), Ctrl (*n* = 10)	CNS (HPC)/immunohistochemistry	Decreased levels of 5mC in AD Negative correlation between 5mC and amyloid plaque load.	[66]

↓—decreased levels; ↑—increased levels; Aβ—amyloid beta; AD—Alzheimer’s disease; *ANK1*—ankyrin 1 gene; *ANKRD30B*—ankyrin repeat domain 30B gene; *APOE*—apolipoprotein E gene; *ARID5B*—AT-rich interaction domain 5B gene; *AS3MT*—arsenite methyltransferase gene; *B3GALT4*—beta-1,3-galactosyltransferase 4 gene; *BACE1*—beta-secretase 1 gene; *BIN1*—bridging integrator 1 gene; *BRCA1*—BRCA1 DNA repair associated gene; CB—cerebellum; CNS—central nervous system; Ctrl—control subjects; *CXXC1-3*—CXXC finger protein 1 gene; DLPFC—dorsolateral prefrontal cortex; DMCs—differentially methylated ctyosines; DMRs—differentially methylated regions; *DSCAML1*—DS cell adhesion molecule like 1 gene; ECx—nteorhinal cortex; EOAD—early onset Alzheimer’s disease; FCx—frontal cortex; *GSTP1*—glutathione S-transferase Pi 1 gene; *HOXA3*—homeobox A3 gene; HPC—hippocampus; HPG—hippocampus/parahippocampal gyrus; ITG—inferior temporal gyrus; *KIAA0566*—ATPase phospholipid transporting 10A gene; LC—locus coeruleus; LOAD—late onset Alzheimer’s disease; MFG—middle frontal gyrus; MTG—middle temporal gyrus; NGS—next-generation sequencing; PFC—refrontal cortex; *RHBDF2*—rhomboid 5 homolog 2 gene; STG—superior temporal gyrus; *TBX15*—T-Box transcription factor 15 gene; TCx—temporal cortex; *WNT5B*—Wnt family member 5B gene; *WT1*—WT1 transcription factor gene; *ZADH2*—zinc binding alcohol dehydrogenase domain containing 2 gene. ^1^ Number of subjects per group is presented as discovery cohort/validation cohort.

A major advantage of the next-generation sequencing (NGS) approach is the ability to determine the exact genomic locations where 5mC patterns were altered. Regardless, no clear conclusion can be drawn as studies reported both increased and decreased levels of DNA methylation, which could be partially due to the use of various tissue samples. Significant increase in DNA methylation has been reported so far in various brain regions including the hippocampus, entorhinal cortex, and dorsolateral prefrontal cortex [51], the temporal cortex [57], and the temporal gyrus [58]. A DNA methylation decrease was observed in the prefrontal cortex and locus coeruleus [49,54]. Similarly, a decrease in DNA methylation was observed in blood samples as well [52]. Even though no common gene was identified in all examined studies, the methylation of *ANK1* was increased in AD patients in four studies [50,51,59,60]. ANK1 is an integral membrane protein, important in cell proliferation, activation, and mobility, by mediating the attachment of membrane proteins (receptors, ion channels, cell adhesion proteins) [67].

The following two studies are an insightful example of epigenetic intertwinement of DNA methylation and other epigenetic modifications. Data analysis of Villela et al. focused on non-coding RNA genes [57]. About 13% of analyzed noncoding RNA CpG sites were differentially methylated in AD, with a majority showing increased levels of 5mC. Altogether, the authors identified 161 microRNAs (miRNAs), of which 10 miRNAs have already been previously associated with AD. Selected miRNAs have been involved in neuron myelination process and targeted some of the genes involved in the AD pathology, such as *APP*, *BACE1*, and sirtuin 1 (*SIRT1)*. The miRNAs not only regulate the expression of their target genes, but they themselves can be regulated by DNA methylation of genes encoding them. Similarly, Watson et al. observed additional brain-specific histone signatures [68]. Poised promoter regions were identified as promoter regions, containing both activating H3K27me3 and repressing H3K27me3 histone marks. When comparing data with known histone modification marks, overlap was observed between differentially methylated DNA regions in AD and histone marks H3K27me3 and H3K4me3 in poised promoters [68]. A recent study by Pishva et al. compared genome-wide methylation pattern between AD cases with and without psychosis [62]. While there was no significant difference in global DNA methylation between studied groups, specific loci of multiple genes, previously associated with schizophrenia and AD, did exhibit similar 5mC pattern across all the brain regions [62]. This is particularly important since approximately 40% of AD patients experience additional symptoms of psychosis, associated with a rapid decline in disease progression.

Although immunohistochemistry lacks the base resolution of NGS, this approach enables an easier tissue localization or cell type differentiation, which is important when discussing epigenetic regulation (details for immunohistochemistry AD studies are presented in Table 1). While some studies found no difference in 5mC mark localization [69], others revealed contrasting results—by observing either a decrease or an increase in 5mC localization. Decreased levels of 5mC mark were detected in astrocytes and pyramidal neurons [63], hippocampal CA1 neurons, and glia [66], whereas increased levels were observed in the middle frontal gyrus and middle temporal gyrus [64], as well as the hippocampus/parahippocampal gyrus [65].

#### 3.1.2. DNA Hydroxymethylation in Alzheimer’s Disease

With 5hmC being a rather recent discovery, not many studies have been published. Our PubMed search identified 11 published papers examining DNA hydroxymethylation associated with AD in human subjects (details in Table 2). Ten studies examined genome-wide levels of 5hmC using NGS or immunohistochemistry methodology.

**Table 2:** Overview of studies investigating DNA hydroxymethylation in AD.

Epigenetic Mechanism	Effect	Gene/Target Pathway Involved	Study Model ^1^	Tissue/Study Design	Main Results	Ref.
5hmC	↓	*FBXL16, ANK1*	AD (*n* = 96/104)	CNS (ECx)/methylation array technology and pyrosequencing	Decreased levels of 5hmC in *FBXL16* (four probes). Decreased levels of 5hmC in *ANK1* (4 CpGs).	[50]
5hmC	↑↓	*BIN1* Signaling, energy metabolism, cell function processes, gene expression, protein degradation, and cell structure and stabilization	LOAD (*n* =3), Ctrl (*n* = 2)	CNS (HPC)/RRHP	Identification of 15.158 (DhMR), majority hyperhydroxymetylated.	[70]
5hmC	↑↓	*ABAT, CAMK1D, HTRA3, LRRN1* Long term memory and neurotrophin signaling pathway	AD (*n* = 20), MCI (*n* = 4), Ctrl (*n* = 6)	CNS (DLPFC)/NGS	Identification of 517 DhMRs, associated with neuritic plaques, and of 60 DhMRs, associated with neurofibrillary tangles. Correlation between 5hmC and gene expression.	[71]
5hmC	↑↓	Neuron projection development, neurogenesis	AD (*n* = 3/2), Ctrl (*n* = 3/2)	CNS (PFC)/NGS	Identification of 7601 DhMR in the discovery set. Identification of 2351 DhMR in the replication set.	[72]
5hmC	↓↑	5hmC cell subtype localization	EOAD (*n* = 5), LOAD (*n* = 5), Ctrl (*n* = 5)	CNS (ITG)/immunohistochemistry	Decreased localization of nuclear 5hmC marks in AD cases compared to controls. No differences in localization of 5hmC in neurofilament-positive pyramidal neurons, disease-resistant calretinin-interneurons, microglia in AD cases compared to control subjects.	[63]
5hmC	None	None	AD (*n* = 12; 10 sporadic + 2 familial), Ctrl (*n* = 14)	CNS (ECx, CB)/immunohistochemistry	No significant difference in 5hmC levels between studied groups.	[69]
5hmC	↓	None stated	AD (*n* = 13), Ctrl (*n* = 8)	CNS (ECx, CB)/immunohistochemistry	Decreased levels of 5hmC in both ECx and CB of AD patients compared to control group subjects.	[68]
5hmC	↑	5hmC cell subtype localization	EOAD and LOAD (*n* = 29), Ctrl (*n* = 29)	CNS (MFG, MTG)/immunohistochemistry	Increased levels of 5hmC in MFG and MTG of AD patients. Positive correlation of 5hmC with 5mC and AD markers (Aβ, tau, and ubiquitin loads). Differences in cell subtype 5hmC distribution (lower levels in astrocytes and microglia, higher levels in neurons).	[64]
5hmC	↑	None stated	preclinical AD (*n* = 5), AD (*n* = 7), Ctrl (*n* = 5)	CNS (HPG, CB)/immunohistochemistry	Increased levels of 5hmC in HPG of both AD patients and preclinical AD cases compared to control group subjects.	[65]
5hmC	↓	5hmC cell subtype localization	AD (*n* = 10), Ctrl (*n* = 10) monozygotic twins (AD twin and non-AD affected twin)	CNS (HPC)/immunohistochemistry	Decreased levels of 5hmC in AD. Decreased levels of 5hmC in CA3 HPC region glial cells and overall decrease in neuronal cells in AD. Negative correlation between 5hmC and amyloid plaque load. Decreased levels of in 5hmC of the AD twin compared to the non-AD affected twin.	[66]
5hmC	None	*TREM2*	AD (*n* = 12), Ctrl (*n* = 5)	CNS (HPC) 5hmC DNA immunoprecipitation/RT-qPCR	No significant difference in *TREM2* 5hmC levels between studied groups.	[73]

↓—decreased levels; ↑—increased levels; 5hmC—5-hydroxymethylated cytosine; AD—Alzheimer’s disease; CB—cerebellum; CNS—central nervous system; Ctrl—control subjects; DhMRs—differentially hydroxymethylated regions; DLPFC—dorsolateral prefrontal cortex; ECx—entorhinal cortex; EOAD—early onset Alzheimer’s disease; HPC—hippocampus; HPG—hippocampus/parahippocampal gyrus; ITG—inferior temporal gyrus; LOAD—late onset Alzheimer’s disease; MCI—mild cognitive impairment; MFG—middle frontal gyrus; MTG—middle temporal gyrus; NGS—next-generation sequencing; RRHP—reduced representation 5-hydroxymethylcytosine profiling; RT-qPCR—reverse transcription quantitative real-time PCR; *TREM2*—triggering receptor expressed on myeloid cell gene. ^1^ Number of subjects per group is presented as discovery cohort/validation cohort.

Using NGS approach, Bernstein et al. identified thousands of differentially hydroxymethylated regions (DhMRs) in a discovery and replication set of AD cases and control subjects [72]. The majority of them had an increase in 5hmC levels located in intragenic regions [72]. Another pilot study by Ellison et al. identified over 15,000 DhMR, with over half of them showing an increase in 5hmC in AD cases, predominately in promotor and gene bodies [70]. Pathway analysis identified AD associated genes, related to signaling, energy metabolism, cell function processes, gene expression, protein degradation, and cell structure and stabilization [70]. While results of both pilot studies are promising, further confirmational studies are necessary as the small sample size lacks the needed statistical power. A larger sample was analyzed in a study by Zhao et al., which included mild cognitive impairment (MCI) subjects as well [71]. Regarding an association with neuritic plaques and neurofibrillary tangles, both traits showed predominately increased levels of 5hmC in DhMRs, with four genes overlapping [71]. A study by Smith et al. found no experiment-wide significant results for Braak-associated 5hmC in entorhinal cortex [48], which is believed to be the starting point of AD pathology in cortex. Additional analysis of spatially correlated genomic regions (at least two DhMRs) identified increased levels of 5hmC in four probes of the F-box and leucine rich repeat protein 16 (*FBXL16*) gene [50]. *FBXL16* has been previously associated with AD, exhibiting decreased expression in microglia of mouse AD model [74]. Using pyrosequencing on an additional cohort of subjects, decreased levels of 5hmC in four CpGs were also confirmed in *ANK1* [50].

Multiple studies used the immunohistochemistry approach as it allows a better cell type differentiation. Decreased levels of 5hmC were observed in AD entorhinal cortex and cerebellum [68], and in glial cells of CA3 hippocampal region [66], while there was an increase in the middle frontal gyrus, middle temporal gyrus [64], and parahippocampal gyrus of AD subjects [65]. In addition to an increase in 5hmC levels, Bradley-Whitman and Lovell observed a decrease in levels of 5fC and 5caC of both AD patients and preclinical AD cases compared to control subjects [65]. Different cell types/formations in inferior temporal gyrus showed increased (neurofibrillary tangles) and decreased (astrocytes) localization [63], while one study found no significance in 5hmC localization in entorhinal cortex and cerebellum of AD patients [69]. Chouliaras et al. additionally examined CA1 hippocampal region in a pair of monozygotic twins and observed a decreased localization of 5hmC in glial cells of AD-affected twin compared to the twin without AD [66].

A single study examined 5hmC of a targeted candidate gene. Celarain et al. focused on triggering receptor TREM2, a transmembrane glycoprotein receptor whose genetic variants could increase the risk of AD [73]. *TREM2* is expressed mainly in microglia cells and is involved in homeostasis, tissue repair, and innate immune response. Immunoprecipitation of hippocampal tissue targeting promotor region, exon 2, and 3′-UTR revealed no significant difference in 5hmC between AD patients and control subjects. However, there was a positive correlation of *TREM2* 5hmC enrichment in exon 2 with *TREM2* expression level. The authors proposed that the observed increase in gene expression could present a way of brain tissue repair [73].

#### 3.1.3. Mitochondrial DNA Methylation in Alzheimer’s Disease

Mitochondria represent the energy source of the cell, and AD has been proposed to be linked to bioenergetics decline stemming from the dysfunction of the mitochondria [75,76,77,78]. Impairments in the mitochondrial energy-generating pathway—oxidative phosphorylation, increased reactive oxygen species production, and apoptosis are some of the important processes in the pathophysiology of neurodegeneration [78].

Mitochondrial DNA (mtDNA) is circular, double-stranded molecule of 16,569 bp. It contains 2 ribosomal RNA (rRNA) genes, 22 transfer RNA (tRNA) genes, and 13 genes for mitochondrial proteins of the respiratory chain [79]. The rest of the proteins necessary for mitochondrial maintenance are encoded by the nuclear DNA and are transported to mitochondria via protein translocases residing in the outer and inner mitochondrial membranes [80]. The mitochondrial genome is intronless, while the transcription and replication control non-coding region is termed as D-loop [81]. Mitochondrial DNA is devoid of nucleosomes, and instead is packed as nucleoides—discrete protein-DNA complexes composed of multiple mtDNA molecules and several different proteins [82].

In mtDNA, several extensive deletions have been reported to be associated with AD [83], as well as mutations of cytochrome c oxidase [84], tRNAs and rRNAs [85,86], and mutations in the regulatory D-loop, which affect transcription, translation, and mtDNA copy number [87].

Very interesting initial findings on mtDNA content in CSF have been published by Podlesniy et al. [88]. Patients exhibiting AD biomarkers of low Aβ and high t-tau in CSF and presymptomatic patients with *PSEN1* mutation, all showed lower content of mtDNA in CSF compared to controls. The authors speculated that low mtDNA copy number as well as defective biogenesis of mitochondria could be reflected in the low content of the mtDNA in CSF, which could serve as a biomarker already in the preclinical stage of the AD [88]. In a subsequent study, Podlesniy et al. further confirmed previous results where early markers for AD, Aβ and p-tau, showed positive and negative correlation with CSF levels of mtDNA, respectively [89]. At the same time, markers for neuronal damage, t-tau, did not show any correlation. When using the ratio of CSF mtDNA and p-tau concentrations, the achieved sensitivity and specificity for diagnosis of slow progression AD were 93% and 94%, respectively. Based on these results, the low mtDNA in CSF, jointly with low Aβ and high p-tau, provides a great potential for differential diagnosis of AD against other neurological disorders [89]. Although the two studies proposed the mtDNA in CSF as new potential biomarker for early AD detection, the study by Cervera-Carles et al. failed to replicate these results [90]. Although Cervera-Carles et al. used the same approach, tested the same amplicon of 85 base pairs (designated as mtDNA-85), and showed that the mtDNA in CSF is indeed a robust biomarker, they found great interindividual variability of mtDNA concentrations in AD patients and control subjects, and failed to replicate the association with AD [90].

Mitochondrial epigenetics, often termed “mitoepigenetics”, is a rather new field of research and so far, limited number of studies of mtDNA methylation exist. Representing less than 1% of the cellular DNA, mtDNA harbors 435 CpG sites and 4747 non-CpG cytosine residues [91]. Due to different methodological approaches, ranging from radiolabeling and restriction enzymes [92,93,94], enzyme-linked immunosorbent assays [95], to PCR-based techniques coupled with bisulfite conversion [96], and immunoprecipitation coupled with NGS [97,98], the results on mtDNA methylation have been rather conflicting. Since mtDNA accounts for only a small portion of the total cellular DNA, and the methylation levels of mtDNA are as low as only few percent, methods for methylation detection have to be sensitive. Moreover, the other important issue is the selection of the interrogated sequence of mtDNA, since the CpG rich region is not ubiquitously present throughout the mtDNA. With the finding of Shock et al. in 2011, who proved translocation of DNMT1 to mitochondria and hence contributed to the discovery of the part of the methylation mechanism, existence of mtDNA methylation (and indirectly hydroxymethylation) became unambiguous [99]. In addition, DNMT3a, DNMT3b, TET1, and TET2 have been detected in the mitochondria [100,101].

Blanch et al. found increased mtDNA methylation at CpG and non-CpG sites of D-loop in the entorhinal cortex of AD patients with Braak stages I to II and III to IV, when compared to control subjects [102]. However, differences in the 5hmC levels were not detected [102]. Stoccoro et al. analyzed D-loop methylation levels in blood samples of AD patients and control subjects, and observed significant reduction of mtDNA methylation in AD patients (25%) [103]. In the same group of subjects, polymorphisms of the genes involved in one-carbon metabolism (*MTHFR*, *MTRR*, *MTR*, and *RFC-1*) and DNA methylation reactions (*DNMT1*, *DNMT3A*, and *DNMT3B*) were analyzed in association with mtDNA methylation. The results demonstrated that *MTRR* 66>G and *DNMT3a* -448A>G polymorphisms were significantly associated with levels of D-loop methylation [104].

### 3.2. Histone Modifications in Alzheimer’s Disease

Histone proteins (H1, H2A, H2B, H3, and H4) associate with DNA to form nucleosomes as fundamental units of chromatin, and they represent an essential part of eukaryotic transcription regulation [105,106]. Specifically, histone modifications are involved in the repression or activation of gene expression as they influence nucleosome stability, chromatin-mediated processes, and histone-histone interactions [105,107]. Such posttranslational modifications (Figure 1), including methylation, acetylation, phosphorylation, ubiquitination, sumoylation, glycosylation, biotinylation, and ADP-ribosylation, occur on specific residues of the histone N-terminal “tail” domain [105,108,109]. Out of all histone modifications, acetylation has been the most studied. Histone acetylation at lysine residues, catalyzed by histone acetyltransferases (HATs), has been associated with transcriptional activation and “open” chromatin conformation, making DNA more accessible for the transcriptional machinery, whereas histone deacetylation, regulated by histone deacetylases (HDACs), has been involved in transcriptional repression and “closed” chromatin structure [106]. Histone acetylation, as a rather transient covalent modification, plays an important role in the regulation of DNA replication, transcription, and various other cellular functions [105]. Histone methylation is one of the most complex post-translational modifications [105,110]. Methyl groups are added or removed from lysine, arginine or histidine residues by different enzymes, such as methyl-transferases and demethylases (Figure 1). The transcriptional effect of histone methylation is dictated in a site-specific manner and by the number of methylation marks at a specific residue, resulting in numerous functional outcomes underlying phenotypic diversity [106,110]. Therefore, it is not surprising that histone methylation takes part in numerous cell processes, including mitosis, meiosis, DNA repair, transcription, differentiation, response to stress, and aging [105]. Histone phosphorylation at serine, threonine, or tyrosine residues is regulated by the activities of protein kinases and protein phosphatases and its function differs among different histones and various sites [106]. Histone phosphorylation is also involved in a variety of cellular processes, such as mitosis, gene transcription, and chromatin condensation [105]. Ubiquitination is an enzymatic process that starts with the activation of ubiquitin by ubiquitin-activating enzyme, followed by conjugation to a cysteine residue by ubiquitin-conjugating enzyme and its transfer to a substrate via lysine residue by ubiquitin-protein isopeptide ligase. Ubiquitination of different histones has different and often opposite functions, such as transcription activation or silencing. The role of ubiquitination, especially H2A and H2B histones, has been identified in DNA repair, silencing, initiation, and elongation of transcription [111]. In addition to covalent histone modifications, histones can be reversibly modified by ADP-ribosylation, which is regulated by specific enzymes. Mono- and poly-ADP ribosylated histones play an important role in chromatin structure regulation, DNA repair, cell cycle, replication, and transcription [112,113]. Finally, crosstalk of histones is also important and different histone modifications work together to regulate gene transcription in different directions [106,114].

Histone modifications play an important role not only in neuronal development, but also in aging brain, as well as in AD pathogenesis [115,116]. Widespread loss of heterochromatin has been observed in tau transgenic Drosophila and mice and in human AD, and is suggested to promote tau-mediated neurodegeneration and aberrant gene expression in AD [117]. Oxidative stress and subsequent DNA damage have been identified as a mechanistic link between transgenic tau expression and heterochromatin relaxation [117]. Here we focused on studies examining histone modifications associated with AD in human subjects, and we excluded the findings obtained only on animal or cell models of AD (Table 3). Our search in PubMed resulted in 14 articles that met all our inclusion and exclusion criteria (Table 3).

Acetylation dysregulation has been associated with various impairments in signaling, proliferation, inflammation, immunity, apoptosis, and neuronal plasticity [129]. So far, two epigenome-wide association studies (EWAS) investigated histone acetylation in postmortem AD brain samples using chromatin immunoprecipitation sequencing (ChIP-seq) [130]. EWAS conducted by Marzi et al. investigated H3K27 histone acetylation in postmortem AD brain samples and identified 4162 differentially acetylated peaks, of which many were located in genes implicated in AD pathology or AD genetic risk variants (APP, PSEN1, PSEN2, and MAPT) [118]. AD-associated differentially acetylated peaks in the entorhinal cortex were enriched in processes related to processes related to pathology (e.g., lipoprotein binding, Aβ metabolic process) and neuronal activity (e.g., GABA receptor activity, synaptic proteins) [118]. Klein et al. profiled H3K9 histone acetilation in the dorsolateral prefrontal cortex and identified that in contrast with amyloid-β, tau protein burden had a broad effect on the epigenome, affecting 5990 of 26,384 H3K9ac domains [131]. The study also suggested that the 17-DMAG or alvespimycin could be used as a potential therapeutic for altering the widespread chromatin remodeling associated with tau pathology [131]. Decreased histone acetylation has been found in temporal lobe of subjects with AD [123]. Specifically, lower levels of acetylated lysine 16 on histone H4 (H4K16ac), which is involved in DNA damage and aging, were observed in cortex of AD patients in comparison to healthy aged subjects [116,119]. On the other hand, increased acetylation of lysine 12 on histone H4 has been associated with memory impairment [116], with higher H4K12ac levels found in MCI, but not in AD patients, supporting its involvement in early stage of aggregate formation and disease development [109]. In addition, increased levels of acetylated and total H3 and H4 histones have been observed in human post-mortem AD brain [119]. In animal models of AD, decreased acetylation of lysine 27 on histone H3 (H3K27) has been detected in regions associated with plasticity, whereas increased acetylation has been found in regions responsible for immunity [129]. Moreover, in AD patients, higher levels of histone deacetylases (HDACs), especially class I HDACs (HDAC2 and HDAC3), the enzymes which catalyze removal of acetyl groups and repress the transcription by condensing chromatin, have been observed in certain brain regions responsible for learning, memory, and neuroplasticity and are associated with impairments in cognitive and synaptic functions [116,132]. However, other studies reported reduction of HDAC levels in brain regions affected by disease, which was correlated with MCI symptoms. Class II HDACs are also involved in pathogenesis of AD. Increased levels of HDAC6 were observed in the hippocampus and cortex of AD patients, as well as in animal models of AD [133]. The HDAC6 affects tubulin acetylation, as well as tau phosphorylation and degradation and is involved in inflammatory processes [134,135]. Reduction of HDAC6 levels leads to higher clearance and reduction of tau aggregation and might help neuronal survival [129,136], whereas overexpression of HDAC6 decreases α-tubulin acetylation, and consequently impairs microtubules stability, vesicular, and mitochondrial transport. Some authors showed that reduction of HDAC4, another class II enzyme, has adverse effects on memory formation and learning. HDAC4 may play an important role in nerve function, since overexpression of HDAC4 leads to apoptosis and its inactivation suppresses neuronal cell death [137]. Class III HDACs, so-called sirtuins, are important in memory and synaptic plasticity and play an important role in the pathogenesis of AD [135]. SIRT1 levels have been decreased in the parietal cortex, but not in the cerebellum of AD patients [129]. Such alterations have been associated with Aβ and tau accumulation [138], as well as tau acetylation of lysine 28, which leads to extensive tau aggregation [129,132].

In addition to acetylation, alternations in histone methylation have been observed in AD. Balance between histone methyltransferases and histone demethylases is important for brain integrity and memory in AD [112]. Increased trimethylation of lysine on histone H3 (H3K9), a marker of gene silencing and condensation of heterochromatin structure [139], as well as higher levels of histone methyltransferase EHMT1 mRNA were found in the postmortem brain of subjects with AD [140]. HMT G9a, the enzyme specific for H3K9 di-methylation, is linked to cognitive performance in mice, whereas H3K4 demethylase is associated with memory deficits in humans [112].

Moreover, increased phosphorylation of serine on histone H3 (H3S10) [127], as well as increased phosphorylation of H2AX at Ser139, as evidence of DNA damage [126], have been observed in AD hippocampal neurons and astrocytes, respectively. In addition, ADP-ribosylation of histone H1 has been found in different AD brain regions [128]. Moreover, increased levels of 4-hydroxynonenal, metabolite of lipid peroxidation, might interact with histones and alter DNA-histone interaction, leading to higher oxidative damage [134]. All these findings support an important role of histone modifications occur in AD; however, the pattern of changes is very complex and further research is needed.

### 3.3. The microRNAs in Alzheimer’s Disease

Non-coding RNAs (ncRNAs) are not translated into proteins but they are crucial in regulating numerous cellular functions by binding DNA, RNA, and proteins, and thus influencing gene expression, mRNA translation, and assembly of protein complexes [141,142]. We can distinguish two types of ncRNAs, housekeeping and regulatory ncRNAs. Housekeeping ncRNAs include transfer RNAs (tRNAs), ribosomal RNAs (rRNAs), small nuclear RNAs (snRNA), and small nucleolar RNAs (snoRNAs). Regulatory ncRNAs are important in modulating gene expression and they can be subdivided according to their length into short chain ncRNAs (small interfering RNAs, microRNAs (miRNAs), piwi-interacting RNAs) and long non-coding RNAs (lncRNAs). In this review, we focus on miRNAs and their potential in improving early diagnosis of AD, as well as their perspective for the treatment of AD. The miRNAs are 19 to 24 nucleotides long single-stranded RNAs with an important role in post-transcriptional gene silencing. They destabilize targeted mRNAs by imperfectly binding to the corresponding sequence, which is usually found in the 3′-untranslated regions of targeted mRNAs. The miRNAs emerge from hairpin-structured precursors, so called primary miRNAs (pri-miRNAs). The hairpin-structured transcripts are processed by two RNase III-type enzymes, Drosha and Dicer [143,144], and converted into precursor miRNAs (pre-miRNAs) and, finally, into small miRNAs (Figure 1). One strand from the miRNA duplex is removed and the other one, the miRNA strand [145], becomes a part of RNA-induced silencing complex (RISC) and serves for targeted recognition of specific mRNAs (Figure 1) [146]. In animals, most of miRNAs are only partially complementary to their targeted mRNAs. This complementarity region is most commonly reduced to 6 to 7 nucleotides located in miRNA 5′ proximal “seed” region [146]. The final result of miRNA action is translational repression and/or degradation of targeted mRNA [147]. Today we know of more than 2000 different miRNAs that regulate gene expression in humans [148], with each miRNA regulating the expression of maybe hundreds of different genes, and interacting with various histone modifications and DNA methylation.

Research regarding miRNA dysregulation in AD began in the 1990s and early 2000s and since then miRNAs have been broadly investigated due to their potential as biomarkers of AD pathogenesis. Circulating miRNAs can be detected in the peripheral circulation (serum, plasma, exosomes, whole blood, peripheral blood mononuclear cells) as well as in the CSF. The miRNAs are quite stable in different biological fluids, compared to mRNA [149]. Non-circulating miRNAs are linked to brain tissue (hippocampus, cerebellum, temporal, frontal, and parietal cortex). The most interesting are miRNAs that can be detected in both the CNS and the periphery, due to their significant biomarker potential. The studies investigating the role of various miRNAs in AD pathology differed in terms of selection of comparable groups (comparing AD with MCI group and cognitively normal control and/or other disease control subjects), sample size, approach (candidate approach and/or high throughout technologies), and/or methodology (RT-qPCR and/or NGS, microarray, NanoString) [150]. In our review, we focused only on original research articles exploring miRNA expression in AD, which included human samples. Our search in PubMed resulted in 83 articles that met all our inclusion and exclusion criteria (Table S1). Due to the extremely large number of articles taken into account, we focused on miRNAs that were most interesting (Table 4), however, the data regarding other potentially important miRNAs are available as supplementary material (Table S1).

Many of the investigated miRNAs target genes are directly involved in the pathophysiology of AD. They are implicated in APP degradation and Aβ metabolism by regulating the activity of enzymes, which are involved in APP cleavage, like BACE1 [209]. Different miRNAs have been found to modulate the expression of BACE1, including miR-15b, miR-29c, miR-124, miR-135b, miR-195, and miR-339-5p [210,211,212,213]. Some miRNAs, like miR-219, target microtubule associated protein tau (MAPT) gene [214] or they regulate the activity of different protein kinases responsible for the phosphorylation of tau protein, such as miR-124-3p and miR-125b [167,189,215]. Synaptic plasticity is also regulated by miRNAs. Specifically, BDNF, a key regulator of synaptic plasticity and transmission, has been suggested to induce the expression of miR-132 [216]. The expression of the miR-132/212 family members has been downregulated in early AD [194,217] and it was suggested that miR-132 targets the gene coding for methyl CpG binding protein 2 (MeCP2), responsible for increasing BDNF levels in the brain tissue. Kawashima et al. demonstrated that the BDNF-dependent increase in the expression of postsynaptic proteins could be decreased by inhibiting miR-132 function [217].

Until now, there have been 61 different miRNAs extensively studied and associated with the development of AD (Table S1). Out of 61 miRNAs considered (Table S1), 48 had altered expression in blood samples and 19 in CSF samples of AD patients when compared to an adequate control. However, the results regarding specific miRNAs were often contradictory or not repeated (Table 4, Table S1). The direction of change for specific miRNAs is listed in Table 4 and Table S1. Many different miRNAs have also been found deregulated in the brain tissue of AD patients (*n* = 17), but the results are inconsistent for the most miRNAs analyzed [218]. The details regarding some of the most studied non-circulating miRNAs are presented in Table 4. From the data presented in Table 4 and Table S1, it is clear that certain miRNAs are more often associated with the development of AD and detected as circulating and non-circulating miRNAs. These are miRNAs that could potentially serve as biomarkers of AD (Table 4) and should be closely investigated in future studies. Therefore, hereinafter we briefly present the most interesting candidates.

#### 3.3.1. miR-9

miR-9 is involved in the regulation of proliferation, migration, and differentiation of neural progenitor cells [219]. The findings regarding the dysregulation of miR-9 in AD are contradictory (Table 4). In the case of downregulation, the role of miR-9 in the AD pathogenesis could be explained by its negative effect on BACE1 regulation [220], and consequently increased Aβ production and aggregation [221]. Downregulation of miR-9 was also associated with targeting calcium/calmodulin-dependent protein kinase kinase 2 (CAMKK2) transcripts [222], leading to increased levels of p-tau and amyloidogenesis through CAMKK2-cyclic adenosine monophosphate-activated protein kinase (AMPK) pathway [223]. Upregulation of miR-9 in AD could be associated with its influence on transforming growth factor, β-induced (TGFBI), tripartite motif-containing 2 (TRIM2), and SIRT1 expression [221]. Discrepancies in the results (Table 4) could be due to the recruitment of patients at different stages of AD progression.

#### 3.3.2. miR-29

There are many studies investigating the role of miR-29 in human subjects and most of these studies have detected downregulation of miR-29a/b/c in blood samples, CSF samples, and different brain regions of patients diagnosed with AD (Table 4). The miR-29 potentially regulates BACE1 expression [224,225], suggesting that BACE1 overexpression in AD could be due to decreased levels of miR-29. It was suggested that miR-29a targets the transcript for neuron navigator 3 (NAV3), whose mRNA is elevated in the frontal cortex of AD patients [226]. However, the exact role of NAV3 in AD is yet unknown. The miR-29 was found to suppress the expression of five members of B-cell lymphoma 2 (Bcl-2) Homology 3 (BH3)-only protein family [227]. These proteins play a role in apoptosis by triggering the release of cytochrome c from the inner mitochondrial membrane [228]. Downregulation of the miR-29 in AD suggests that this miRNA could be the cause of increased apoptosis rate seen in individuals diagnosed with AD.

#### 3.3.3. miR-34

Most of the studies exploring the role of miR-34 in AD reported upregulation of this miRNA in different brain regions and blood mononuclear cells of AD patients (Table 4). Bcl-2 emerged as a possible target of miR-34a [229]. This antiapoptotic protein inhibits the action of caspase-9 and improves neuron survival [230]. Therefore, the increased expression of miR-34a could inhibit the expression of BCL2 and make neurons more exposed to apoptosis. In addition, in mice brain, protein p53 was associated with miR-34a, and p53/miR-34a axis was shown to promote cell apoptosis by suppressing SIRT1 and BCL2 gene expression and by activating caspase-3 [231,232]. The miR-34a was also suggested to target tau mRNA [233], however, the effect is not clear since both miR-34a and tau are upregulated in AD.

#### 3.3.4. miR-107

miR-107 was downregulated in blood samples and CNS of AD subjects, especially during the early stages of the disease (Table 4). Wang et al. demonstrated negative correlation between miR-107 expression and levels of BACE1 protein, suggesting BACE1 mRNA as a target of miR-107 [182]. These predictions were confirmed by Nelson and Wang [234]. Except for BACE1, miR-107 has other targets connected to pathogenesis of AD, including neurotrophic factor granulin (GRN), involved in neurite outgrowth, and cofilin, which is involved in actin-filament disassembly [235,236,237]. Cyclin dependent kinase 5 regulatory subunit 1 (CDK5R1) gene is also regulated by miR-107 [238]. The protein encoded by CDK5R1 (p35) is a neuron-specific activator of cyclin-dependent kinase 5 (Cdk5), an enzyme which is required for normal development and function of CNS [239]. Other identified targets of miR-107 are disintegrin and metalloproteinase domain-containing protein 10 (ADAM10) gene encoding α-secretase [240], an enzyme responsible for cleaving transmembrane region of APP.

#### 3.3.5. miR-125

miR-125b was downregulated in plasma and serum samples of AD patients compared to control subjects (Table 4). However, in CNS, miR-125b expression was mostly upregulated when compared to controls (Table 4). Elevated miR-125b levels were shown to induce tau hyperphosphorylation and lead to elevated p35 expression and mitogen-activated protein kinase/extracellular signal-regulated kinases (MAPK/ERK) signaling [167]. Considering that Cdk5/p35, along with Erk1/2, phosphorylates tau, the miR-125b mediated upregulation of kinase expression and activity can be linked to tau pathology in AD. Study by Banzhaf-Strathmann identified two additional targets of miR-125b, tau phosphatase protein phosphatase 1 catalytic subunit alpha (PPP1CA) and the anti-apoptotic protein B-cell lymphoma-w (Bcl-W). PPP1CA dephosphorylates tau, and the levels of this protein were found to be significantly reduced in frontal cortex of AD patients [167]. The involvement of Bcl-W in tau phosphorylation is still not clarified.

#### 3.3.6. miR-132/-212

The miR-132/-212 gene locus was associated with cognitive capacity [241] and has been persistently downregulated in different brain areas (Table 4). In mice, the miR-132/212 deficiency was associated with increased tau phosphorylation, expression, and pathological aggregation [195]. It was suggested by Weinberg et al. that miR-132/122-mediated upregulation of the SIRT1 pathway in MCI could act as a compensatory mechanism at the beginning of cognitive dysfunction [196].

#### 3.3.7. miR-146

There is a large amount of data in the literature regarding the association of miR-146 with the development of AD; however, the results of the studies are contradictory demonstrating both downregulation and upregulation of this miRNA in serum, plasma, CSF, and CNS of AD subjects (Table 4). The evidence suggests that transcription of miR-146a is regulated by nuclear factor kappa-B (NF-κB) [204]. By promoting miR-146 transcription, NF-κB suppresses translation of complement factor H (CFH) and affects inflammatory response in the CNS [204]. Dysregulation of this system in AD leads to increased inflammation and neurodegeneration. Cell treatment with IL-1β and Aβ led to upregulation of NF-κB and miR-146a, confirming the relationship between NF-κB and miR-146 [242]. Lukiw also reported downregulation of both CFH and tetraspanin 12 (TSPAN12) mRNA by miR-146a [242], while Li et al. showed decreased mRNA expression of CFH and interleukin-1 receptor-associated kinase 1 (IRAK-1) as a consequence of miR-146a upregulation [243]. Downregulation of these proteins facilitates neuroinflammation and amyloidogenesis.

#### 3.3.8. mR-155

miR-155 was upregulated in peripheral blood mononuclear cells (PBMCs) and different brain regions of patients diagnosed with AD (Table 4). This miRNA is one of the most studied miRNAs related to immune response. Using in vitro model (SH-SY5Y cells with APP695 mutation), it was demonstrated that higher production of APP and Aβ is associated with upregulation of inflammatory-associated miRNAs, miR-155, miR-146a, and miR-124 [244]. The role of miR-155 in inflammation was also confirmed in mice treated with lipopolysaccharide [245]. Study by Li et al. reported elevated expression of inflammation-related miRNAs, including the miR-21, miR-125a, miR-146a, and miR-155 in serum-derived exosomes [245]. Additionally, miR-155 was associated with AD through its influence of T lymphocyte function [246].

#### 3.3.9. miR-181

miR-181 was mostly downregulated in AD CNS and serum samples (Table 4). In animal models of AD, the downregulation of miR-181c was linked to increased expression of Aβ [247], and it was suggested that miR-181c downregulations mostly affect the MAPK signaling pathway. Geekiyanage et al. detected miR-181c binding site in the 3′-UTR region of serine palmitoyltransferase long chain base subunit 1 (SPTLC1) gene [153]. The authors showed positive correlation between SPTLC1 and Aβ expression in AD brain, and suggested that the downregulation of miR-181c can increase the abundance of pathogenic Aβ by dysregulating the expression of SPTLC1 and increasing the levels of ceramide [153]. TRIM2, SIRT1, and BTB Domain Containing 3 (BTBD3) were also suggested as additional targets of miR-181 [248], along with high mobility group protein 1 (HMGB1), Bcl-2, and nicotinamide phosphoribosyltransferase (NAMPT) [249].

#### 3.3.10. miR-206

miR-206 has been elevated in AD plasma, serum, and CSF samples (Table 4). This miRNA was shown to be a modulator of BDNF expression in a way that upregulation of miR-206 expression downregulates the expression of BDNF [250,251]. The role of miR-206 in AD was also confirmed by administration of donepezil in APP/PS1 mice, which reversed the elevated expression of miR-206 in the mice hippocampus and cortex [252].

## 4. Treatment Opportunities through Epigenetics

The treatment of AD has not significantly changed or improved in the last decade. It includes acetylcholine esterase inhibitors donepezil, galantamine, and rivastigmine, and NMDA receptor antagonist memantine. Although many new drugs with novel mechanism of action were effective in animal models (nicotinic receptor agonists, glutamate receptor modulators, gamma secretase inhibitors, grow factors, statins, monoclonal antibodies, tau inhibitors, serotonin receptor modulators), most of them had serious side effects, whereas only a few showed the efficacy in improving cognitive decline in humans. Consequently, they were suspended in phases 1–3 of clinical trials, resulting in no new AD medications in the last ten years. Therefore, there is an urgent need to develop new medications with a disease-modifying effect to reduce the progress of AD. The effect of environment and drugs that could change epigenetic landscape of the cells in human body is one of the most interesting fields in a contemporary research.

The evidence presented in this review emphasizes the involvement of DNA methylation in AD progression and pathology, thus pointing out the importance of exploring potential epigenetic therapeutics that would modulate aberrant DNA methylation pattern in AD at a very early stage of the disease. The question remains whether the observed dysregulation of 5mC and 5hmC levels is a part of the cause or simply the consequence of AD. In a subset analysis, examining only cognitively non-impaired subjects compared to all study subjects, De Jager et al. noticed changes in 5mC similar to those in AD patients [60]. Similarly, an increased expression of ten-eleven translocation methylcytosine dioxygenase 1 (*TET1*), as well as increased levels of 5mC and of 5hmC were observed in parahippocampal gyrus in both AD patients and preclinical AD subjects compared to control group [65]. The findings of these two studies suggest that the molecular changes appear before the onset of clinical symptoms. The DNA methylation could therefore present a diagnostic biomarker; however, it should be extensively validated. As these changes have only been observed in brain, tissue availability also presents a major concern.

Most of the epigenetic drugs targeting histone modifications (Figure 1), as potential therapeutics for AD, belong to the group of HDAC inhibitors (HDACi) [108]. HDACi have been shown to reduce AD hallmarks such as tau phosphorylation and Aβ production, and improve memory formation, learning, and spatial memory, as well as increase synaptic plasticity [253]. The pan-HDAC inhibitors include vorinostat (SAHA), trichostatin A (TSA), valproic acid (VPA), sodium butyrate, sodium 4-phenylbutyrate (4-PBA), and suberoylanilide hydroxamic acid, which interact with zinc-dependent HDAC proteins and affect class I, II, and IV HDACs, while nicotinamide, as the precursor of NAD+, is a specific inhibitor for class III HDACs [253]. Valproic acid inhibits production of amyloid beta peptide in vitro and in vivo, and reduces mRNA level of NF-κB in a mice model of AD. On the other hand, phenylbutyrate and sirtuin inhibitor nicotinamide reduced phosphorylated tau and ameliorated cognitive function, leading to memory and learning restoration [135,137]. Another potential therapeutic approach might be increase in HATs [254]. Several HATs, including CBP (cAMP-response element binding protein), p300 and PACAF (p300/CBP-associated factor) showed more specific performance than non-selective HDACi. However, HATs are not reliable in AD treatment due to low membrane permeability and solubility [254]. The treatment with two recently developed HDACi, hydroxamide-based class I and II HDACi and mercaptoacetamide-based class II HDACi with better penetration through blood brain barrier and longer half-life, resulted in restoration of learning and memory in AD mice [255]. However, HDACi are usually non-selective and affect not only histones in the nucleus, but also other proteins in cytoplasm [129]. Since it was shown that increased HDAC2 and HDAC3 activity has a negative impact on cognition while reduced HDAC1 activity may be neurotoxic, HDAC-based therapy that would inhibit HDAC2 or HDAC3, but not HDAC1 would represent a great step towards AD treatment [140]. On the other hand, there are HDACi, such as tubacin and suramin, which demonstrate great selectivity for HDAC6, Sirt1, and Sirt2 [137]. HDAC6 inhibitors, such as M344, were shown to additionally increase histone acetylation, decrease microglia inflammation, and exert neuroprotective effects [136]. Other HDACi, such as MS-275, W2, and RGFP-966, have shown some promise in studies of AD, since they appear to reduce AD pathology in vitro and memory impairments in vivo [140]. In addition to HDACs and HATs, inhibitor of acetyltransferases (INHAT) can also regulate histone acetylation, through histone-masking, in which INHAT binds to histones and masks their access to HAT [256]. As a key component of INHAT, level of ANP32A, the inhibitor of protein phosphatase-2A, is selectively upregulated in the brain of AD patients [257,258]. Downregulating ANP32A rescues synaptic plasticity and memory ability in tau transgenic mice model by reducing INHAT formation and unmasking histone for hyperacetylation [259]. Histone methyltransferase inhibitors might also have potential therapeutic effects in AD. However, large loss of methyltransferase function has been associated with learning deficiencies in both AD patients and mouse models of AD. One of the solutions for this problem might be a partial histone methyltransferase inhibition that would help restore balanced enzyme function [140]. In summary, in search for a new therapeutic approach to AD, a complex balance between both histone acetylation and deacetylation, as well as other histone modifications should be taken into consideration [112,129].

The miRNAs have become extremely interesting therapeutic targets because of their ability to regulate the endogenous gene expression with the possibility of only one miRNA regulating entire biological pathways. Therefore, miRNA-based therapy has a great therapeutical potential in diseases in which the cause is complex and related to a number of genes and biological processes. The idea is to select a specific miRNA that targets multiple mRNAs, which are altered in certain pathological conditions, and target these miRNAs with specific anti-miRNAs or use them to design miRNA mimics (Figure 1). However, by targeting a particular miRNA, we are affecting a large number of transcripts, potentially resulting in certain side effects. This is also a problem with a large number of currently available and prescribed therapeutics. The idea behind miRNA mimics, which are artificial miRNA duplexes very similar to specific miRNA precursors, is to downregulate the expressions of targeted genes/proteins involved in disease pathogenesis [260]. Another approach would be the use of anti-miRNA therapies in order to completely or partially eliminate the function of the miRNA of interest [261]. All the miRNAs discussed in this review represent potential targets for future studies that will try to implement anti-miRNAs or miRNA mimics as personalized treatment approaches in individuals diagnosed with AD [261]. However, another current challenge is the delivery of the therapeutic agent to the desired location in the human body. In addition to viral vectors, liposomes and nanoparticles, the use of exosomes for these purposes has been increasingly considered [262], since the transport of miRNAs is one of their key roles in long-distance intercellular communication. Even though miRNA research in vitro and in animal models of AD suggest the potential of certain miRNAs in AD therapy, this is still a relatively new direction in AD research.

In addition, we have to point out a potential of directly targeting specific genetic alterations as a strategy for AD treatment. Contemporary methods of gene editing include the use of programmable DNA binding proteins such as such zinc finger proteins (ZFP), transcription activator-like effectors (TALE), and RNA-guided clustered regularly interspaced short palindromic repeats (CRISPR)/CRISPR-associated protein 9 (Cas9) [263]. The latest genome editing technology, CRISPR/Cas9, showed several advantages over ZFP and TALE, and demonstrated promising potential in the treatment of several neurological disorders, including AD [264,265,266]. The CRISPR/Cas9 system, in which Cas9 endonuclease is able to target specific DNA sequences with the help of guide RNAs (gRNAs) [267], can be used to target and correct any specific gene sequences, including AD genetic risk factors. The mutant form of the Cas9, called dead Cas9 (dCas9), advanced the CRISPR/Cas9 gene editing tool and resulted in CRISPR interference (CRISPRi) technology, when dCas9 is fused or interacts with transcriptional repressors, and in CRISPR activation (CRISPRa) technology, when dCas9 is fused or interacts with transcriptional activators [268]. In the light of treatment opportunities through epigenetics in AD, very promising results were gained with targeting histone demethylase [269], histone acetyltransferase [270], and histone methyltransferases [271,272], with dCas9 fusion proteins. Several studies reported successful methylation of CpG island induced by dCas9 fused to the catalytic domain of specific DNMTs [273,274,275,276,277], while others induced targeted hydroxylation of methylated CpGs with the help of dCas9 fused with the catalytic domain of TET1 [274,278,279,280]. CRISPR/Cas9 technology can also be used to target specific ncRNAs by incorporating ncRNA into the gRNA sequence [281]. However, there are many challenges that still need to be overcome in order to target CRISPR/Cas9 to specific cell types, as in the case of miRNAs, and future studies will need to elucidate the real therapeutic potential of CRISPR/Cas9 in AD.

Interesting evidence regarding the effects on mitochondrial epigenome are coming from neuropsychiatric studies, suggesting that epigenetic changes are dynamic and reversible [282]. However, although there is a developing potential for mitochondrial epigenome to become an applicable biomarker, there is still a long way to go before mitochondria specific drugs will be available.

## 5. Conclusions

Different epigenetic mechanisms play crucial roles in the development and pathogenesis of AD, and there is a great potential for using them as disease biomarkers or as a new strategic approach to AD treatment. Investigation of the CSF represents a great potential in the AD diagnostics, since it enables monitoring of CNS metabolism, due to its closeness to the brain parenchyma [283]. Combination of CSF biomarkers, currently used for AD diagnosis, include increased levels of t-tau and p-tau and decreased levels of Aβ. Achieved sensitivity and specificity of these biomarkers in AD diagnosis is about 90%, and additionally, these biomarkers can be used for monitoring disease progression and are at the same time indicators for early stages of AD [284]. However, we have to keep in mind that all candidate biomarkers have to pass rigorous screening processes, and that several critical issues (analytical validity, clinical validity, and clinical utility) must be addressed, in order to reach routine clinical application. One of the major problems that studies exploring potential epigenetic biomarkers of AD have to face is the variability of sociodemographic and clinical attributes in researched subjects, leading to discrepancies between the results. In order to obtain applicable data, great attention should be paid to all possible co-variants and the selection of appropriate healthy controls (age-matched, sex-matched, with similar lifestyle and education). Secondly, it is very important to have reliable clinical diagnosis, which is often very hard to achieve when determining the correct cause of dementia. Standardization and simplification of sample collection, preparation protocols, and procedures are also crucial in order to be able to draw some objective conclusions. Finally, methods and technologies used to analyze epigenetic alterations are continuously being improved, but their cost-effectiveness is still questionable. Despite all the problems highlighted, we expect that over time more reliable epigenetic markers will be identified and incorporated into multi-target assays. Given the growing threat of rapidly increasing incidence of AD worldwide, it is essential to direct the focus to developing inexpensive, rapid, and simple methods that will allow several epigenetic biomarkers to be measured at the same in order for them to be adopted into clinical practice.

Results imply the involvement of 5mC and 5hmC in AD pathology and progression (Table 1 and Table 2). However, studies mentioned above are hard to compare directly due to different brain tissue regions analyzed (including bulk tissue or specific cell types), various methodologies applied and differences in subjects (e.g., Braak stage) enrolled. Additionally, DNA methylation is a process that is being directly affected by various external factors during the course of our lives while also exhibiting tissue specificity [285]. Combined with the use of different methodologies, each carrying specific advantages and biases, study results need to be interpreted with caution [286]. When choosing the best method for DNA methylation analysis, a broader perspective should be taken into consideration, including factors such as the type and amount of biological sample, quality of the starting DNA sample, effect size desired to observe, and costs of the analysis. With technology rapidly evolving, multiple methods for analyzing DNA methylation are available; from base pair specific (most popular being methylation array technology, next generation sequencing, and pyrosequencing) to a more qualitative method (such as immunohistochemistry) [286]. In the end, the choice of the method best suitable for DNA methylation analysis often remains dependent on the question asked by the researcher.

Although a large body of evidence from animal models and cell culture experiments has supported an important role of histone modifications in AD, the results of studies in human AD brain tissue are scarce and further research is needed (Table 3). Specifically, the pattern of histone changes appears very complex, and a balance between various histone modifications should be taken into consideration. HDACi have shown most promising potential for AD therapy. However, many issues still need to be resolved before these compounds can be effectively used in AD. Nevertheless, it is becoming evident that subtype- or target-selective HDACi may be more applicable as novel therapeutic strategies for AD, rather than relatively broad-spectrum HDACi.

Various miRNAs are included in the pathology of AD and have a significant biomarker potential since they can be non-invasively monitored in body fluids. Specifically, miRNAs are stable in body fluids and, for many of them, the results of different studies have shown the same trend in terms of deregulation in AD patients (Table 4; Table S1). The miRNAs that have been detected as differently expressed in individuals with AD compared to control subjects are not only involved in amyloidogenic and inflammatory pathways, but also in the regulation of other biological systems. Better understanding of the role of specific miRNAs in AD can broaden, not only our knowledge of the disease, but also the pool for the development of novel AD therapies. Zhang et al. preformed a meta-analysis including 10 different studies and showed that miRNAs have a great diagnostic potential with the overall sensitivity of 0.80 (95% CI: 0.75–0.83), specificity of 0.83 (95% CI: 0.78–0.87), and diagnostic odds ratio of 14 (95% CI: 11–19) [287].

In a few studies, mtDNA in CSF was presented as an interesting biomarker [88,89,288], since dysfunctions in the mitochondria, including the impaired mitochondrial biogenesis [289], as well as lower mtDNA copy number [76], were shown to be a part of the AD pathophysiology. Low levels of mtDNA in CSF can already be detected in the preclinical stage of the AD, and could help differentiate between AD and other neurodegenerative disease, including frontotemporal degeneration [88] and Creutzfeldt-Jakob disease [288].

Reviewing the literature supports the conclusion that the interest of exploring the epigenetics of AD is constantly growing and the amount of data available regarding this topic is extensive and difficult to synthesize. The current evidence suggest that epigenetic changes can be successfully detected, not only in the CNS, but also in the CSF and on the periphery, contributing further to their potential as both biomarkers and therapeutic targets in AD.

## Figures and Tables

**Table 3:** Overview of studies investigating histone modifications in AD.

Epigenetic Mechanism	Effect	Gene/Target Pathway Involved	Study Model	Tissue/Study Design	Main Results	Ref.
Acetylation (H3K27)	↑	*CR1, GPR22, KMO, PIM3, PSEN1, RGCC*	AD (*n* = 24), Ctrl (*n* = 23)	CNS (Ecx)/ChIP-seq	Acetylated peaks identified close to the genes involved in tau and amyloid neuropathologies.	[118]
Acetylation (H3, H4, acetyl histone H3, acetyl histone H4)	↑	Genes implicated in AD development and synaptic plasticity. *BACE1, PSEN1*	AD (*n* = 14), Ctrl (*n* = 17)	CNS (ITG, MTG)/immunohistochemistry and tissue microarrays	Significant positive correlations found between ubiquitin load and histone modifications.	[119]
Acetylation (H4K12, H4K16)	↑	None stated	AD (*n* = 6), Ctrl (*n* = 6)	CNS (FCx)/LC-MS/MS	First study to report changes in methylation of H2BK108 and H4R55, and ubiquitination of H2BK120 in FCx of AD subjects.	[120]
			AD (*n* = 34), MCI (*n* = 15), Ctrl (*n* = 31)	Monocytes/quantification fluorometric kit	Significantly elevated acetylation of H4K12 in human patients with MCI but not in patients with AD.	[121]
Acetylation (HDAC1, HDAC2)	↓	None stated	AD (*n* = 8), Ctrl (*n* = 7)	CNS (FCx, HPC)/Western blot	HDAC1 and HDAC2 are decreased in FCX, while HDAC1 is decreased in HPC of AD patients.	[122]
Acetylation (H3K9K14, H2B)	↑	None stated	AD (*n* = 8), Ctrl (*n* = 7)	CNS (FCx, HPC)/Western blot	Increased histone levels associated with the cytoplasmic fraction and pointing to a dysregulation in histone catabolism in FCx.	[122]
Acetylation (H3K18/K23)	↓	None stated	AD (*n* = 11), Ctrl (*n* = 4)	CNS (TCx)/LC-MS/MS and Western blot	Histone acetylation significantly lower in AD temporal lobe than in aged controls.	[123]
Acetylation (HDACs)	↑	None stated	AD (*n* = 25), Ctrl (*n* = 25)	Plasma/colorimetric HDAC activity assay	Plasma levels of HDACs might be used as peripheral biomarkers of AD.	[124]
Methylation (H2BK108, H4R55)	↓	None stated	AD (*n* = 6), Ctrl (*n* = 6)	CNS (FCx)/LC-MS/MS	First study to report changes in methylation of H2BK108, methylation of H4R55, and ubiquitination of H2BK120 in FCx of AD subjects	[120]
Phosphorylation (H3)	↑	None stated	AD (*n* = 10), BD (*n* = 10), Ctrl (*n* = 10)	CNS (FCx)/ELISA	Increased H3 phosphorylation in AD and BD subjects indicate an onset of apoptosis and cell death.	[125]
Phosphorylation (H2AX)	↑	None stated	AD (*n* = 13), Ctrl (*n* = 13)	CNS (HPC, TCx)/immunohistochemistry	Increased DNA damage in the astrocytes of AD brains, as evidenced by the astrocytic nuclear accumulation of γH2AX.	[126]
Phosphorylation (H3)	↑	None stated	AD (*n* = 17), Ctrl (*n* = 9)	CNS (HPC)/immunohistochemistry	Study implicates that neurons in AD are mitotically activated. This is in line with other studies that indicate that the cell cycle is activated in AD neurons.	[127]
Ubiquitination (H2BK120)	↑	None stated	AD (*n* = 6), Ctrl (*n* = 6)	CNS (FCx)/LC-MS/MS	First to report changes in methylation of H2BK108, methylation of H4R55, and ubiquitination of H2BK120 in AD FCx.	[120]
Poly(ADP-ribosyl)ation	↑	Gnenes coding for nuclear proteins: *MAP2, GFAP, CD68*	AD (*n* = 20), Ctrl (*n* = 10)	CNS (FCx, TCx)/immunohistochemistry	Findings indicate that there is enhanced PARP activity in AD.	[128]

↓—decreased levels; ↑—increased levels; AD—Alzheimer’s disease; *BACE1*—beta-Secretase 1 gene; CB—cerebellum; *CD68*—CD68 antigen gene; ChIP-seq—ChIP-sequencing; CNS—central nervous system; *CR1*—complement C3b/C4b receptor 1 gene; Ctrl—control subjects; ECx—entorhinal cortex; ELISA—enzyme-linked immunosorbent assay; FCx—frontal cortex; *GFAP*—glial fibrillary acidic protein gene; *GPR22*—G protein-coupled receptor 22 gene; H—histone; HDAC—histone deacetylase; HPC—hippocampus; ITG—inferior temporal gyrus; K—lysine; *KMO*—kynurenine 3-monooxygenase gene; LC-MS/MS—liquid chromatography with tandem mass spectrometry; *MAP2*—microtubule associated protein 2 gene; MTG—middle temporal gyrus; *PIM3*—serine/threonine kinase Pim-3 gene; *PSEN1*—presenilin 1 gene; R—arginine; *RGCC*—regulator of cell cycle gene; TCx—temporal cortex.

**Table 4:** Short overview of studies investigating miRNA expression in AD. More detailed overview is given in Supplementary Materials (Table S1).

Epigenetic Mechanism	Effect	Study Model ^1^	Tissue Study/Design	Ref.
miR-9-5p	↓	AD (*n* = 69), PD (*n* = 67), Ctrl (*n* = 78)	CSF/miRNA-seq	[151]
		Braak III-VI (*n* = 20), Braak 0-I (*n* = 7)	CNS (HPC, MFG, CB)/RT-qPCR	[152]
		AD (*n* = 7), Ctrl (*n* = 7)	CNS (neocortex)/RT-qPCR	[153]
		AD (*n* = 27), Ctrl (*n* = 18)	CNS (TCx)/miRNA-seq/RT-qPCR	[154]
	↑	AD (*n* = 10/18), Ctrl (*n* = 10/18)	CSF/RT-qPRC	[155]
		AD (*n* = 6), Ctrl (*n* = 6)	CNS (TCx)/array and RT-qPCR	[156]
		AD (*n* = 6), Ctrl (*n* = 6)	CNS (TCx)/array	[157]
		AD (*n* = 5), Ctrl (*n* = 5)	CNS (TCx)/RT-qPCR	[158]
		AD (*n* = 5), Ctrl (*n* = 5)	CNS (HPC)/array	[159]
miR-29a(-3p)	↓	AD (*n* = 10), Ctrl (*n* = 10)	CSF/RT-qPCR	[160]
		AD (*n* = 50/16); Ctrl (*n* = 49/16)	CSF/array and RT-qPCR	[161]
		AD (*n* = 7), Ctrl (*n* = 7)	CNS (neocortex)/RT-qPCR	[153]
	↑	AD (*n* = 18), Ctrl (*n* = 20)	CSF/RT-qPCR	[162]
		Braak III-VI (*n* = 20), Braak 0-I (*n* = 7)	CNS (HPC, MFG, CB)/RT-qPCR	[152]
miR-29b(-3p)	↓	Probable AD (*n* = 7), aMCI/Probable Early AD (*n* = 7), Ctrl (*n* = 7)	Serum/RT-qPCR	[163]
		AD (*n* = 35), Ctrl (*n* = 35)	Plasma (exosomes)/miRNA-seq	[164]
		AD (*n* = 48), Ctrl (*n* = 22)	Blood/omiRas and DIANA miRPath	[165]
		AD (*n* = 28), Ctrl (*n* = 25)	PBMC/RT-qPCR	[166]
		AD (*n* = 10), Ctrl (*n* = 5)	CNS (FCx)/RT-qPCR	[167]
		AD (*n* = 5), Ctrl (*n* = 5)	CNS (PCx) array	[168]
	↑	Braak III-VI (*n* = 20), Braak 0-I (*n* = 7)	CNS (HPC, MFG, CB)/RT-qPCR	[152]
miR-29c(-3p)	↓	AD (*n* = 30), Ctrl (*n* = 30)	Blood/RT-qPCR	[169]
		AD (*n* = 20), Ctrl (*n* = 20)	Serum/miRNA-seq & RT-qPCR	[170]
		AD (*n* = 28), PD (*n* = 47), Ctrl (*n* = 27)	CSF (exosomes)/array and RT-qPCR	[171]
		AD (*n* = 30), Ctrl (*n* = 30)	CSF/RT-qPCR	[172]
		AD (*n* = 31), Ctrl (*n* = 29)	CNS (FCx)/RT-qPCR	[173]
		AD (*n* = 5), Ctrl (*n* = 5)	CNS (PCx)/array	[168]
	↑	AD (*n* = 10), VD (*n* = 4), FTD (*n* = 4), DLB (*n* = 2)	CSF/RT-qPCR	[174]
miR-34a(-5p)	↓	AD (*n* = 10), Ctrl (*n* = 10)	Plasma/RT-qPCR	[160]
		AD (*n* = 21/15), preclinical AD (*n* = 21/15), Ctrl (*n* = 21/15), PD (*n* = 21/0)	Plasma/RT-qPCR	[175]
		AD (*n* = 10), Ctrl (*n* = 10)	CSF/RT-qPCR	[160]
	↑	AD (*n* = 16), Ctrl (*n* = 16)	PBMC/array and RT-qPCR	[176]
		AD (*n* = 5), Ctrl (*n* = 5)	CNS (TCx)/RT-qPCR	[158]
		AD (*n* = 26), Ctrl (*n* = 19)	CNS (TCx)/array	[177]
		AD (*n* = 29), Ctrl (*n* = 20)	CNS (HPC)/RT-qPCR	[178]
		AD (*n* = 3), Ctrl (*n* = 3)	CNS (HPC)/array	[179]
miR-107	↓	AD (*n* = 48/106), MCI (*n* = 18/0), MS (*n* = 16/0), PD (*n* = 9/0), DEP (*n* = 15/0), BD (*n* = 15/0), SCH (*n* = 14/0), Ctrl (*n* = 22/22)	Blood/miRNA-seq and RT-qPCR	[180]
		AD (*n* = 97), aMCI (*n* = 116), Ctrl (*n* = 81)	Plasma/RT-qPCR	[181]
		AD (*n* = 27), Ctrl (*n* = 18)	CNS (TCx)/miRNA-seq and RT-qPCR	[154]
		AD (*n* = 6), MCI (*n* = 6), Ctrl (*n* = 11)	CNS (TCx)/array	[182]
		AD (*n* = 12), Ctrl (*n* = 12)	CNS (HPC, TCx, CB)/RT-qPCR	[183]
		AD (*n* = 10), Ctrl (*n* = 11)	CNS (HPC)/RT-qPCR	[184]
miR-125b	↓	AD (*n* = 69), PD (*n* = 67), Ctrl (*n* = 78)	Serum/miRNA-seq	[151]
		AD (*n* = 22), FTD (*n* = 10), Ctrl (*n* = 26)	Serum/array and RT-qPCR	[185]
		AD (*n* = 10), Ctrl (*n* = 10)	Plasma/RT-qPCR	[160]
		AD (*n* = 35), Ctrl (*n* = 35)	Plasma (exosomes)/miRNA-seq	[164]
		Probable AD (*n* = 105), Ctrl (*n* = 150)	Serum/RT-qPCR	[186]
		AD (*n* = 22), FTD (*n* = 10), Ctrl (*n* = 26)	CSF/array and RT-qPCR	[185]
		AD (*n* = 50/16), Ctrl (*n* = 49/16)	CSF/array and RT-qPCR	[161]
	↑	AD (*n* = 6), Ctrl (*n* = 6)	CSF/array and RT-qPCR	[156]
		AD (*n* = 10/37), Ctrl (*n* = 10/32)	CSF/array and RT-qPCR	[187]
		AD (*n* = 10); Ctrl (*n* = 10)	CSF/RT-qPCR	[160]
		YOAD (*n* = 17/17), LOAD (*n* = 13/13), Ctrl (*n* = 12/12)	CSF (exosomes)/RT-qPCR	[188]
miR-125b-3p	↓	AD (*n* = 20), Ctrl (*n* = 20)	Serum/miRNA-seq and RT-qPCR	[170]
	↑	AD (*n* = 6), Ctrl (*n* = 6)	CNS (TCx)/array and RT-qPCR	[156]
		AD (*n* = 27), Ctrl (*n* = 18)	CNS (TCx)/miRNA-seq and RT-qPCR	[154]
		AD (*n* = 5), Ctrl (*n* = 5)	CNS (TCx)/RT-qPCR	[158]
		AD (*n* = 26), Ctrl (*n* = 19)	CNS (TCx)/array	[177]
		AD (*n* = 6), Ctrl (*n* = 6)	CNS (TCx) array	[157]
		Braak III-VI (*n* = 20), Braak 0-I (*n* = 7)	CNS (HPC, MFG, CB)/RT-qPCR	[152]
		AD (*n* = 3), Ctrl *n* = 3)	CNS (HPC)/array	[179]
		AD (*n* = 10), Ctrl (*n* = 5)	CNS (FCx)/RT-qPCR	[167]
		AD (*n* = 9), MCI (*n* = 8), Ctrl (*n* = 10)	CNS/RT-qPCR	[189]
miR-132(-3p)	↓	AD (*n* = 31), AD-MCI (*n* = 16), Ctrl (*n* = 16)	Plasma (exosomes)/array and RT-qPCR	[190]
		AD (*n* = 11), Ctrl (*n* = 8)	CNS/array and RT-qPCR	[190]
		AD (*n* = 6), PD (*n* = 6), Ctrl (*n* = 6)	CNS (HPC, TCx, FCx)/miRNA-seq and RT-qPCR	[191]
		Braak III-VI (*n* = 20), Braak 0-I (*n* = 7)	CNS (HPC, MFG, CB)/RT-qPCR	[152]
		AD (*n* = 27), Ctrl (*n* = 18)	CNS (TCx)/miRNA-seq and RT-qPCR	[154]
		AD (*n* = 5), DLB (*n* = 4), FTD (*n* = 5), HS-aging (*n* = 4), Ctrl (*n* = 2)	CNS (TCx)/RNA deep seq and RT-qPCR	[192]
		HPC: AD (*n* = 41), Ctrl (*n* = 23); FCx: AD (*n* = 21), Ctrl (*n* = 28), TCx: AD (*n* = 8), Ctrl (*n* = 8)	CNS (HPC, FCx, TCx)/RNA deep seq and RT-qPCR	[193]
		FCx: AD (*n* = 225/8), Ctrl (*n* = 87/8), TCx: AD (*n* = 39/8), Ctrl (*n* = 25/8)	CNS (FCx, TCx)/array and RT-qPCR	[194]
		HPC: AD (*n* = 10), Ctrl (*n* = 13); FCx: AD (*n* = 7), Ctrl (*n* = 5); TCx: AD (*n* = 8/11), MCI (*n* = 0/10), Ctrl (*n* = 8/11)	CNS (HPC, FCx, TCx)/RT-qPCR	[195]
		AD (*n* = 3/10), MCI (*n* = 0/10), Ctrl (*n* = 3/12)	CNS (FCx)/array and RT-qPCR	[196]
		Braak IV (*n* = 18), Braak III/IV (*n* = 14), Ctrl (*n* = 18)	CNS (TCx)/RT-qPCR	[197]
	↑	MCI (*n* = 66), Ctrl (*n* = 76)	Serum/RT-qPCR	[198]
miR-146a(-5p)	↓	AD (*n* = 127), MCI (*n* = 30), VD (*n* = 30)	Serum/miRNA-seq and RT-qPCR	[199]
		AD (*n* = 10), Ctrl (*n* = 10)	Plasma/RT-qPCR	[160]
		AD (*n* = 40), Ctrl (*n* = 31); Validation cohort: publicly available dataset of miRNA data	Blood/miRNA-seq	[200]
		AD (*n* = 10), Ctrl (*n* = 10)	CSF/RT-qPCR	[160]
		AD (*n* = 50/16), Ctrl (*n* = 49/16)	CSF/array and RT-qPCR	[161]
		AD (*n* = 20), Ctrl (*n* = 20)	CSF/RT-qPCR	[184]
		AD (*n* = 60), MCI-AD (*n* = 39), FTD (*n* = 37), DLB (*n* = 37), Ctrl (*n* = 40)	CSF/RT-qPCR	[162]
		Braak III-VI (*n* = 20), Braak 0-I (*n* = 7)	CNS (HPC, MFG, CB)/RT-qPCR	[152]
		AD (*n* = 27), Ctrl (*n* = 18)	CNS (TCx)/miRNA-seq and RT-qPCR	[154]
		AD (*n* = 10), Ctrl (*n* = 11)	CNS (HPC)/array	[184]
		Braak III-VI (*n* = 20), Braak 0-I (*n* = 7)	CNS (HPC, MFG, CB)/RT-qPCR	[152]
		AD (*n* = 27), Ctrl (*n* = 18)	CNS (TCx)/miRNA-seq and RT-qPCR	[154]
	↑	AD (*n* = 20), Ctrl (*n* = 20)	Serum/miRNA-seq and RT-qPCR	[170]
		AD (*n* = 6), Ctrl (*n* = 6)	CSF/array and RT-qPCR	[156]
		AD (*n* = 22), Ctrl (*n* = 28)	CSF/RT-qPCR	[201]
		AD (*n* = 6), Ctrl (*n* = 6)	CNS (TCx)/array and RT-qPCR	[156]
		AD (*n* = 36), Ctrl (*n* = 30)	CNS (HPC, TCx)/array	[202]
		AD (*n* = 5), Ctrl (*n* = 5)	CNS (TCx)/RT-qPCR	[158]
		AD (*n* = 26), Ctrl (*n* = 19)	CNS (TCx)/array	[177]
		AD (*n* = 6), Ctrl (*n* = 6)	CNS (TCx)/array	[157]
		AD (*n* = 12), Ctrl (*n* = 6)	CNS/array	[203]
		AD (*n* = 23), Ctrl (*n* = 23)	CNS (TCx)/array	[204]
		AD (*n* = 3), Ctrl (*n* = 3)	CNS (HPC)/array	[179]
miR-155	↑	AD (*n* = 36), MCI (*n* = 52), Ctrl (*n* = 6)	PBMC/RT-qPCR	[205]
		AD (*n* = 16), Ctrl (*n* = 16)	PBMC/array and RT-qPCR	[176]
		AD (*n* = 6), Ctrl (*n* = 6)	CNS (TCx)/array and RT-qPCR	[156]
		AD (*n* = 12), Ctrl (*n* = 6)	CNS/array	[203]
		AD (*n* = 5), Ctrl (*n* = 5)	CNS (TCx)/RT-qPCR	[158]
		AD (*n* = 3), Ctrl (*n* = 3)	CNS (HPC)/array	[179]
		Braak III-VI (*n* = 20), Braak 0-I (*n* = 7)	CNS (HPC, MFG, CB)/RT-qPCR	[152]
		AD (*n* = 27), Ctrl (*n* = 18)	CNS (TCx)/miRNA-seq and RT-qPCR	[154]
		AD (*n* = 26), Ctrl (*n* = 19)	CNS (TCx)/array	[177]
miR-181c(-5p)	↓	Probable AD (*n* = 7), aMCI/Probable Early AD (*n* = 7), Ctrl (*n* = 7)	Serum/RT-qPCR	[163]
		Probable AD (*n* = 105), Ctrl (*n* = 150)	Serum/RT-qPCR	[186]
		AD (*n* = 7), Ctrl (*n* = 7)	CNS (neocortex)/RT-qPCR	[153]
		AD (*n* = 5), Ctrl (*n* = 5)	CNS (PCx)/array	[168]
	↑	AD (*n* = 56/0), MCI (*n* = 26/0), FTD (*n* = 0/27), Ctrl (*n* = 14/24)	Plasma/RT-qPCR	[206]
miR-206	↑	AD (*n* = 25), MCI (*n* = 30), Ctrl (*n* = 31); Longitudinal cohort: MCI-MCI-Dementia (*n* = 6), Ctrl-MCI-Dementia (*n* = 6), Ctrl-MCI-MCI (*n* = 6)	Plasma/array and RT-qPCR	[207]
		MCI (*n* = 66), Ctrl (*n* = 76)	Serum/RT-qPCR	[198]
		AD (*n* = 10/18), Ctrl (*n* = 10/18)	CSF/RT-qPRC	[155]
		AD (*n* = 19/19); Ctrl (*n* = 19/19)	CSF/array and RT-qPCR	[208]
miR-212(-3p)	↓	AD (*n* = 31), AD-MCI (*n* = 16), Ctrl (*n* = 16)	Plasma (exosomes)/array and RT-qPCR	[190]
		AD (*n* = 11), Ctrl (*n* = 8)	CNS/Array & RT-qPCR	[190]
		AD (*n* = 6), PD (*n* = 6), Ctrl (*n* = 6)	CNS (HPC, FCx, TCx)/miRNA-seq and RT-qPCR	[191]
		Braak III-VI (*n* = 20), Braak 0-I (*n* = 7)	CNS (HPC, MFG, CB)/RT-qPCR	[152]
		AD (*n* = 3/10), MCI (*n* = 10), Ctrl (*n* = 3/12)	CNS (FCx)/array and RT-qPCR	[196]
		Braak IV (*n* = 18), Braak III/IV (*n* = 14), Ctrl (*n* = 18)	CNS (TCx)/RT-qPCR	[197]
		FCX: AD (*n* = 225/8), Ctrl (*n* = 87/8); TCx: AD (*n* = 39/8), Ctrl (*n* = 25/8)	CNS (FCx, TCx)/array and RT-qPCR	[194]
		AD (*n* = 27), Ctrl (*n* = 18)	CNS (TCx)/miRNA-seq and RT-qPCR	[154]

↓—decreased levels; ↑—increased levels; AD—Alzheimer’s disease; BD—bipolar disorder; CB—cerebellum; CNS—central nervous system; Ctrl—control subjects; DEP—depression; DLB—dementia with Lewy bodies; FCx—frontal cortex; FTD—frontotemporal dementia; HPC—hippocampus; HS-aging—hippocampal sclerosis of aging; LOAD—late onset AD; MCI—mild cognitive impairment; MFG—medial frontal gyrus; aMCI—amnestic MCI; MS—multiple sclerosis; n—number of subjects; PBMC—peripheral blood mononuclear cell; PD—Parkinson’s disease; PCx—parietal cortex; RT-qPCR—reverse transcription quantitative real-time PCR; SCH—schizophrenia; SMC—subjective memory complaints; TCx—temporal cortex; VD—vascular dementia; YOAD—young onset AD. ^1^ Number of subjects per group is presented as discovery cohort/validation cohort.

## Supplementary Materials

The following are available online at https://www.mdpi.com/2218-273X/11/2/195/s1, Table S1: Overview of studies investigating miRNA expression in AD.
