# Supplementary material for: Epigenetics of Alzheimer’s Disease
Source: Biomolecules. 2021 Jan 30;11(2):195. doi: 10.3390/biom11020195 (PMC7911414; doi:10.3390/biom11020195)
Supplement: Supplementary file 1 [file biomolecules-11-00195-s001.pdf]

biomolecules

MDPI

Review

Epigenetics of Alzheimer's Disease

Matea Nikolac Perkovic [1,†], Alja Videtic Paska [2,†], Marcela Konjevod [1], Katarina Kouter [2], Dubravka Svob Strac [1], Gordana Nedic Erjavec [1] and Nela Pivac [1,*]

[1] Laboratory for Molecular Neuropsychiatry, Division of Molecular Medicine, Ruder Boskovic Institute, HR-10000 Zagreb, Croatia; mnikolac@irb.hr (M.N.P.); mkonjev@irb.hr (M.K.); dsvob@irb.hr (D.S.S.); gnedic@irb.hr (G.N.E.)

[2] Medical Center for Molecular Biology, Institute of Biochemistry and Molecular Genetics, Faculty of Medicine, University of Ljubljana, SI-1000 Ljubljana, Slovenia; alja.videtic@mf.uni-lj.si (A.V.P.); katarina.kouter@mf.uni-lj.si (K.K.)

* Correspondence: npivac@irb.hr; Tel.: +38-514-571-207

† M.N.P. and A.V.P. equally contributed to this work.

Abstract: There are currently no validated biomarkers, which can be used to accurately diagnose Alzheimer's disease (AD), and to distinguish it from other dementia-causing neuropathologies. Moreover, to date, only symptomatic treatments exist for this progressive neurodegenerative disorder. In the search for new, more reliable biomarkers, and potential therapeutic options, epigenetic modifications have emerged as important players in the pathogenesis of AD. The aim of the article was to provide a brief overview of the current knowledge regarding the role of epigenetics (including mitoepigenetics) in AD, and the possibility of applying these advances for future AD therapy. Extensive research has suggested an important role of DNA methylation and hydroxymethylation, histone posttranslational modifications, and noncoding RNA regulation (with the emphasis on microRNAs) in the course and development of AD. Recent studies also indicated mitochondrial DNA (mtDNA) as an interesting biomarker of AD, since dysfunctions in the mitochondria, as well as lower mtDNA copy number, have been associated with AD pathophysiology. The current evidence suggest that epigenetic changes can be successfully detected, not only in the central nervous system, but also in the cerebrospinal fluid and on the periphery, contributing further to their potential as both biomarkers and therapeutic targets in AD.

Keywords: epigenetics; mitoepigenetics; DNA methylation; DNA hydroxymethylation; miRNA; histone modifications; Alzheimer's disease

---

Table S1. Overview of studies investigating miRNA expression in AD.

Epigenetic mechanism	Effect	Gene/Target pathway involved [1]	Study model [2]	Tissue Study/design	Main results	References
**let-7b**	↑	http://www.mirdb.org/cgi-bin/search.cgi?searchType=miRNA&searchBox=hsa-let-7b-5p&full=1	AD (*n* = 25), MCI (*n* = 30), Ctrl (*n* = 31); Longitudinal cohort: MCI-MCI-Dementia (*n* = 6), Ctrl-MCI-Dementia (*n* = 6), Ctrl-MCI-MCI (*n* = 6)	Plasma Array/RT-qPCR	Expression of let-7b increased in MCI subjects. Expression of miR-206 increased in AD subjects.	[217]
			AD (*n* = 17), SMC (*n* = 41), MCI (*n* = 36)	CSF RT-qPCR	Expression of let-7b increased in association with the progression of AD.	[290]
**let-7d-5p**	↓	http://www.mirdb.org/cgi-bin/search.cgi?searchType=miRNA&searchBox=hsa-let-7d-5p&full=1	AD (*n* = 11/20), MCI (*n* = 9/0), Ctrl (*n* = 20/17)	Plasma miRNA-seq/RT-qPCR	Expression of let-7d-5p, let-7g-5p, miR-15b-5p, miR-142-3p, miR-191-5p, miR-301a-3p and miR-545-3p deregulated in AD.	[291]
**let-7f-5p**	↑	http://www.mirdb.org/cgi-bin/search.cgi?searchType=miRNA&searchBox=hsa-let-7f-5p&full=1	AD (*n* = 27), Ctrl (*n* = 18)	Serum miRNA-seq/RT-qPCR	Expression of 20 miRNAs deregulated in AD, including miR-501-3p, let-7f-5p, and miR-26b-5p.	[167]

Epigenetic mechanism	Effect	Gene/Target pathway involved [1]	Study model [2]	Tissue Study/design	Main results	References
**let-7g-5p**	↓	http://www.mirdb.org/cgi-bin/search.cgi?searchType=miRNA&searchBox=hsa-let-7g-5p&full=1	AD (*n* = 11/20), MCI (*n* = 9/0), Ctrl (*n* = 20/17)	Plasma<br>miRNA-seq/RT-qPCR	Expression of let-7d-5p, let-7g-5p, miR-15b-5p, miR-142-3p, miR-191-5p, miR-301a-3p and miR-545-3p deregulated in AD.	[291]
**let-7i-5p**	↓	http://www.mirdb.org/cgi-bin/search.cgi?searchType=miRNA&searchBox=hsa-let-7i-5p&full=1	Aβ(−)MCI (*n* = 12), Aβ(+)MCI (*n* = 11), Ctrl (*n* = 9)	Platelets<br>Array/RT-qPCR	Expression of let-7i-5p, miR-125a, miR-1233-5p, and miR-6787-5p downregulated in AD, miR-6880-5p upregulated in AD.	[292]
**miR-9-5p**	↓	http://www.mirdb.org/cgi-bin/search.cgi?searchType=miRNA&searchBox=hsa-miR-9-5p&full=1	AD (*n* = 69), PD (*n* = 67), Ctrl (*n* = 78)	CSF<br>miRNA-seq	Expression of 41 miRNAs deregulated in AD CSF, and 20 miRNAs in AD serum.	[164]
			Braak III-VI (*n* = 20), Braak 0-I (*n* = 7)	CNS (HPC, MFG, CB)<br>RT-qPCR	Expression of miR-145, miR-148a, miR-200c, miR-210, miR-26a, miR-30c, miR-30e-5p, miR-423, and miR-92 deregulated in HPC and/or MFG. Expression of miR-100, miR-125b, miR-132, miR-146b, miR-212, miR-27a, miR-27b, miR-34a, miR-381, miR-422a, miR-425, miR-9 deregulated in CB. Increased expression of miR-29a and miR-29b.	[165]

Epigenetic mechanism	Effect	Gene/Target pathway involved [1]	Study model [2]	Tissue Study/design	Main results	References
	↓		AD (*n* = 7), Ctrl (*n* = 7)	CNS (Neocortex)<br>RT-qPCR	Expression of miR-137, miR-181c, miR-9, miR-29a, miR-29b-1, miR-15 and miR-124 downregulated in AD FCx.	[166]
			AD (*n* = 27), Ctrl (*n* = 18)	CNS (TCx)<br>miRNA-seq/RT-qPCR	Expression of 20 miRNAs deregulated in AD, including miR-501-3p, let-7f-5p, and miR-26b-5p.	[167]
**miR-9-5p**	↑	http://www.mirdb.org/cgi-bin/search.cgi?searchType=miRNA&searchBox=hsa-miR-9-5p&full=1	AD (*n* = 10/18), Ctrl (*n* = 10/18)	CSF<br>RT-qPRC	Expression of 15 microRNAs deregulated in AD. miR-9-5p and miR-598 suggested as potential biomarkers for AD.	[168]
			AD (*n* = 6), Ctrl (*n* = 6)	CNS (TCx)<br>Array/RT-qPCR	Expression of miRNA-9, miRNA-125b, miRNA-146a, miRNA-155 increased in AD CSF and brain tissue-derived extracellular fluid.	[169]
			AD (*n* = 6), Ctrl (*n* = 6)	CNS (TCx)<br>Array	Expression of miRNA-9, miRNA-125b and miRNA-146a up-regulated in AD TCx.	[170]

Epigenetic mechanism	Effect	Gene/Target pathway involved [1]	Study model [2]	Tissue Study/design	Main results	References
**miR-9-5p**	↑	http://www.mirdb.org/cgi-bin/search.cgi?searchType=miRNA&searchBox=hsa-miR-9-5p&full=1	AD (*n* = 5), Ctrl (*n* = 5)	CNS (TCx) RT-qPCR	Expression of miR-146a, miR-155, miR-9, miR-125b, miR-34a, miR-25, miR-128, miR-15b, miR-454, miR-642, miR-20a, and miR-106a increased in AD neocortical extracellular fluid and CSF.	[171]
			AD (*n* = 5), Ctrl (*n* = 5)	CNS (HPC) Array	Expression of miR-9, miR-125b and miR-128 elevated and of miR-124a reduced in AD.	[172]
**miR-15b-5p**	↓	http://www.mirdb.org/cgi-bin/search.cgi?searchType=miRNA&searchBox=hsa-miR-15b-5p&full=1	AD (*n* = 23/16), MCI (*n* = 3/8), Ctrl (*n* = 23/36)	Serum (Exosomes) miRNA-seq/RT-qPCR	Expression of 17 miRNA deregulated (14 miRNAs upregulated and 3 downregulated) in AD.	[293]
			AD (*n* = 11/20), MCI (*n* = 9/0), Ctrl (*n* = 20/17)	Plasma miRNA-seq/RT-qPCR	Expression of let-7d-5p, let-7g-5p, miR-15b-5p, miR-142-3p, miR-191-5p, miR-301a-3p and miR-545-3p deregulated in AD.	[291]
			AD (*n* = 40), Ctrl (*n* = 31); Validation cohort: publicly available dataset of miRNA data	Blood miRNA-seq	Expression of 71 deregulated in AD, including miR-146b-5p and miR-15b-5p.	[210]

Epigenetic mechanism	Effect	Gene/Target pathway involved [1]	Study model [2]	Tissue Study/design	Main results	References
	↓		AD (*n* = 10), Ctrl (*n* = 11)	CNS (HPC) RT-qPCR	Expression of miR-16, miR-34c, miR-107, miR-128a, and miR-146a were differentially regulated in AD HPC.	[196]
**miR-16-5p**		http://www.mirdb.org/cgi-bin/search.cgi?searchType=miRNA&searchBox=hsa-miR-16-5p&full=1	Braak III-VI (*n* = 20), Braak 0-I (*n* = 7)	CNS (HPC, MFG, CB) RT-qPCR	Expression of miR-145, miR-148a, miR-200c, miR-210, miR-26a, miR-30c, miR-30e-5p, miR-423, and miR-92 deregulated in HPC and/or MFG. Expression of miR-100, miR-125b, miR-132, miR-146b, miR-212, miR-27a, miR-27b, miR-34a, miR-381, miR-422a, miR-425, miR-9 deregulated in CB. Increased expression of miR-29a and miR-29b.	[165]
			AD (*n* = 27), Ctrl (*n* = 18)	CNS (TCx) miRNA-seq/RT-qPCR	Expression of 20 miRNAs deregulated in AD, including miR-501-3p, let-7f-5p, and miR-26b-5p.	[167]

Epigenetic mechanism	Effect	Gene/Target pathway involved ¹	Study model ²	Tissue Study/design	Main results	References
**miR-16-5p**	↓	http://www.mirdb.org/cgi-bin/search.cgi?searchType=miRNA&searchBox=hsa-miR-16-5p&full=1	AD (*n* = 12), Ctrl (*n* = 12)	CNS (HPC, TCx, CB)<br>RT-qPCR	Expression of miR-103, miR-107, miR-15b, miR-16 and miR-195 reduced in AD HPC and TCx (including miR-15a), but not in CB.	[195]
**miR-19b-3p**	↓	http://www.mirdb.org/cgi-bin/search.cgi?searchType=miRNA&searchBox=hsa-miR-19b-3p&full=1	AD (*n* = 20), Ctrl (*n* = 20)	Serum<br>miRNA-seq/RT-qPCR	Expression of 72 miRNAs deregulated in AD (miR-146a-5p, miR-106b-3p, miR-195-5p, miR-20b-5p, and miR-497-5p upregulated, miR-125b-3p, miR-29c-3p, miR-93-5p and miR-19b-3p downregulated).	[182]
**miR-22-3p**	↑	http://www.mirdb.org/cgi-bin/search.cgi?searchType=miRNA&searchBox=hsa-miR-22-3p&full=1	AD (*n* = 19/121), Ctrl (*n* = 9/86)	Serum<br>miRNA-seq/RT-qPCR	Expression of miR-26a-5p, miR-181c-3p, miR-126-5p, miR-22-3p, miR-148b-5p, miR-106b-3p, miR-6119-5p, miR-1246, and miR-660-5p can be utilized as biomarker for detecting AD.	[294]
**miR-27a-3p**	↓	http://www.mirdb.org/cgi-bin/search.cgi?searchType=miRNA&searchBox=hsa-miR-27a-3p&full=1	AD (*n* = 20/15), Ctrl (*n* = 19/18)	CSF<br>RT-qPCR	Expression of miR-27a-3p level reduced in AD CSF.	[295]

Epigenetic mechanism	Effect	Gene/Target pathway involved [1]	Study model [2]	Tissue Study/design	Main results	References
**miR-29a(-3p)**	↓		AD (*n* = 10), Ctrl (*n* = 10)	CSF RT-qPCR	Expression of miR-34a and miR-146a decreased in AD plasma, and of miR-34a, miR-125b, and miR-146a in CSF.	[173]
		http://www.mirdb.org/cgi-bin/search.cgi?searchType=miRNA&searchBox=hsa-miR-29a-3p&full=1	AD (*n* = 50/16); Ctrl (*n* = 49/16)	CSF Array/RT-qPCR	Expression of 36 miRNAs (20 in validation cohort) discriminate AD from control CSF.	[174]
		http://www.mirdb.org/cgi-bin/search.cgi?searchType=miRNA&searchBox=hsa-miR-29a-3p&full=1	AD (*n* = 7), Ctrl (*n* = 7)	CNS (Neocortex) RT-qPCR	Expression of miR-137, miR-181c, miR-9, miR-29a, miR-29b-1, miR-15 and miR-124 downregulated in AD FCx.	[166]
	↑		AD (*n* = 18), Ctrl (*n* = 20)	CSF RT-qPCR	Expression of miR-29a increased in AD CSF.	[175]

Epigenetic mechanism	Effect	Gene/Target pathway involved [1]	Study model [2]	Tissue Study/design	Main results	References
**miR-29a(-3p)**	↑	http://www.mirdb.org/cgi-bin/search.cgi?searchType=miRNA&searchBox=hsa-miR-29a-3p&full=1	Braak III-VI (*n* = 20), Braak 0-I (*n* = 7)	CNS (HPC, MFG, CB)<br>RT-qPCR	Expression of miR-145, miR-148a, miR-200c, miR-210, miR-26a, miR-30c, miR-30e-5p, miR-423, and miR-92 deregulated in HPC and/or MFG. Expression of miR-100, miR-125b, miR-132, miR-146b, miR-212, miR-27a, miR-27b, miR-34a, miR-381, miR-422a, miR-425, miR-9 deregulated in CB. Increased expression of miR-29a and miR-29b.	[165]
**miR-29b(-3p)**	↓	http://www.mirdb.org/cgi-bin/search.cgi?searchType=miRNA&searchBox=hsa-miR-29b-3p&full=1	Probable AD (*n* = 7), aMCI/Probable Early AD (*n* = 7), Ctrl (*n* = 7)	Serum<br>RT-qPCR	Expression of miR-137, miR-181c, miR-9, miR-29a/b down-regulated in probable AD patients.	[176]
			AD (*n* = 35), Ctrl (*n* = 35)	Plasma (Exosomes)<br>miRNA-seq	Expression of 20 miRNAs deregulated in AD group.	[177]
			AD (*n* = 48), Ctrl (*n* = 22)	BloodomiRas and DIANA miRPath	Expression of 27 miRNAs discriminate between two groups.	[178]
			AD (*n* = 28), Ctrl (*n* = 25)	PBMC<br>RT-qPCR	Expression of miR-29b downregulated in AD.	[179]

Epigenetic mechanism	Effect	Gene/Target pathway involved [1]	Study model [2]	Tissue Study/design	Main results	References
	↓		AD (*n* = 10), Ctrl (*n* = 5)	CNS (FCx) RT-qPCR	Expression of miR-125b increased in AD brains. Expression of miR-29a and 29b downregulated in AD.	[158]
			AD (*n* = 5), Ctrl (*n* = 5)	CNS (PCx) Array	Expression of 48 miRNAs differentially regulated in AD brain.	[180]
**miR-29b(-3p)**	↑	http://www.mirdb.org/cgi-bin/search.cgi?searchType=miRNA&searchBox=hsa-miR-29b-3p&full=1	Braak III–VI (*n* = 20), Braak 0–I (*n* = 7)	CNS (HPC, MFG, CB) RT-qPCR	Expression of miR-145, miR-148a, miR-200c, miR-210, miR-26a, miR-30c, miR-30e-5p, miR-423, and miR-92 deregulated in HPC and/or MFG. Expression of miR-100, miR-125b, miR-132, miR-146b, miR-212, miR-27a, miR-27b, miR-34a, miR-381, miR-422a, miR-425, miR-9 deregulated in CB. Increased expression of miR-29a and miR-29b.	[165]
**miR-29c(-3p)**	↓	http://www.mirdb.org/cgi-bin/search.cgi?searchType=miRNA&searchBox=hsa-miR-29c-3p&full=1	AD (*n* = 30), Ctrl (*n* = 30)	Blood RT-qPCR	Expression of miR-29c decreased in AD.	[181]

Epigenetic mechanism	Effect	Gene/Target pathway involved [1]	Study model [2]	Tissue Study/design	Main results	References
	↓	http://www.mirdb.org/cgi-bin/search.cgi?searchType=miRNA&searchBox=hsa-miR-29c-3p&full=1	AD (*n* = 20), Ctrl (*n* = 20)	Serum miRNA-seq/RT-qPCR	Expression of 72 miRNAs deregulated in AD (miR-146a-5p, miR-106b-3p, miR-195-5p, miR-20b-5p, and miR-497-5p upregulated, miR-125b-3p, miR-29c-3p, miR-93-5p and miR-19b-3p downregulated).	[182]
			AD (*n* = 28), PD (*n* = 47), Ctrl (*n* = 27)	CSF (Exosomes) Array/RT-qPCR	Expression of miR-16-2, miR-331-5p, miR-132-5p, miR-485-5p, miR-151, miR-136-3p, and miR-29c were deregulated in AD CSF.	[183]
**miR-29c(-3p)**			AD (*n* = 30), Ctrl (*n* = 30)	CSF RT-qPCR	Expression of miR-29c decreased in AD CSF.	[184]
			AD (*n* = 31), Ctrl (*n* = 29)	CNS (FCx) RT-qPCR	Expression of miR-29c downregulated in sporadic AD brains.	[185]
			AD (*n* = 5), Ctrl (*n* = 5)	CNS (PCx) Array	Expression of 48 miRNA deregulated in AD brains.	[180]
	↑		AD (*n* = 10), VD (*n* = 4), FTD (*n* = 4), DLB (*n* = 2)	CSF RT-qPCR	Expression of let-7i-5p and miR-15a-5p upregulated and of miR-29c-3p downregulated in AD.	[186]

Epigenetic mechanism	Effect	Gene/Target pathway involved [1]	Study model [2]	Tissue Study/design	Main results	References
**miR-30a-3p**	↓	http://www.mirdb.org/cgi-bin/search.cgi?searchType=miRNA&searchBox=hsa-miR-30a-3p&full=1	AD (*n* = 50/16), Ctrl (*n* = 49/16)	CSF Array/RT-qPCR	Expression of 36 miRNAs (20 in validation cohort) discriminate AD from control CSF.	[174]
	↑		Braak V (*n* = 10), Braak I (*n* = 10)	CSF RT-qPCR	Expression of miR-145, miR-148a, miR-200c, miR-210, miR-26a, miR-30c, miR-30e-5p, miR-423, and miR-92 deregulated in HPC and/or MFG. Expression of miR-100, miR-125b, miR-132, miR-146b, miR-212, miR-27a, miR-27b, miR-34a, miR-381, miR-422a, miR-425, miR-9 deregulated in CB. Increased expression of miR-29a and miR-29b.	[165]
**miR-34a(-5p)**	↓	http://www.mirdb.org/cgi-bin/search.cgi?searchType=miRNA&searchBox=hsa-miR-34a-5p&full=1	AD (*n* = 10), Ctrl (*n* = 10)	Plasma RT-qPCR	Expression of miR-34a and miR-146a decreased in AD plasma, and of miR-34a, miR-125b, and miR-146a in CSF.	[173]
			AD (*n* = 21/15), preclinical AD (*n* = 21/15), Ctrl (*n* = 21/15), PD (*n* = 21/0)	Plasma RT-qPCR	Expression levels of miR-15b-5p, miR-34a-5p, miR-142-3p, and miR-545-3p distinguished AD from PD and HC subjects.	[187]

Epigenetic mechanism	Effect	Gene/Target pathway involved [1]	Study model [2]	Tissue Study/design	Main results	References
	↓		AD (*n* = 10), Ctrl (*n* = 10)	CSF RT-qPCR	Expression of miR-34a and miR-146a decreased in AD plasma, and of miR-34a, miR-125b, and miR-146a in CSF.	[173]
	↑		AD (*n* = 16), Ctrl (*n* = 16)	PBMC Array/RT-qPCR	Expression of 22 miRNAs upregulated in AD subjects.	[188]
**miR-34a(-5p)**		http://www.mirdb.org/cgi-bin/search.cgi?searchType=miRNA&searchBox=hsa-miR-34a-5p&full=1	AD (*n* = 5), Ctrl (*n* = 5)	CNS (TCx) RT-qPCR	Expression of miR-146a, miR-155, miR-9, miR-125b, miR-34a, miR-25, miR-128, miR-15b, miR-454, miR-642, miR-20a, and miR-106a increased in AD neocortical extracellular fluid and CSF samples.	[171]
			AD (*n* = 26), Ctrl (*n* = 19)	CNS (TCx) Array	Expression of miR-7, miR-9-1, miR-23a/miR-27a, miR-34a, miR-125b-1, miR-146a, and miR-155 increased in AD-affected superior TCx.	[189]

Epigenetic mechanism	Effect	Gene/Target pathway involved [1]	Study model [2]	Tissue Study/design	Main results	References
**miR-34a(-5p)**	↑	http://www.mirdb.org/cgi-bin/search.cgi?searchType=miRNA&searchBox=hsa-miR-34a-5p&full=1	AD (*n* = 29), Ctrl (*n* = 20)	CNS (HPC) RT-qPCR	Expression of miR-34a increased in the AD HPC.	[190]
			AD (*n* = 3), Ctrl (*n* = 3)	CNS (HPC) Array	Expression of miR-34a, miR-125b, miR-146a, miR-155 upregulated in AD brain samples.	[170]
**miR-34c**	↑	http://www.mirdb.org/cgi-bin/search.cgi?searchType=miRNA&searchBox=hsa-miR-34c-5p&full=1	AD (*n* = 110), Ctrl (*n* = 123)	Plasma, PBMC RT-qPCR	Expression of miR-34c increased in both AD PBMCs and plasma.	[296]
			aMCI (*n* = 71), Ctrl (*n* = 69)	Plasma RT-qPCR	Expression of miR-34c increased in patients with aMCI.	[297]
**miR-92a-3p**	↑	http://www.mirdb.org/cgi-bin/search.cgi?searchType=miRNA&searchBox=hsa-miR-92a-3p&full=1	AD (*n* = 56/0), MCI (*n* = 26/0), FTD (*n* = 0/27), Ctrl (*n* = 14/24)	Plasma RT-qPCR	Expression of miR-92a-3p, miR-181c-5p and miR-210-3p increased in AD and MCI plasma.	[216]
**miR-98-5p**	↓	http://www.mirdb.org/cgi-bin/search.cgi?searchType=miRNA&searchBox=hsa-miR-98-5p&full=1	AD (*n* = 50/158), Ctrl (*n* = 50/155)	Serum miRNA-seq/RT-qPCR	Expression of miR-98-5p, miR-885-5p, miR-483-3p, miR-342-3p, miR-191-5p, and miR-let-7d-5p deregulated in AD patients.	[198]

Epigenetic mechanism	Effect	Gene/Target pathway involved [1]	Study model [2]	Tissue Study/design	Main results	References
**miR-103a-3p**	↓	http://www.mirdb.org/cgi-bin/search.cgi?searchType=miRNA&searchBox=hsa-miR-103a-3p&full=1	AD (*n* = 48/106), MCI (*n* = 18/0), MS (*n* = 16/0), PD (*n* = 9/0), DEP (*n* = 15/0), BD (*n* = 15/0), SCH (*n* = 14/0), Ctrl (*n* = 22/22)	Blood<br>miRNA-seq/RT-qPCR	Expression of 82 miRNAs upregulated and 58 downregulated in AD patient samples.	[192]
			AD (*n* = 7/13), MCI-AD (*n* = 7/8), Ctrl (*n* = 6/9)	Plasma<br>RT-qPCR	Expression of miR-151a-5p, miR-30b-5p, miR-486-5p, miR-33a-5p, miR-483-5p, miR-18a-5p, miR-320a, miR-320b, miR-320c, miR-502-3p, miR-103a-3p, miR-301a-3p, miR-142-3p, and miR-200a-3p deregulated in AD.	[298]
**miR-106b**	↓	http://www.mirdb.org/cgi-bin/search.cgi?searchType=miRNA&searchBox=hsa-miR-106b-5p&full=1	AD (*n* = 56), Ctrl (*n* = 60)	Serum<br>RT-qPCR	Expression of miR-106b downregulated in AD serum samples.	[299]
**miR-106b-3p**	↑	http://www.mirdb.org/cgi-bin/search.cgi?searchType=miRNA&searchBox=hsa-miR-106b-3p&full=1	AD (*n* = 20), Ctrl (*n* = 20)	Serum<br>miRNA-seq/RT-qPCR	Expression of 72 miRNAs deregulated in AD (miR-146a-5p, miR-106b-3p, miR-195-5p, miR-20b-5p, and miR-497-5p upregulated, miR-125b-3p, miR-29c-3p, miR-93-5p and miR-19b-3p downregulated).	[182]

Epigenetic mechanism	Effect	Gene/Target pathway involved [1]	Study model [2]	Tissue Study/design	Main results	References
	↓		AD (*n* = 48/106), MCI (*n* = 18/0), MS (*n* = 16/0), PD (*n* = 9/0), DEP (*n* = 15/0), BD (*n* = 15/0), SCH (*n* = 14/0), Ctrl (*n* = 22/22)	Blood<br>miRNA-seq/RT-qPCR	Expression of 82 miRNAs upregulated and 58 downregulated in AD patient samples.	[192]
			AD (*n* = 97), aMCI (*n* = 116), Ctrl (*n* = 81)	Plasma<br>RT-qPCR	Expression of miR-107 in plasma had a high capability to discriminate between patients with aMCI and healthy controls.	[193]
**miR-107**		http://www.mirdb.org/cgi-bin/search.cgi?searchType=miRNA&searchBox=hsa-miR-107&full=1	AD (*n* = 27), Ctrl (*n* = 18)	CNS (TCx)<br>miRNA-seq/RT-qPCR	Expression of 20 miRNAs deregulated in AD, including miR-501-3p, let-7f-5p, and miR-26b-5p.	[167]
			AD (*n* = 6), MCI (*n* = 6), Ctrl (*n* = 11)	CNS (TCx)<br>Array	Expression of miR-107 decreased in the progression of AD.	[194]
			AD (*n* = 12), Ctrl (*n* = 12)	CNS (HPC, TCx, CB)<br>RT-qPCR	Expression of miR-103, miR-107, miR-15b, miR-16 and miR-195 reduced in AD HPC and TCx (including miR-15a), but not in CB.	[195]

Epigenetic mechanism	Effect	Gene/Target pathway involved [1]	Study model [2]	Tissue Study/design	Main results	References
**miR-107**	↓	http://www.mirdb.org/cgi-bin/search.cgi?searchType=miRNA&searchBox=hsa-miR-107&full=1	AD (*n* = 10), Ctrl (*n* = 11)	CNS (HPC)<br>RT-qPCR	Expression of miR-16, miR-34c, miR-107, miR-128a, and miR-146a were differentially regulated in AD HPC.	[196]
**miR-125a-5p**	↑	http://www.mirdb.org/cgi-bin/search.cgi?searchType=miRNA&searchBox=hsa-miR-125a-5p&full=1	AD (*n* = 27), Ctrl (*n* = 18)	CNS (TCx)<br>miRNA-seq/RT-qPCR	Expression of 20 miRNAs deregulated in AD, including miR-501-3p, let-7f-5p, and miR-26b-5p.	[167]
**miR-125b**	↓	http://www.mirdb.org/cgi-bin/search.cgi?searchType=miRNA&searchBox=hsa-miR-125b-5p&full=1	AD (*n* = 69), PD (*n* = 67), Ctrl (*n* = 78)	Serum<br>miRNA-seq	Expression of 41 miRNAs deregulated in AD CSF, and 20 miRNAs in AD serum.	[164]
			AD (*n* = 22), FTD (*n* = 10), Ctrl (*n* = 26)	Serum<br>Array/RT-qPCR	Expression of miR-26b, miR-23a and miR-126b downregulated in AD serum, and of miR-26b and miR-126b in AD CSF.	[197]
			AD (*n* = 10), Ctrl (*n* = 10)	Plasma<br>RT-qPCR	Expression of miR-34a and miR-146a decreased in AD plasma, and of miR-34a, miR-125b, and miR-146a in CSF.	[173]

Epigenetic mechanism	Effect	Gene/Target pathway involved [1]	Study model [2]	Tissue Study/design	Main results	References
	↓		AD (*n* = 35), Ctrl (*n* = 35)	Plasma (Exosomes) miRNA-seq	Expression of 20 miRNAs deregulated in AD group.	[177]
			Probable AD (*n* = 105), Ctrl (*n* = 150)	Serum RT-qPCR	Expression of miR-125b and miR-181c downregulated, and of miR-9 upregulated in serum of AD patients.	[198]
			AD (*n* = 22), FTD (*n* = 10), Ctrl (*n* = 26)	CSF Array/RT-qPCR	Expression of miR-26b, miR-23a and miR-126b downregulated in AD serum, and of miR-26b and miR-126b in AD CSF.	[197]
**miR-125b**		http://www.mirdb.org/cgi-bin/search.cgi?searchType=miRNA&searchBox=hsa-miR-125b-5p&full=1	AD (*n* = 50/16), Ctrl (*n* = 49/16)	CSF Array/RT-qPCR	Expression of 36 miRNAs (20 in validation cohort) discriminate AD from control CSF.	[174]
	↑		AD (*n* = 6), Ctrl (*n* = 6)	CSF Array/RT-qPCR	Expression of miRNA-9, miRNA-125b, miRNA-146a, miRNA-155 increased in AD CSF and brain tissue-derived extracellular fluid.	[169]
			AD (*n* = 10/37), Ctrl (*n* = 10/32)	CSF Array/RT-qPCR	Expression of miR-222 and miR-125b upregulated in AD CSF.	[199]

Epigenetic mechanism	Effect	Gene/Target pathway involved [1]	Study model [2]	Tissue Study/design	Main results	References
**miR-125b**	↑	http://www.mirdb.org/cgi-bin/search.cgi?searchType=miRNA&searchBox=hsa-miR-125b-5p&full=1	AD (*n* = 10); Ctrl (*n* = 10)	CSF<br>RT-qPCR	Expression of miR-34a and miR-146a decreased in AD plasma, and of miR-34a, miR-125b, and miR-146a in CSF.	[173]
			YOAD (*n* = 17/17), LOAD (*n* = 13/13), Ctrl (*n* = 12/12)	CSF (Exosomes)<br>RT-qPCR	Expression of miR-16-5p, miR-125b-5p, miR-451a, and miR-605-5p deregulated in AD CSF-derived exosomes.	[200]
**miR-125b-3p**	↓	http://www.mirdb.org/cgi-bin/search.cgi?searchType=miRNA&searchBox=hsa-miR-125b-3p&full=1	AD (*n* = 20), Ctrl (*n* = 20)	Serum<br>miRNA-seq/RT-qPCR	Expression of 72 miRNAs deregulated in AD (miR-146a-5p, miR-106b-3p, miR-195-5p, miR-20b-5p, and miR-497-5p upregulated, miR-125b-3p, miR-29c-3p, miR-93-5p and miR-19b-3p downregulated).	[182]
	↑		AD (*n* = 6), Ctrl (*n* = 6)	CNS (TCx)<br>Array/RT-qPCR	Expression of miRNA-9, miRNA-125b, miRNA-146a, miRNA-155 increased in AD CSF and brain tissue-derived extracellular fluid.	[169]
			AD (*n* = 27), Ctrl (*n* = 18)	CNS (TCx)<br>miRNA-seq/RT-qPCR	Expression of 20 miRNAs deregulated in AD, including miR-501-3p, let-7f-5p, and miR-26b-5p.	[167]

Epigenetic mechanism	Effect	Gene/Target pathway involved [1]	Study model [2]	Tissue Study/design	Main results	References
**miR-125b-3p**	↑	http://www.mirdb.org/cgi-bin/search.cgi?searchType=miRNA&searchBox=hsa-miR-125b-3p&full=1	AD (*n* = 5), Ctrl (*n* = 5)	CNS (TCx) RT-qPCR	Expression of miR-146a, miR-155, miR-9, miR-125b, miR-34a, miR-25, miR-128, miR-15b, miR-454, miR-642, miR-20a, and miR-106a increased in AD neocortical extracellular fluid and CSF samples.	[171]
			AD (*n* = 26), Ctrl (*n* = 19)	CNS (TCx) Array	Expression of miR-7, miR-9-1, miR-23a/miR-27a, miR-34a, miR-125b-1, miR-146a, and miR-155 increased in AD-affected superior TCx.	[189]
			AD (*n* = 6), Ctrl (*n* = 6)	CNS (TCx) Array	Expression of miRNA-9, miRNA-125b and miRNA-146a upregulated in AD TCx.	[170]

Epigenetic mechanism	Effect	Gene/Target pathway involved [1]	Study model [2]	Tissue Study/design	Main results	References
**miR-125b-3p**	↑	http://www.mirdb.org/cgi-bin/search.cgi?searchType=miRNA&searchBox=hsa-miR-125b-3p&full=1	Braak III–VI (*n* = 20), Braak 0–I (*n* = 7)	CNS (HPC, MFG, CB) RT-qPCR	Expression of miR-145, miR-148a, miR-200c, miR-210, miR-26a, miR-30c, miR-30e-5p, miR-423, and miR-92 deregulated in HPC and/or MFG. Expression of miR-100, miR-125b, miR-132, miR-146b, miR-212, miR-27a, miR-27b, miR-34a, miR-381, miR-422a, miR-425, miR-9 deregulated in CB. Increased expression of miR-29a and miR-29b..	[165]
			AD (*n* = 3), Ctrl (*n* = 3)	CNS (HPC) Array	Expression of miR-34a, miR-125b, miR-146a, miR-155 upregulated in AD brain samples	[191]
			AD (*n* = 10), Ctrl (*n* = 5)	CNS (FCx) RT-qPCR	Expression of miR-125b increased and of miR-29a and miR-29b decreased in AD.	[158]
			AD (*n* = 9), MCI (*n* = 8), Ctrl (*n* = 10)	CNS RT-qPCR	Expression of miR-125b upregulated in AD.	[159]

Epigenetic mechanism	Effect	Gene/Target pathway involved [1]	Study model [2]	Tissue Study/design	Main results	References
	↓		AD (*n* = 69), PD (*n* = 67), Ctrl (*n* = 78)	CSF miRNA-seq	Expression of 41 miRNAs deregulated in AD CSF, and 20 miRNAs in AD serum.	[164]
**miR-127-3p**		http://www.mirdb.org/cgi-bin/search.cgi?searchType=miRNA&searchBox=hsa-miR-127-3p&full=1	AD (*n* = 19/19), Ctrl (*n* = 19/19)	CSF Array/RT-qPCR	Expression of miRNA-139-5p, miRNA-181-5p, miRNA-127-3p, miRNA-140-3p, miRNA-374b, miRNA-376c, miRNA-532-3p, miRNA-758, miRNA-744, miRNA-642 downregulated, miRNA-129-3p, miRNA-206 upregulated in EOAD. Let-7a, miRNA-210, miRNA-224 downregulated and miRNA-223-5p, miRNA-486-5p, miRNA-424-3p and miRNA-625-3p upregulated in LOAD.	[218]
	↑		AD (*n* = 210), Probable FTD (*n* = 54), Ctrl (*n* = 53)	Plasma RT-qPCR	Expression of miR-127-3p downregulated in FTD compared to controls and AD.	[300]

Epigenetic mechanism	Effect	Gene/Target pathway involved [1]	Study model [2]	Tissue Study/design	Main results	References
**miR-128b**	↑	http://www.mirdb.org/cgi-bin/search.cgi?searchType=miRNA&searchBox=hsa-miR-155-5p&full=1	AD (*n* = 12), Ctrl (*n* = 6)	CNS (FCx, TCx) RT-qPCR	Expression of miR-128a/b reduced in AD TCx and of miR-128b in AD FCx.	[301]
**miR-132(-3p)**	↓	http://www.mirdb.org/cgi-bin/search.cgi?searchType=miRNA&searchBox=hsa-miR-132-3p&full=1	AD (*n* = 31), AD-MCI (*n* = 16), Ctrl (*n* = 16)	Plasma (Exosomes) Array/RT-qPCR	Expression of miR-132-3p and miR-212 was decreased in neurally-derived plasma EVs from AD patients.	[201]
			AD (*n* = 11), Ctrl (*n* = 8)	CNS Array/RT-qPCR	Expression of miR-132-3p and miR-212 was decreased in neurally-derived plasma EVs from AD patients.	[201]
			AD (*n* = 6), PD (*n* = 6), Ctrl (*n* = 6)	CNS (HPC, TCx, FCx) miRNA-seq/RT-qPCR	Expression of miR-184, miR-34c-3p, miR-375 and miR-132-5p, and of the other members of the 132/212 cluster downregulated in AD brain.	[202]

Epigenetic mechanism	Effect	Gene/Target pathway involved [1]	Study model [2]	Tissue Study/design	Main results	References
**miR-132(-3p)**	↓	http://www.mirdb.org/cgi-bin/search.cgi?searchType=miRNA&searchBox=hsa-miR-132-3p&full=1	Braak III–VI (*n* = 20), Braak 0–I (*n* = 7)	CNS (HPC, MFG, CB)<br>RT-qPCR	Expression of miR-145, miR-148a, miR-200c, miR-210, miR-26a, miR-30c, miR-30e-5p, miR-423, and miR-92 deregulated in HPC and/or MFG. Expression of miR-100, miR-125b, miR-132, miR-146b, miR-212, miR-27a, miR-27b, miR-34a, miR-381, miR-422a, miR-425, miR-9 deregulated in CB. Increased expression of miR-29a and miR-29b..	[165]
			AD (*n* = 27), Ctrl (*n* = 18)	CNS (TCx)<br>miRNA-seq/RT-qPCR	Expression of 20 miRNAs deregulated in AD, including miR-501-3p, let-7f-5p, and miR-26b-5p.	[167]
			AD (*n* = 5), DLB (*n* = 4), FTD (*n* = 5), HS-aging (*n* = 4), Ctrl (*n* = 2)	CNS (TCx)<br>RNA deep seq/RT-qPCR	Expression of miR-132 altered in AD and FTD cases.	[203]
			HPC: AD (*n* = 41), Ctrl (*n* = 23); FCx: AD (*n* = 21), Ctrl (*n* = 28), TCx: AD (*n* = 8), Ctrl (*n* = 8)	CNS (HPC, FCx, TCx)<br>RNA deep seq/RT-qPCR	Expression of 41 miRNAs deregulated in AD HPC.	[204]

Epigenetic mechanism	Effect	Gene/Target pathway involved [1]	Study model [2]	Tissue Study/design	Main results	References
	↓		TCx: AD (*n* = 39/8), Ctrl (*n* = 25/8); FCx: AD (*n* = 225/8), Ctrl (*n* = 87/8)	CNS (FCx, TCx) Array/RT-qPCR	Expression of 102 miRNAs deregulated in AD temporal cortical brain regions, including downregulated expression of miR132-3p and miR-212-3p in AD FCx and TCx.	[162]
			FCx: AD (*n* = 7), Ctrl (*n* = 5); TCx: AD (*n* = 8/11), MCI (*n* = 0/10), Ctrl (*n* = 8/11); HPC: AD (*n* = 10), Ctrl (*n* = 13)	CNS (HPC, TCx, FCx) RT-qPCR	Expression of miR-132 is downregulated in AD.	[205]
**miR-132(-3p)**		http://www.mirdb.org/cgi-bin/search.cgi?searchType=miRNA&searchBox=hsa-miR-132-3p&full=1	AD (*n* = 3/10), MCI (*n* = 0/10), Ctrl (*n* = 3/12)	CNS (FCx) Array/RT-qPCR	Expression of miR-498, miR-150, miR-886-3p, miR-132, miR-21, miR-23a, miR-140-3p, miR-212, miR-23b, miR-181a and let-7d upregulated in AD FCx, and of miR-212, miR-132 and miR-150 upregulated in AD TCx.	[206]
			Braak IV (*n* = 18), Braak III/IV (*n* = 14), Ctrl (*n* = 18)	CNS (TCx) RT-qPCR	Expression of miR-132 and miR-212 downregulated in AD TCX and CA1 HPC neurons.	[207]

Epigenetic mechanism	Effect	Gene/Target pathway involved [1]	Study model [2]	Tissue Study/design	Main results	References
**miR-132(-3p)**	↑	http://www.mirdb.org/cgi-bin/search.cgi?searchType=miRNA&searchBox=hsa-miR-132-3p&full=1	MCI (*n* = 66), Ctrl (*n* = 76)	Serum<br>RT-qPCR	Expression of miR-206 and miR-132 elevated in MCI patients.	[208]
**miR-133b**	↓	http://www.mirdb.org/cgi-bin/search.cgi?searchType=miRNA&searchBox=hsa-miR-133b&full=1	AD (*n* = 105), Ctrl (*n* = 98)	Serum<br>RT-qPCR	Expression of miR-133b downregulated in AD.	[302]
**miR-135a**	↓	http://www.mirdb.org/cgi-bin/search.cgi?searchType=miRNA&searchBox=hsa-miR-135a-5p&full=1	AD (*n* = 5), Ctrl (*n* = 5)	CSF<br>RT-qPCR	Expression of miR-135a and miR-200b downregulated in AD.	[303]
			AD (*n* = 107), MCI (*n* = 101), PD (*n* = 30), VD (*n* = 20), Ctrl (*n* = nR)	Serum (Exosomes)<br>RT-qPCR	Expression of miR-135a and miR-384 upregulated, and of miR-193b downregulated in AD.	[304]

Epigenetic mechanism	Effect	Gene/Target pathway involved [1]	Study model [2]	Tissue Study/design	Main results	References
**miR-135a**	↓	http://www.mirdb.org/cgi-bin/search.cgi?searchType=miRNA&searchBox=hsa-miR-135a-5p&full=1	Braak V (*n* = 10), Braak 0 (*n* = 10)	CSF<br>RT-qPCR	Expression of miR-145, miR-148a, miR-200c, miR-210, miR-26a, miR-30c, miR-30e-5p, miR-423, and miR-92 deregulated in HPC and/or MFG. Expression of miR-100, miR-125b, miR-132, miR-146b, miR-212, miR-27a, miR-27b, miR-34a, miR-381, miR-422a, miR-425, miR-9 deregulated in CB. Increased expression of miR-29a and miR-29b.	[165]
**miR-135b**	↓	http://www.mirdb.org/cgi-bin/search.cgi?searchType=miRNA&searchBox=hsa-miR-135b-5p&full=1	AD (*n* = 25), Ctrl (*n* = 25)	Blood<br>RT-qPCR	Expression of miR-135b reduced AD patients.	[155]
**miR-136-3p**	↓	http://www.mirdb.org/cgi-bin/search.cgi?searchType=miRNA&searchBox=hsa-miR-136-3p&full=1	AD (*n* = 69), PD (*n* = 67), Ctrl (*n* = 78)	CSF<br>miRNA-seq	Expression of 41 miRNAs deregulated in AD CSF, and 20 miRNAs in AD serum.	[164]
			AD (*n* = 28), PD (*n* = 47), Ctrl (*n* = 27)	CSF (Exosomes)<br>Array/RT-qPCR	Expression of miR-16-2, miR-331-5p, miR-132-5p, miR-485-5p, miR-151, miR-136-3p, and miR-29c deregulated in AD CSF.	[183]

Epigenetic mechanism	Effect	Gene/Target pathway involved [1]	Study model [2]	Tissue Study/design	Main results	References
	↓		AD (*n* = 69), PD (*n* = 67), Ctrl (*n* = 78)	CSF miRNA-seq	Expression of 41 miRNAs deregulated in AD CSF, and 20 miRNAs in AD serum.	[164]
**miR-139-5p**		http://www.mirdb.org/cgi-bin/search.cgi?searchType=miRNA&searchBox=hsa-miR-139-5p&full=1	AD (*n* = 19/19), Ctrl (*n* = 19/19)	CSF Array/RT-qPCR	Expression of miR-139-5p, miR-181-5p, miR-127-3p, miR-140-3p, miR-374b, miR-376c, miR-532-3p, miR-758, miR-744, and miR-642 downregulated miR-129-3p, and miR-206 upregulated in EOAD. Let-7a, miR-210 and miR-224 downregulated, and miR-223-5p, miR-486-5p, miR-424-3p and miR-625-3p upregulated in LOAD.	[218]

Epigenetic mechanism	Effect	Gene/Target pathway involved [1]	Study model [2]	Tissue Study/design	Main results	References
**miR-141**	↑	http://www.mirdb.org/cgi-bin/search.cgi?searchType=miRNA&searchBox=hsa-miR-141-3p&full=1	Braak V (*n* = 10), Braak 0 (*n* = 10)	CSF<br>RT-qPCR	Expression of miR-145, miR-148a, miR-200c, miR-210, miR-26a, miR-30c, miR-30e-5p, miR-423, and miR-92 deregulated in HPC and/or MFG. Expression of miR-100, miR-125b, miR-132, miR-146b, miR-212, miR-27a, miR-27b, miR-34a, miR-381, miR-422a, miR-425, miR-9 deregulated in CB. Increased expression of miR-29a and miR-29b.	[165]
			AD (*n* = 10/18), Ctrl (*n* = 10/18)	CSF<br>RT-qPRC	Expression of 15 miRNAs deregulated in AD, miR-9-5p and miR-598 suggested as potential biomarkers for AD.	[168]
**miR-142-3p**	↓	http://www.mirdb.org/cgi-bin/search.cgi?searchType=miRNA&searchBox=hsa-miR-142-3p&full=1	AD (*n* = 21/15), preclinical AD (*n* = 21/15), Ctrl (*n* = 21/15), PD (*n* = 21/0)	Plasma<br>RT-qPCR	Expression levels of miR-15b-5p, miR-34a-5p, miR-142-3p, and miR-545-3p distinguished AD from PD and HC subjects.	[187]
			AD (*n* = 11/20), MCI (*n* = 9/0), Ctrl (*n* = 20/17)	Plasma miRNA-seq/RT-qPCR	Expression of let-7d-5p, let-7g-5p, miR-15b-5p, miR-142-3p, miR-191-5p, miR-301a-3p and miR-545-3p deregulated in AD.	[291]

Epigenetic mechanism	Effect	Gene/Target pathway involved [1]	Study model [2]	Tissue Study/design	Main results	References
**miR-142-3p**	↓	http://www.mirdb.org/cgi-bin/search.cgi?searchType=miRNA&searchBox=hsa-miR-142-3p&full=1	AD (*n* = 7/13), MCI-AD (*n* = 7/8), Ctrl (*n* = 6/9)	Plasma<br>RT-qPCR	Expression of miR-151a-5p, miR-30b-5p, miR-486-5p, miR-33a-5p, miR-483-5p, miR-18a-5p, miR-320a, miR-320b, miR-320c, miR-502-3p, miR-103a-3p, miR-301a-3p, miR-142-3p, and miR-200a-3p deregulated in AD.	[298]
**miR-146a(-5p)**	↓	http://www.mirdb.org/cgi-bin/search.cgi?searchType=miRNA&searchBox=hsa-miR-146a-5p&full=1	AD (*n* = 127), MCI (*n* = 30), VD (*n* = 30)	Serum<br>miRNA-seq/RT-qPCR	Expression of miR-31, miR-93, miR-143, and miR-146a decreased in AD serum, and of miR-93, and miR-146a increased in MCI.<br>The panel of miR-31, miR-93 and miR-146a can be used to discriminate AD from VD.	[209]
			AD (*n* = 10), Ctrl (*n* = 10)	Plasma<br>RT-qPCR	Expression of miR-34a and miR-146a decreased in AD plasma, and of miR-34a, miR-125b, and miR-146a in CSF.	[173]
			AD (*n* = 40), Ctrl (*n* = 31); Validation cohort: publicly available dataset of miRnA data	Blood<br>miRNA-seq	Expression of 71 deregulated in AD, including miR-146b-5p and miR-15b-5p.	[210]

Epigenetic mechanism	Effect	Gene/Target pathway involved [1]	Study model [2]	Tissue Study/design	Main results	References
	↓		AD (*n* = 10), Ctrl (*n* = 10)	CSF RT-qPCR	Expression of miR-34a and miR-146a decreased in AD plasma, and of miR-34a, miR-125b, and miR-146a in CSF.	[173]
			AD (*n* = 50/16), Ctrl (*n* = 49/16)	CSF Array/RT-qPCR	Expression of 36 miRNAs (20 in validation cohort) discriminate AD from control CSF.	[174]
**miR-146a(-5p)**		http://www.mirdb.org/cgi-bin/search.cgi?searchType=miRNA&searchBox=hsa-miR-146a-5p&full=1	AD (*n* = 20), Ctrl (*n* = 20)	CSF RT-qPCR	Expression of miR-16, miR-34c, miR-107, miR-128a, and miR-146a were differentially regulated in AD HPC.	[196]
			AD (*n* = 60), MCI-AD (*n* = 39), FTD (*n* = 37), DLB (*n* = 37), Ctrl (*n* = 40)	CSF RT-qPCR	Expression of miR-29a increased in AD CSF.	[175]

Epigenetic mechanism	Effect	Gene/Target pathway involved [1]	Study model [2]	Tissue Study/design	Main results	References
	↓		Braak III–VI (*n* = 20), Braak 0–I (*n* = 7)	CNS (HPC, MFG, CB) RT-qPCR	Expression of miR-145, miR-148a, miR-200c, miR-210, miR-26a, miR-30c, miR-30e-5p, miR-423, and miR-92 deregulated in HPC and/or MFG. Expression of miR-100, miR-125b, miR-132, miR-146b, miR-212, miR-27a, miR-27b, miR-34a, miR-381, miR-422a, miR-425, miR-9 deregulated in CB. Increased expression of miR-29a and miR-29b.	[165]
**miR-146a(-5p)**		http://www.mirdb.org/cgi-bin/search.cgi?searchType=miRNA&searchBox=hsa-miR-146a-5p&full=1	AD (*n* = 27), Ctrl (*n* = 18)	CNS (TCx) miRNA-seq/RT-qPCR	Expression of 20 miRNAs deregulated in AD, including miR-501-3p, let-7f-5p, and miR-26b-5p.	[167]
			AD (*n* = 10), Ctrl (*n* = 11)	CNS (HPC) Array	Expression of miR-16, miR-34c, miR-107, miR-128a, and miR-146a were differentially regulated in AD HPC.	[196]

Epigenetic mechanism	Effect	Gene/Target pathway involved [1]	Study model [2]	Tissue Study/design	Main results	References
	↓		Braak III–VI (*n* = 20), Braak 0–I (*n* = 7)	CNS (HPC, MFG, CB) RT-qPCR	Expression of miR-145, miR-148a, miR-200c, miR-210, miR-26a, miR-30c, miR-30e-5p, miR-423, and miR-92 deregulated in HPC and/or MFG. Expression of miR-100, miR-125b, miR-132, miR-146b, miR-212, miR-27a, miR-27b, miR-34a, miR-381, miR-422a, miR-425, miR-9 deregulated in CB. Increased expression of miR-29a and miR-29b.	[165]
**miR-146a(-5p)**		http://www.mirdb.org/cgi-bin/search.cgi?searchType=miRNA&searchBox=hsa-miR-146a-5p&full=1	AD (*n* = 27), Ctrl (*n* = 18)	CNS (TCx) miRNA-seq/RT-qPCR	Expression of 20 miRNAs deregulated in AD, including miR-501-3p, let-7f-5p, and miR-26b-5p.	[167]
	↑		AD (*n* = 20), Ctrl (*n* = 20)	Serum miRNA-seq/RT-qPCR	Expression of 72 miRNAs deregulated in AD (miR-146a-5p, miR-106b-3p, miR-195-5p, miR-20b-5p, and miR-497-5p upregulated, miR-125b-3p, miR-29c-3p, miR-93-5p and miR-19b-3p downregulated).	[182]

Epigenetic mechanism	Effect	Gene/Target pathway involved [1]	Study model [2]	Tissue Study/design	Main results	References
	↑		AD (*n* = 6), Ctrl (*n* = 6)	CSF Array/RT-qPCR	Expression of miRNA-9, miRNA-125b, miRNA-146a, miRNA-155 increased in AD CSF and brain tissue-derived extracellular fluid.	[169]
			AD (*n* = 22), Ctrl (*n* = 28)	CSF RT-qPCR	Expression of 74 down- and 74 up-regulated mi-RNAs detected in AD, including miR-100, miR-146a and miR-1274a.	[211]
**miR-146a(-5p)**		http://www.mirdb.org/cgi-bin/search.cgi?searchType=miRNA&searchBox=hsa-miR-146a-5p&full=1	AD (*n* = 6), Ctrl (*n* = 6)	CNS (TCx) Array/RT-qPCR	Expression of miRNA-9, miRNA-125b, miRNA-146a, miRNA-155 increased in AD CSF and brain tissue-derived extracellular fluid.	[169]
			AD (*n* = 36), Ctrl (*n* = 30)	CNS (HPC, TCx) Array	Expression of miRNA-146a upregulated in AD superior TCx.	[212]
			AD (*n* = 5), Ctrl (*n* = 5)	CNS (TCx) RT-qPCR	Expression of miR-146a, miR-155, miR-9, miR-125b, miR-34a, miR-25, miR-128, miR-15b, miR-454, miR-642, miR-20a, and miR-106a increased in AD neocortical extracellular fluid and CSF samples.	[171]

Epigenetic mechanism	Effect	Gene/Target pathway involved [1]	Study model [2]	Tissue Study/design	Main results	References
**miR-146a(-5p)**	↑	http://www.mirdb.org/cgi-bin/search.cgi?searchType=miRNA&searchBox=hsa-miR-146a-5p&full=1	AD (*n* = 26), Ctrl (*n* = 19)	CNS (TCx) Array	Expression of miR-7, miR-9-1, miR-23a/miR-27a, miR-34a, miR-125b-1, miR-146a, and miR-155 increased in AD-affected superior TCx.	[189]
			AD (*n* = 6), Ctrl (*n* = 6)	CNS (TCx) Array	Expression of miRNA-9, miRNA-125b and miRNA-146a upregulated in AD TCx.	[170]
			AD (*n* = 12), Ctrl (*n* = 6)	CNS Array	Expression of miR-7, miR-146a, and miR-155 increased in AD.	[213]
			AD (*n* = 23), Ctrl (*n* = 23)	CNS (TCx) Array	Expression of miR-146a coupled to down-regulation of CFH observed in AD brain.	[214]
			AD (*n* = 3), Ctrl (*n* = 3)	CNS (HPC) Array	Expression of miR-34a, miR-125b, miR-146a, miR-155 upregulated in AD brain samples.	[191]
**miR-151a-3p**	↑	http://www.mirdb.org/cgi-bin/search.cgi?searchType=miRNA&searchBox=hsa-miR-151a-3p&full=1	AD (*n* = 48/106), MCI (*n* = 18/0), MS (*n* = 16/0), PD (*n* = 9/0), DEP (*n* = 15/0), BD (*n* = 15/0), SCH (*n* = 14/0), Ctrl (*n* = 22/22)	Blood miRNA-seq/RT-qPCR	Expression of 82 miRNAs upregulated and 58 downregulated in AD patient samples.	[192]

Epigenetic mechanism	Effect	Gene/Target pathway involved [1]	Study model [2]	Tissue Study/design	Main results	References
**miR-151a-3p**	↑	http://www.mirdb.org/cgi-bin/search.cgi?searchType=miRNA&searchBox=hsa-miR-151a-3p&full=1	AD (*n* = 48), Ctrl (*n* = 22)	BloodomiRas and DIANA miRPath	Expression of 27 miRNAs discriminate between two groups.	[178]
**miR-155**	↑	http://www.mirdb.org/cgi-bin/search.cgi?searchType=miRNA&searchBox=hsa-miR-155-5p&full=1	AD (*n* = 36), MCI (*n* = 52), Ctrl (*n* = 6)	PBMC<br>RT-qPCR	Expression of immune-related miRNAs, including miR-155, miR-154, miR-200b, miR-27b, and miR-128, differentially expressed in PBMCs from AD and MCI subjects.	[215]
			AD (*n* = 16), Ctrl (*n* = 16)	PBMC<br>Array/RT-qPCR	Expression of 22 miRNAs upregulated in AD.	[188]
			AD (*n* = 6), Ctrl (*n* = 6)	CNS (TCx)<br>Array/RT-qPCR	Expression of miR-9, miR-125b, miR-146a, miR-155 increased in AD CSF and brain tissue-derived extracellular fluid.	[169]
			AD (*n* = 12), Ctrl (*n* = 6)	CNS<br>Array	Expression of miR-7, miR-146a, and miR-155 increased in AD.	[213]

Epigenetic mechanism	Effect	Gene/Target pathway involved [1]	Study model [2]	Tissue Study/design	Main results	References
	↑		AD (*n* = 5), Ctrl (*n* = 5)	CNS (TCx) RT-qPCR	Expression of miR-146a, miR-155, miR-9, miR-125b, miR-34a, miR-25, miR-128, miR-15b, miR-454, miR-642, miR-20a, and miR-106a increased in AD neocortical extracellular fluid and CSF samples.	[171]
			AD (*n* = 3), Ctrl (*n* = 3)	CNS (HPC) Array	Expression of miR-34a, miR-125b, miR-146a, miR-155 upregulated in AD brain samples.	[191]
**miR-155**		http://www.mirdb.org/cgi-bin/search.cgi?searchType=miRNA&searchBox=hsa-miR-155-5p&full=1	Braak III–VI (*n* = 20), Braak 0–I (*n* = 7)	CNS (HPC, MFG, CB) RT-qPCR	Expression of miR-145, miR-148a, miR-200c, miR-210, miR-26a, miR-30c, miR-30e-5p, miR-423, and miR-92 deregulated in HPC and/or MFG. Expression of miR-100, miR-125b, miR-132, miR-146b, miR-212, miR-27a, miR-27b, miR-34a, miR-381, miR-422a, miR-425, miR-9 deregulated in CB. Increased expression of miR-29a and miR-29b.	[165]

Epigenetic mechanism	Effect	Gene/Target pathway involved [1]	Study model [2]	Tissue Study/design	Main results	References
**miR-155**	↑	http://www.mirdb.org/cgi-bin/search.cgi?searchType=miRNA&searchBox=hsa-miR-155-5p&full=1	AD (*n* = 27), Ctrl (*n* = 18)	CNS (TCx) miRNA-seq/RT-qPCR	Expression of 20 miRNAs deregulated in AD, including miR-501-3p, let-7f-5p, and miR-26b-5p.	[167]
			AD (*n* = 26), Ctrl (*n* = 19)	CNS (TCx) Array	Expression of miR-7, miR-9-1, miR-23a/miR-27a, miR-34a, miR-125b-1, miR-146a, and miR-155 increased in AD-affected superior TCx.	[189]
**miR-181a-5p**	↓	http://www.mirdb.org/cgi-bin/search.cgi?searchType=miRNA&searchBox=hsa-miR-181a-5p&full=1	Braak III–VI (*n* = 20), Braak 0–I (*n* = 7)	CNS (HPC, MFG, CB) RT-qPCR	Expression of miR-145, miR-148a, miR-200c, miR-210, miR-26a, miR-30c, miR-30e-5p, miR-423, and miR-92 deregulated in HPC and/or MFG. Expression of miR-100, miR-125b, miR-132, miR-146b, miR-212, miR-27a, miR-27b, miR-34a, miR-381, miR-422a, miR-425, miR-9 deregulated in CB. Increased expression of miR-29a and miR-29b.	[165]

Epigenetic mechanism	Effect	Gene/Target pathway involved [1]	Study model [2]	Tissue Study/design	Main results	References
**miR-181a-5p**	↓	http://www.mirdb.org/cgi-bin/search.cgi?searchType=miRNA&searchBox=hsa-miR-181a-5p&full=1	AD (*n* = 3/10), MCI (*n* = 0/10), Ctrl (*n* = 3/12)	CNS (FCx) Array/RT-qPCR	Expression of miR-498, miR-150, miR-886-3p, miR-132, miR-21, miR-23a, miR-140-3p, miR-212, miR-23b, miR-181a and let-7d upregulated in AD FCx, and of miR-212, miR-132 and miR-150 upregulated in AD TCx.	[206]
**miR-181c(-5p)**	↓	http://www.mirdb.org/cgi-bin/search.cgi?searchType=miRNA&searchBox=hsa-miR-181c-5p&full=1	Probable AD (*n* = 7), aMCI/Probable Early AD (*n* = 7), Ctrl (*n* = 7)	Serum RT-qPCR	Expression of miR-137, miR-181c, miR-9, miR-29a/b downregulated in serum of probable AD patients.	[176]
			Probable AD (*n* = 105), Ctrl (*n* = 150)	Serum RT-qPCR	Expression of miR-125b and miR-181c downregulated, and of miR-9 upregulated in serum of AD patients.	[198]
			AD (*n* = 7), Ctrl (*n* = 7)	CNS (Neocortex) RT-qPCR	Expression of miR-137, miR-181c, miR-9, miR-29a, and miR-29b-1, miR-15 and miR-124 downregulated in the FCx of the subgroup of sporadic AD patient.	[166]
			AD (*n* = 5), Ctrl (*n* = 5)	CNS (PCx) Array	Expression of 48 miRNAs deregulated in AD brain.	[180]

Epigenetic mechanism	Effect	Gene/Target pathway involved [1]	Study model [2]	Tissue Study/design	Main results	References
**miR-181c(-5p)**	↑	http://www.mirdb.org/cgi-bin/search.cgi?searchType=miRNA&searchBox=hsa-miR-181c-5p&full=1	AD (*n* = 56/0), MCI (*n* = 26/0), FTD (*n* = 0/27), Ctrl (*n* = 14/24)	Plasma<br>RT-qPCR	Expression of miR-92a-3p, miR-181c-5p and miR-210-3p increased in AD and MCI subjects.	[216]
**miR-181d-5p**	↓	http://www.mirdb.org/cgi-bin/search.cgi?searchType=miRNA&searchBox=hsa-miR-181d-5p&full=1	AD (*n* = 27), Ctrl (*n* = 18)	CNS (TCx)<br>miRNA-seq/RT-qPCR	Expression of 20 miRNAs deregulated in AD, including miR-501-3p, let-7f-5p, and miR-26b-5p.	[167]
**miR-191-5p**	↓	http://www.mirdb.org/cgi-bin/search.cgi?searchType=miRNA&searchBox=hsa-miR-191-5p&full=1	AD (*n* = 11/20), MCI (*n* = 0/9), Ctrl (*n* = 20/17)	Plasma<br>miRNA-seq/RT-qPCR	Expression of let-7d-5p, let-7g-5p, miR-15b-5p, miR-142-3p, miR-191-5p, miR-301a-3p and miR-545-3p deregulated in AD.	[291]
			AD (*n* = 50/158), Ctrl (*n* = 50/155)	Serum<br>miRNA-seq/RT-qPCR	Expression of miR-98-5p, miR-885-5p, miR-483-3p, miR-342-3p, miR-191-5p, and miR-let-7d-5p deregulated in AD patients.	[198]
**miR-193a-3p**	↓	http://www.mirdb.org/cgi-bin/search.cgi?searchType=miRNA&searchBox=hsa-miR-193a-3p&full=1	AD (*n* = 108), Ctrl (*n* = 93)	Serum<br>RT-qPCR	Expression of miR-193a-3p downregulated in AD.	[305]

Epigenetic mechanism	Effect	Gene/Target pathway involved [1]	Study model [2]	Tissue Study/design	Main results	References
**miR-193b**	↓	http://www.mirdb.org/cgi-bin/search.cgi?searchType=miRNA&searchBox=hsa-miR-193b-3p&full=1	AD (*n* = 107), MCI (*n* = 101), PD (*n* = 30), VD (*n* = 20), Ctrl (*n* = nR)	Serum (Exosomes) RT-qPCR	Expression of miR-135a and miR-384 upregulated, and of miR-193b downregulated in AD.	[304]
**miR-200a-3p**	↓	http://www.mirdb.org/cgi-bin/search.cgi?searchType=miRNA&searchBox=hsa-miR-200a-3p&full=1	AD (*n* = 7), Ctrl (*n* = 5)	Plasma RT-qPCR	Expression of miR-200a-3p levels downregulated in AD blood plasma.	[306]
	↑		AD (*n* = 7/13), MCI-AD (*n* = 7/8), Ctrl (*n* = 6/9)	Plasma RT-qPCR	Expression of miR-151a-5p, miR-30b-5p, miR-486-5p, miR-33a-5p, miR-483-5p, miR-18a-5p, miR-320a, miR-320b, miR-320c, miR-502-3p, miR-103a-3p, miR-301a-3p, miR-142-3p, and miR-200a-3p deregulated in AD.	[298]
**miR-206**	↑	http://www.mirdb.org/cgi-bin/search.cgi?searchType=miRNA&searchBox=hsa-miR-206&full=1	AD (*n* = 25), MCI (*n* = 30), Ctrl (*n* = 31); Longitudinal cohort: MCI-MCI-Dementia (*n* = 6), Ctrl-MCI-Dementia (*n* = 6), Ctrl-MCI-MCI (*n* = 6)	Plasma Array/RT-qPCR	Expression of let-7b increased in MCI subjects. Expression of miR-206 increased in AD subjects.	[217]
			MCI (*n* = 66), Ctrl (*n* = 76)	Serum RT-qPCR	Expression of miR-206 and miR-132 elevated in MCI patients.	[208]

Epigenetic mechanism	Effect	Gene/Target pathway involved [1]	Study model [2]	Tissue Study/design	Main results	References
**miR-206**	↑		AD (*n* = 10/18), Ctrl (*n* = 10/18)	CSF RT-qPRC	Expression of 15 miRNAs deregulated in AD. miR-9-5p and miR-598 suggested as potential biomarkers for AD.	[168]
		http://www.mirdb.org/cgi-bin/search.cgi?searchType=miRNA&searchBox=hsa-miR-206&full=1	AD (*n* = 19/19); Ctrl (*n* = 19/19)	CSF Array/RT-qPCR	Expression of miR-139-5p, miR-181-5p, miR-127-3p, miR-140-3p, miR-374b, miR-376c, miR-532-3p, miR-758, miR-744, and miR-642 downregulated miR-129-3p, and miR-206 upregulated in EOAD. Let-7a, miR-210 and miR-224 downregulated, and miR-223-5p, miR-486-5p, miR-424-3p and miR-625-3p upregulated in LOAD.	[218]
**miR-210-3p**	↑	http://www.mirdb.org/cgi-bin/search.cgi?searchType=miRNA&searchBox=hsa-miR-210-3p&full=1	AD (*n* = 56/0), MCI (*n* = 26/0), FTD (*n* = 0/27), Ctrl (*n* = 14/24)	Plasma RT-qPCR	Expression of miR-92a-3p, miR-181c-5p and miR-210-3p increased in AD and MCI subjects.	[216]
**miR-212(-3p)**	↓	http://www.mirdb.org/cgi-bin/search.cgi?searchType=miRNA&searchBox=hsa-miR-212-3p&full=1	AD (*n* = 31), AD-MCI (*n* = 16), Ctrl (*n* = 16)	Plasma (Exosomes) Array/RT-qPCR	Expression of miR-132-3p and miR-212 decreased in neurally-derived plasma exosomes from AD patients.	[201]

Epigenetic mechanism	Effect	Gene/Target pathway involved [1]	Study model [2]	Tissue Study/design	Main results	References
	↓		AD (*n* = 11), Ctrl (*n* = 8)	CNS<br>Array/RT-qPCR	Expression of miR-132-3p and miR-212 decreased in neurally-derived plasma exosomes from AD patients.	[201]
			AD (*n* = 6), PD (*n* = 6), Ctrl (*n* = 6)	CNS (HPC, TCx, FCx)<br>miRNA-seq/RT-qPCR	Expression of miR-184, miR-34c-3p, miR-375, miR-132-5p, and other members of the 132/212 cluster downregulated in AD brain.	[202]
**miR-212(-3p)**		http://www.mirdb.org/cgi-bin/search.cgi?searchType=miRNA&searchBox=hsa-miR-212-3p&full=1	Braak III–VI (*n* = 20), Braak 0–I (*n* = 7)	CNS (HPC, MFG, CB)<br>RT-qPCR	Expression of miR-145, miR-148a, miR-200c, miR-210, miR-26a, miR-30c, miR-30e-5p, miR-423, and miR-92 deregulated in HPC and/or MFG. Expression of miR-100, miR-125b, miR-132, miR-146b, miR-212, miR-27a, miR-27b, miR-34a, miR-381, miR-422a, miR-425, miR-9 deregulated in CB. Increased expression of miR-29a and miR-29b.	[165]

Epigenetic mechanism	Effect	Gene/Target pathway involved [1]	Study model [2]	Tissue Study/design	Main results	References
	↓		AD (*n* = 3/10), MCI (*n* = 10), Ctrl (*n* = 3/12)	CNS (FCx) Array/RT-qPCR	Expression of miR-498, miR-150, miR-886-3p, miR-132, miR-21, miR-23a, miR-140-3p, miR-212, miR-23b, miR-181a and let-7d upregulated in AD FCx, and of miR-212, miR-132 and miR-150 upregulated in AD TCx.	[206]
**miR-212(-3p)**		http://www.mirdb.org/cgi-bin/search.cgi?searchType=miRNA&searchBox=hsa-miR-212-3p&full=1	Braak IV (*n* = 18), Braak III/IV (*n* = 14), Ctrl (*n* = 18)	CNS (TCx) RT-qPCR	Expression of miR-132 and miR-212 downregulated in AD TCx areas and CA1 HPC neurons.	[207]
			TCx: AD (*n* = 39/8), Ctrl (*n* = 25/8); FCx: AD (*n* = 225/8), Ctrl (*n* = 87/8)	CNS (FCx, TCx) Array/RT-qPCR	Expression of 102 miRNAs deregulated in AD temporal cortical brain regions, including downregulated expression of miR132-3p and miR-212-3p in AD FCx and TCx.	[162]
			AD (*n* = 27), Ctrl (*n* = 18)	CNS (TCx) miRNA-seq/RT-qPCR	Expression of 20 miRNAs deregulated in AD, including miR-501-3p, let-7f-5p, and miR-26b-5p.	[167]

Epigenetic mechanism	Effect	Gene/Target pathway involved [1]	Study model [2]	Tissue Study/design	Main results	References
**miR-222**	↓	http://www.mirdb.org/cgi-bin/search.cgi?searchType=miRNA&searchBox=hsa-miR-222-3p&full=1	Mild AD (*n* = 30), Moderate AD (*n* = 30), Ctrl (*n* = 30)	Serum<br>miRNA-seq/RT-qPCR	Expression of miR-222 decreased in the mild and moderate AD.	[307]
	↑		AD (*n* = 70), VD (*n* = 17), tauopathies other than AD (*n* = 23), Ctrl (*n* = 43)	CSF<br>RT-qPCR	Expression of miR-222 increased in VD patients compared to both control and AD subjects.	[308]
**miR-223**	↓	http://www.mirdb.org/cgi-bin/search.cgi?searchType=miRNA&searchBox=hsa-miR-223-3p&full=1	Probable AD (*n* = 84), Ctrl (*n* = 62)	Serum<br>RT-qPCR	Expression of miR-29, miR-125b and miR-223 decreased, and of miR-519 increased in AD patients.	[309]
			AD - no treatment (*n* = 11), AD - with treatment (*n* = 11), VD (*n* = 10), Ctrl (*n* = 16)	Serum (Exosomes)<br>RT-qPCR	Expression of miR-223 decreased in dementia patients.	[310]
**miR-301a-3p**	↓	http://www.mirdb.org/cgi-bin/search.cgi?searchType=miRNA&searchBox=hsa-miR-301a-3p&full=1	AD (*n* = 11/20), MCI (*n* = 9), Ctrl (*n* = 20/17)	Plasma<br>miRNA-seq/RT-qPCR	Expression of let-7d-5p, let-7g-5p, miR-15b-5p, miR-142-3p, miR-191-5p, miR-301a-3p and miR-545-3p deregulated in AD.	[291]

Epigenetic mechanism	Effect	Gene/Target pathway involved [1]	Study model [2]	Tissue Study/design	Main results	References
**miR-301a-3p**	↓	http://www.mirdb.org/cgi-bin/search.cgi?searchType=miRNA&searchBox=hsa-miR-301a-3p&full=1	AD (*n* = 7/13), MCI-AD (*n* = 7/8), Ctrl (*n* = 6/9)	Plasma<br>RT-qPCR	Expression of miR-151a-5p, miR-30b-5p, miR-486-5p, miR-33a-5p, miR-483-5p, miR-18a-5p, miR-320a, miR-320b, miR-320c, miR-502-3p, miR-103a-3p, miR-301a-3p, miR-142-3p, and miR-200a-3p deregulated in AD.	[298]
**miR-342-3p**	↓	http://www.mirdb.org/cgi-bin/search.cgi?searchType=miRNA&searchBox=hsa-miR-34a-3p&full=1	AD (*n* = 23/16), MCI (*n* = 3/8), Ctrl (*n* = 23/36)<br>AD (*n* = 35), Ctrl (*n* = 35)	Serum (Exosomes)<br>miRNA-seq/RT-qPCR<br>Plasma (Exosomes)<br>miRNA-seq	Expression of 17 miRNA deregulated (14 miRNAs upregulated and 3 downregulated) in AD.<br>Expression of 20 miRNAs deregulated in AD group.	[293]<br>[177]
**miR-384**	↑	http://www.mirdb.org/cgi-bin/search.cgi?searchType=miRNA&searchBox=hsa-miR-384&full=1	AD (*n* = 107), MCI (*n* = 101), PD (*n* = 30), VD (*n* = 20), Ctrl (*n* = nR)	Serum (Exosomes)<br>RT-qPCR	Expression of miR-135a and miR-384 upregulated, and of miR-193b downregulated in AD.	[304]
**miR-425**	↑	http://www.mirdb.org/cgi-bin/search.cgi?searchType=miRNA&searchBox=hsa-miR-425-5p&full=1	AD (*n* = 4 severe+4 mild/45), Ctrl (*n* = 4/41)<br>AD (*n* = 48), Ctrl (*n* = 22)	PBMC<br>Array/RT-qPCR<br>BloodomiRas and DIANA miRPath	Expression of miR-339 and miR-425 altered in AD.<br>Expression of 27 miRNAs discriminate between two groups.	[311]<br>[178]

Epigenetic mechanism	Effect	Gene/Target pathway involved [1]	Study model [2]	Tissue Study/design	Main results	References
**miR-451a**	↓	http://www.mirdb.org/cgi-bin/search.cgi?searchType=miRNA&searchBox=hsa-miR-451a&full=1	YOAD (*n* = 17/17), LOAD (*n* = 13/13), Ctrl (*n* = 12/12)	CSF (Exosomes)<br>RT-qPCR	Expression of miR-16-5p, miR-125b-5p, miR-451a, and miR-605-5p deregulated in AD CSF-derived exosomes.	[200]
**miR-455-3p**	↑	http://www.mirdb.org/cgi-bin/search.cgi?searchType=miRNA&searchBox=hsa-miR-455-3p&full=1	AD (*n* = 10/11), MCI (*n* = 16/20), Ctrl (*n* = 14/18)	Serum<br>Array/RT-qPCR	Expression of miR-455-3p, miR-4668-5p, miR-3613-3p, miR-4674 upregulated, and of mir-6722 downregulated in AD and MCI.	[312]
**miR-501-3p**	↓	http://www.mirdb.org/cgi-bin/search.cgi?searchType=miRNA&searchBox=hsa-miR-501-3p&full=1	AD (*n* = 27), Ctrl (*n* = 18)	Serum<br>miRNA-seq/RT-qPCR	Expression of 20 miRNAs deregulated in AD, including miR-501-3p, let-7f-5p, and miR-26b-5p.	[167]
**miR-545-3p**	↓	http://www.mirdb.org/cgi-bin/search.cgi?searchType=miRNA&searchBox=hsa-miR-545-3p&full=1	AD (*n* = 21/15), preclinical AD (*n* = 21/15), PD (*n* = 21/0), Ctrl (*n* = 21/15)	Plasma, CSF<br>RT-qPCR	Expression levels of miR-15b-5p, miR-34a-5p, miR-142-3p, and miR-545-3p distinguished AD from PD and HC subjects.	[187]
			AD (*n* = 11/20), MCI (*n* = 9/0), Ctrl (*n* = 20/17)	Plasma<br>miRNA-seq/RT-qPCR	Expression of let-7d-5p, let-7g-5p, miR-15b-5p, miR-142-3p, miR-191-5p, miR-301a-3p and miR-545-3p deregulated in AD.	[291]

Epigenetic mechanism	Effect	Gene/Target pathway involved [1]	Study model [2]	Tissue Study/design	Main results	References
**miR-598**	↓	http://www.mirdb.org/cgi-bin/search.cgi?searchType=miRNA&searchBox=hsa-miR-598-5p&full=1	AD (*n* = 69); PD (*n* = 67); Ctrl (*n* = 78)	CSF<br>miRNA-seq	Expression of 41 miRNAs deregulated in AD CSF, and 20 miRNAs in AD serum.	[164]
	↑		AD (*n* = 10/18), Ctrl (*n* = 10/18)	CSF<br>RT-qPRC	Expression of 15 microRNAs deregulated in AD.<br>miR-9-5p and miR-598 suggested as potential biomarkers for AD.	[168]
**miR-1233-5p**	↓	http://www.mirdb.org/cgi-bin/search.cgi?searchType=miRNA&searchBox=hsa-miR-1233-5p&full=1	Aβ(−)MCI (*n* = 12), Aβ(+)MCI (*n* = 11), Ctrl (*n* = 9)	Platelets<br>Array/RT-qPCR	Expression of let-7i-5p, miR-125a, miR-1233-5p, and miR-6787-5p downregulated, and of miR-6880-5p upregulated in AD.	[292]

↓—decreased levels; ↑—increased levels; AD—Alzheimer's disease; BD—Bipolar disorder; CB—Cerebellum; CNS—Central nervous system; Ctrl—Control subjects; DEP—Depression; DLB—Dementia with Lewy bodies; FCx—Frontal cortex; FTD—Frontotemporal dementia; HPC—Hippocampus; HS-aging—Hippocampal sclerosis of aging; LOAD—Late-onset AD; MCI—Mild cognitive impairment; MFG—Medial frontal gyrus; aMCI—amnestic MCI; MS—Multiple sclerosis; *n*—Number of subjects; PD—Parkinson's disease; PCx—Parietal cortex; RT-qPCR—Reverse transcription quantitative real-time PCR; SCH—Schizophrenia; SMC—subjective memory complaints; TCx—Temporal cortex; VD—Vascular dementia; YOAD—Young-onset AD.

[1] Link to miRDB online database (http://www.mirdb.org/) which uses bioinformatics tool, MirTarget, for prediction of functional microRNA targets [313,314]

[2] Number of subjects per group is presented as Discovery cohort/Validation cohort

References

164. Burgos, K.; Malenica, I.; Metpally, R.; Courtright, A.; Rakela, B.; Beach, T.; Shill, H.; Adler, C.; Sabbagh, M.; Villa, S.; et al. Profiles of extracellular miRNA in cerebrospinal fluid and serum from patients with Alzheimer's and Parkinson's diseases correlate with disease status and features of pathology. *PLoS ONE* **2014**, *9*, e94839.
165. Cogswell, J.P.; Ward, J.; Taylor, I.A.; Waters, M.; Shi, Y.; Cannon, B.; Kelnar, K.; Kemppainen, J.; Brown, D.; Chen, C.; et al. Identification of miRNA changes in Alzheimer's disease brain and CSF yields putative biomarkers and insights into disease pathways. *J. Alzheimers Dis.* **2008**, *14*, 27–41.
166. Geekiyanage, H.; Chan, C. MicroRNA-137/181c regulates serine palmitoyltransferase and in turn amyloid beta, novel targets in sporadic Alzheimer's disease. *J. Neurosci.* **2011**, *31*, 14820–14830.
167. Hara, N.; Kikuchi, M.; Miyashita, A.; Hatsuta, H.; Saito, Y.; Kasuga, K.; Murayama, S.; Ikeuchi, T.; Kuwano, R. Serum microRNA miR-501-3p as a potential biomarker related to the progression of Alzheimer's disease. *Acta Neuropathol. Commun.* **2017**, *5*, 10.
168. Riancho, J.; Vázquez-Higuera, J.L.; Pozueta, A.; Lage, C.; Kazimierczak, M.; Bravo, M.; Calero, M.; Gonalezález, A.; Rodríguez, E.; Lleó, A.; et al. MicroRNA profile in patients with Alzheimer's disease: Analysis of miR-9-5p and miR-598 in raw and exosome enriched cerebrospinal fluid samples. *J. Alzheimers Dis.* **2017**, *57*, 483–491.
169. Alexandrov, P.N.; Dua, P.; Hill, J.M.; Bhattacharjee, S.; Zhao, Y.; Lukiw, W.J. MicroRNA (miRNA) speciation in Alzheimer's disease (AD) cerebrospinal fluid (CSF) and extracellular fluid (ECF). *Int. J. Biochem. Mol. Biol.* **2012**, *3*, 365–373.
170. Sethi, P.; Lukiw, W.J. Micro-RNA abundance and stability in human brain: Specific alterations in Alzheimer's disease temporal lobe neocortex. *Neurosci. Lett.* **2009**, *459*, 100–104.
171. Lukiw, W.J.; Alexandrov, P.N.; Zhao, Y.; Hill, J.M.; Bhattacharjee, S. Spreading of Alzheimer's disease inflammatory signaling through soluble micro-RNA. *Neuroreport* **2012**, *23*, 621–626.
172. Lukiw, W.J. Micro-RNA speciation in fetal, adult and Alzheimer's disease hippocampus. *Neuroreport* **2007**, *18*, 297–300.
173. Kiko, T.; Nakagawa, K.; Tsuduki, T.; Furukawa, K.; Arai, H.; Miyazawa, T. MicroRNAs in plasma and cerebrospinal fluid as potential markers for Alzheimer's disease. *J. Alzheimers Dis.* **2014**, *39*, 253–259.
174. Lusardi, T.A.; Phillips, J.I.; Wiedrick, J.T.; Harrington, C.A.; Lind, B.; Lapidus, J.A.; Quinn, J.F.; Saugstad, J.A. MicroRNAs in human cerebrospinal fluid as biomarkers for Alzheimer's disease. *J. Alzheimers Dis.* **2017**, *55*, 1223–1233.
175. Müller, M.; Jäkel, L.; Bruinsma, I.B.; Claassen, J.A.; Kuiperij, H.B.; Verbeek, M.M. MicroRNA-29a is a candidate biomarker for Alzheimer's disease in cell-free cerebrospinal fluid. *Mol. Neurobiol.* **2016**, *53*, 2894–2899.
176. Geekiyanage, H.; Jicha, G.A.; Nelson, P.T.; Chan, C. Blood serum miRNA: Non-invasive biomarkers for Alzheimer's disease. *Exp. Neurol.* **2012**, *235*, 491–496.
177. Lugli, G.; Cohen, A.M.; Bennett, D.A.; Shah, R.C.; Fields, C.J.; Hernandez, A.G.; Smalheiser, N.R. Plasma exosomal miRNAs in persons with and without Alzheimer disease: Altered expression and prospects for biomarkers. *PLoS ONE* **2015**, *10*, e0139233.
178. Satoh, J.; Kino, Y.; Niida, S. MicroRNA-Seq data analysis pipeline to identify blood biomarkers for Alzheimer's disease from public data. *Biomark Insights* **2015**, *10*, 21–31.
179. Villa, C.; Ridolfi, E.; Fenoglio, C.; Ghezzi, L.; Vimercati, R.; Clerici, F.; Marcone, A.; Gallone, S.; Serpente, M.; Cantoni, C.; et al. Expression of the transcription factor Sp1 and its regulatory hsa-miR-29b in peripheral blood mononuclear cells from patients with Alzheimer's disease. *J. Alzheimers Dis.* **2013**, *35*, 487–494.
180. Nunez-Iglesias, J.; Liu, C.C.; Morgan, T.E.; Finch, C.E.; Zhou, X.J. JoInt. genome-wide profiling of miRNA and mRNA expression in Alzheimer's disease cortex reveals altered miRNA regulation. *PLoS ONE* **2010**, *5*, e8898.
181. Yang, G.; Song, Y.; Zhou, X.; Deng, Y.; Liu, T.; Weng, G.; Yu, D.; Pan, S. DNA methyltransferase 3, a target of microRNA-29c, contributes to neuronal proliferation by regulating the expression of brain-derived neurotrophic factor. *Mol. Med. Rep.* **2015**, *12*, 1435–1442.
182. Wu, Y.; Xu, J.; Xu, J.; Cheng, J.; Jiao, D.; Zhou, C.; Dai, Y.; Chen, Q. Lower serum levels of miR-29c-3p and miR-19b-3p as biomarkers for Alzheimer's disease. *Tohoku J. Exp. Med.* **2017**, *242*, 129–136.
183. Gui, Y.; Liu, H.; Zhang, L.; Lv, W.; Hu, X. Altered microRNA profiles in cerebrospinal fluid exosome in Parkinson disease and Alzheimer disease. *Oncotarget* **2015**, *6*, 37043–37053.
184. Yang, G.; Song, Y.; Zhou, X.; Deng, Y.; Liu, T.; Weng, G.; Yu, D.; Pan, S. MicroRNA-29c targets beta-site amyloid precursor protein-cleaving enzyme 1 and has a neuroprotective role in vitro and in vivo. *Mol. Med. Rep.* **2015**, *12*, 3081–3088.
185. Lei, X.; Lei, L.; Zhang, Z.; Cheng, Y. Downregulated miR-29c correlates with increased BACE1 expression in sporadic Alzheimer's disease. *Int. J. Clin. Exp. Pathol.* **2015**, *8*, 1565–1574.

186. Sorensen, S.; Nygaard, A.; Christensen, T. miRNA expression profiles in cerebrospinal fluid and blood of patients with Alzheimer's disease and oTher. types of dementia—An exploratory study. *Transl. Neurodegener.* **2016**, *5*, 6.
187. Cosín-Tomás, M.; Antonell, A.; Lladó, A.; Alcolea, D.; Fortea, J.; Ezquerra, M.; Lleó, A.; Martí, M.J.; Pallàs, M.; Sanchez-Valle, R.; et al. Plasma miR-34a-5p and miR-545-3p as early biomarkers of Alzheimer's disease: Potential and limitations. *Mol. Neurobiol.* **2017**, *54*, 5550–5562.
188. Schipper, H.M.; Maes, O.C.; Chertkow, H.M.; Wang, E. MicroRNA expression in Alzheimer blood mononuclear cells. *Gene Regul. Syst. Biol.* **2007**, *1*, 263–274.
189. Pogue, A.I.; Lukiw, W.J. Up-regulated pro-inflammatory microRNAs (miRNAs) in Alzheimer's disease (AD) and age-related macular degeneration (AMD). *Cell Mol. NeuroBiol.* **2018**, *38*, 1021–1031.
190. Agostini, M.; Tucci, P.; Killick, R.; Candi, E.; Sayan, B.S.; di Val Cervo, P.R.; Nicotera, P.; McKeon, F.; Knight, R.A.; Mak, T.W.; et al. Neuronal differentiation by TAp73 is mediated by microRNA-34a regulation of synaptic protein targets. *Proc. Natl. Acad. Sci. USA* **2011**, *108*, 21093–21098.
191. Zhao, Y.; Bhattacharjee, S.; Jones, B.M.; Dua, P.; Alexandrov, P.N.; Hill, J.M.; Lukiw, W.J. Regulation of TREM2 expression by NF-κB-sensitive miRNA-34a. *Neuroreport* **2013**, *24*, 318–323.
192. Leidinger, P.; Backes, C.; Deutscher, S.; Schmitt, K.; Mueller, S.; Frese, K.; Haas, J.; Ruprecht, K.; Paul, F.; Stahler, C.; et al. A blood based 12-miRNA signature of Alzheimer disease patients. *Genome Biol.* **2013**, *14*, R78.
193. Wang, T.; Chen, K.; Li, H.; Dong, S.; Su, N.; Liu, Y.; Cheng, Y.; Dai, J.; Yang, C.; Xiao, S. The feasibility of utilizing plasma MiRNA107 and BACE1 messenger RNA gene expression for clinical diagnosis of amnestic mild cognitive impairment. *J. Clin. Psychiatry* **2015**, *76*, 135–141.
194. Wang, W.X.; Rajeev, B.W.; Stromberg, A.J.; Ren, N.; Tang, G.; Huang, Q.; Rigoutsos, I.; and Nelson, P.T. The expression of microRNA miR-107 decreases early in Alzheimer's disease and may accelerate disease progression through regulation of beta-site amyloid precursor protein-cleaving enzyme 1. *J. Neurosci.* **2008**, *28*, 1213–1223.
195. Moncini, S.; Lunghi, M.; Valmadre, A.; Grasso, M.; Vescovo, V.D.; Riva, P.; Denti, M.A.; Venturin, M. The miR-15/107 family of microRNA genes regulates CDK5R1/p35 with implications for Alzheimer's disease pathogenesis. *Mol. NeuroBiol.* **2017**, *54*, 4329–4342.
196. Müller, M.; Kuiperij, H.B.; Claassen, J.A.; Kusters, B.; Verbeek, M.M. MicroRNAs in Alzheimer's disease: Differential expression in hippocampus and cell-free cerebrospinal fluid. *Neurobiol. Aging* **2014**, *35*, 152–158.
197. Galimberti, D.; Villa, C.; Fenoglio, C.; Serpente, M.; Ghezzi, L.; Cioffi, S.M.; Arighi, A.; Fumagalli, G.; Scarpini, E. Circulating miRNAs as potential biomarkers in Alzheimer's disease. *J. Alzheimers Dis.* **2014**, *42*, 1261–1267.
198. Tan, L.; Yu, J.T.; Tan, M.S.; Liu, Q.Y.; Wang, H.F.; Zhang, W.; Jiang, T.; Tan, L. Genome-wide serum microRNA expression profiling identifies serum biomarkers for Alzheimer's disease. *J. Alzheimers Dis.* **2014**, *40*, 1017–1027.
199. Dangla-Valls, A.; Molinuevo, J.L.; Altirriba, J.; Sanchez-Valle, R.; Alcolea, D.; Fortea, J.; Rami, L.; Balasa, M.; Muñoz-García, C.; Ezquerra, M.; et al. CSF microRNA profiling in Alzheimer's disease: A screening and validation study. *Mol. Neurobiol.* **2017**, *54*, 6647–6654.
200. McKeever, P.M.; Schneider, R.; Taghdiri, F.; Weichert, A.; Multani, N.; Brown, R.A.; Boxer, A.L.; Karydas, A.; Miller, B.; Robertson, J.; et al. MicroRNA expression levels are altered in the cerebrospinal fluid of patients with young-onset Alzheimer's disease. *Mol. Neurobiol.* **2018**, *55*, 8826–8841.
201. Cha, D.; Mengel, D.; Mustapic, M.; Liu, W.; Selkoe, D.J.; Kapogiannis, D.; Galasko, D.; Rissman, R.A.; Bennett, D.A.; Walsh, D.M. miR-212 and miR-132 are downregulated in neurally derived plasma exosomes of Alzheimer's patients. *Front. Neurosci.* **2019**, *13*, 1208.
202. Annese, A.; Manzari, C.; Lionetti, C.; Picardi, E.; Horner, D.S.; Chiara, M.; Caratozzolo, M.F.; Tullo, A.; Fosso, B.; Pesole, G.; et al. Whole transcriptome profiling of late-onset Alzheimer's disease patients provides insights into the molecular changes involved in the disease. *Sci. Rep.* **2018**, *8*, 4282.
203. Hebert, S.S.; Wang, W.-X.; Zhu, Q.; Nelson, P.T. A study of small RNAs from cerebral neocortex of pathology-verified Alzheimer's disease, dementia with Lewy bodies, hippocampal sclerosis, frontotemporal lobar dementia, and non-demented human controls. *J. Alzheimers Dis.* **2013**, *35*, 335–348.
204. Lau, P.; Bossers, K.; Janky, R.S.; Salta, E.; Frigerio, C.S.; Barbash, S.; Rothman, R.; Sierksma, A.S.; Thathiah, A.; Greenberg, D.; et al. Alteration of the microRNA network during the progression of Alzheimer's disease. *EMBO Mol. Med.* **2013**, *5*, 1613–1634.
205. Smith, P.Y.; Hernandez-Rapp, J.; Jolivette, F.; Lecours, C.; Bisht, K.; Goupil, C.; Dorval, V.; Parsi, S.; Morin, F.; Planel, E.; et al. miR-132/212 deficiency impairs tau metabolism and promotes pathological aggregation in vivo. *Hum. Mol. Genet.* **2015**, *24*, 6721–6735.
206. Weinberg, R.B.; Mufson, E.J.; Counts, S.E. Evidence for a neuroprotective microRNA pathway in amnestic mild cognitive impairment. *Front. Neurosci.* **2015**, *9*, 430.

207. Wong, H.K.; Veremeyko, T.; Patel, N.; Lemere, C.A.; Walsh, D.M.; Esau, C.; Vanderburg, C.; Krichevsky, A.M. De-repression of FOXO3a death axis by microRNA-132 and -212 causes neuronal apoptosis in Alzheimer's disease. *Hum. Mol. Genet.* **2013**, *22*, 3077–3092.
208. Xie, B.; Zhou, H.; Zhang, R.; Song, M.; Yu, L.; Wang, L.; Liu, Z.; Zhang, Q.; Cui, D.; Wang, X.; et al. Serum miR-206 and miR-132 as potential circulating biomarkers for mild cognitive impairment. *J. Alzheimers Dis.* **2015**, *45*, 721–731.
209. Dong, H.; Li, J.; Huang, L.; Chen, X.; Li, D.; Wang, T.; Hu, C.; Xu, J.; Zhang, C.; Zen, K.; et al. Serum microRNA profiles serve as novel biomarkers for the diagnosis of Alzheimer's disease. *Dis. Markers* **2015**, *2015*, 625659.
210. Wu, H.Z.Y.; Thalamuthu, A.; Cheng, L.; Fowler, C.; Masters, C.L.; Sachdev, P.; Mather, K.A. The Australian Imaging Biomarkers and Lifestyle Flagship Study of Ageing. Differential blood miRNA expression in brain amyloid imaging-defined Alzheimer's disease and controls. *Alzheimers Res. Ther.* **2020**, *12*, 59.
211. Denk, J.; Boelmans, K.; Siegismund, C.; Lassner, D.; Arlt, S.; Jahn, H. MicroRNA profiling of CSF reveals potential biomarkers to detect Alzheimer`s disease. *PLoS ONE* **2015**, *10*, e0126423.
212. Cui, J.G.; Li, Y.Y.; Zhao, Y.; Bhattacharjee, S.; Lukiw, W.J. Differential regulation of interleukin-1 receptor-associated kinase-1 (IRAK-1) and IRAK-2 by microRNA-146a and NFkappaB in stressed human astroglial cells and in Alzheimer disease. *J. Biol. Chem.* **2010**, *285*, 38951–38960.
213. Zhao, Y.; Alexandrov, P.N.; Jaber, V.; Lukiw, W.J. Deficiency in the ubiquitin conjugating enzyme UBE2A in Alzheimer's disease (AD) is linked to deficits in a natural circular miRNA-7 sponge (circRNA; ciRS-7). *Genes* **2016**, *7*, 12.
214. Lukiw, W.J.; Zhao, Y.; Cui, J.G. An NF-kappaB-sensitive microRNA-146a-mediated inflammatory circuit in Alzheimer disease and in stressed human brain cells. *J. Biol. Chem.* **2008**, *283*, 31315–31322.
215. Guedes, J.R.; Santana, I.; Cunha, C.; Duro, D.; Almeida, M.R.; Cardoso, A.M.; de Lima, M.C.; Cardoso, A.L. MicroRNA deregulation and chemotaxis and phagocytosis impairment in Alzheimer's disease. *Alzheimers Dement.* **2016**, *3*, 7–17.
216. Siedlecki-Wullich, D.; Catala-Solsona, J.; Fabregas, C.; Hernandez, I.; Clarimon, J.; Lleo, A.; Boada, M.; Saura, C.A.; Rodríguez-Álvarez, J.; Miñano-Molina, A.J. Altered microRNAs related to synaptic function as potential plasma biomarkers for Alzheimer's disease. *Alzheimers Res. Ther.* **2019**, *11*, 46.
217. Kenny, A.; McArdle, H.; Calero, M.; Rabano, A.; Madden, S.F.; Adamson, K.; Forster, R.; Spain, E.; Prehn, J.H.M.; Henshall, D.C.; et al. Elevated plasma microRNA-206 levels predict cognitive decline and progression to dementia from mild cognitive impairment. *Biomolecules* **2019**, *9*, 734.
218. van Harten, A.C.; Mulders, J.; Scheltens, P.; van der Flier, W.M.; Oudejans, C.B. Differential expression of microRNA in cerebrospinal fluid as a potential novel biomarker for Alzheimer's disease *J. Alzheimers Dis.* **2015**, *47*, 243–252.
219. Aydemir, Y.; Simkin, A.; Gascon, E.; Gao, F.B. MicroRNA-9: Functional evolution of a conserved small regulatory RNA. *RNA Biol.* **2011**, *8*, 557–564.
220. Holohan, K.N.; Lahiri, D.K.; Schneider, B.P.; Foroud, T.; Saykin, A.J. Functional microRNAs in Alzheimer's disease and cancer: Differential regulation of common mechanisms and pathways. *Front. Genet.* **2012**, *3*, 323.
221. Shaik, M.M.; Tamargo, I.A.; Abubakar, M.B.; Kamal, M.A.; Greig, N.H.; Gan, S.H. The role of microRNAs in Alzheimer's disease and their therapeutic potentials. *Genes* **2018**, *9*, 174.
222. Chang, F.; Zhang, L.H.; Xu, W.P.; Jing, P.; Zhan, P.Y. microRNA-9 attenuates amyloidβ-induced synaptotoxicity by targeting calcium/calmodulin-dependent protein kinase kinase 2. *Mol. Med. Rep.* **2014**, *9*, 1917–1922.
223. Mairet-Coello, G.; Courchet, J.; Pieraut, S.; Courchet, V.; Maximov, A.; Polleux, F. The CAMKK2-AMPK kinase pathway mediates the synaptotoxic effects of Aβ oligomers through Tau phosphorylation. *Neuron* **2013**, *78*, 94–108.
224. Bettens, K.; Brouwers, N.; Engelborghs, S.; Van Miegroet, H.; De Deyn, P.P.; Theuns, J.; Sleegers, K.; Van Broeckhoven, C. APP and BACE1 miRNA genetic variability has no major role in risk for Alzheimer disease. *Hum. Mutat.* **2009**, *30*, 1207–1213.
225. Zong, Y.; Wang, H.; Dong, W.; Quan, X.; Zhu, H.; Xu, Y.; Huang, L.; Ma, C.; Qin, C. miR-29c regulates BACE1 protein expression. *Brain Res.* **2011**, *1395*, 108–115.
226. Shioya, M.; Obayashi, S.; Tabunoki, H.; Arima, K.; Saito, Y.; Ishida, T.; Satoh, J. Aberrant microRNA expression in the brains of neurodegenerative diseases: miR-29a decreased in Alzheimer disease brains targets neurone navigator 3. *Neuropathol. Appl. Neurobiol.* **2010**, *36*, 320–330.
227. Kole, A.J.; Swahari, V.; Hammond, S.M.; Deshmukh, M. miR-29b is activated during neuronal maturation and targets BH3-only genes to restrict apoptosis. *Genes Dev.* **2011**, *25*, 125–130.
228. Wang, X. The expanding role of mitochondria in apoptosis. *Genes Dev.* **2001**, *15*, 2922–2933.

229. Rohn, T.T.; Vyas, V.; Hernandez-Estrada, T.; Nichol, K.E.; Christie, L.A.; Head, E. Lack of pathology in a triple transgenic mouse model of Alzheimer's disease after overexpression of the anti-apoptotic protein Bcl-2. *J. Neurosci.* **2008**, *28*, 3051–3059.
230. Howard, S.; Bottino, C.; Brooke, S.; Cheng, E.; Giffard, R.G.; Sapolsky, R. Neuroprotective effects of bcl-2 overexpression in hippocampal cultures: Interactions with pathways of oxidative damage. *J. Neurochem.* **2002**, *83*, 914–923.
231. Li, L.H.; Tu, Q.Y.; Deng, X.H.; Xia, J.; Hou, D.R.; Guo, K.; Zi, X.H. Mutant presenilin2 promotes apoptosis through the p53/miR-34a axis in neuronal cells. *Brain Res.* **2017**, *1662*, 57–64.
232. Wang, X.; Liu, P.; Zhu, H.; Xu, Y.; Ma, C.; Dai, X.; Huang, L.; Liu, Y.; Zhang, L.; Qin, C. miR-34a, a microRNA up-regulated in a double transgenic mouse model of Alzheimer's disease, inhibits BCL2 translation. *Brain Res. Bull.* **2009**, *80*, 268–273.
233. Dickson, J.R.; Kruse, C.; Montagna, D.R.; Finsen, B.; Wolfe, M.S. Alternative polyadenylation and miR-34 family members regulate tau expression. *J. Neurochem.* **2013**, *127*, 739–749.
234. Nelson, P.T.; Wang, W.X. MiR-107 is reduced in Alzheimer's disease brain neocortex: Validation study. *J. Alzheimers Dis.* **2010**, *21*, 75–79.
235. Van Damme, P.; Van Hoecke, A.; Lambrechts, D.; Vanacker, P.; Bogaert, E.; van Swieten, J.; Carmeliet, P.; Van Den Bosch, L.; Robberecht, W. Progranulin functions as a neurotrophic factor to regulate neurite outgrowth and enhance neuronal survival. *J. Cell Biol.* **2008**, *181*, 37–41.
236. Wang, W.X.; Wilfred, B.R.; Madathil, S.K.; Tang, G.; Hu, Y.; Dimayuga, J.; Stromberg, A.J.; Huang, Q.; Saatman, K.E.; Nelson, P.T. miR-107 regulates granulin/progranulin with implications for traumatic brain injury and neurodegenerative disease. *Am. J. Pathol.* **2010**, *177*, 334–345.
237. Yao, J.; Hennessey, T.; Flynt, A.; Lai, E.; Beal, M.F.; Lin, M.T. MicroRNA-related cofilin abnormality in Alzheimer's disease. *PLoS ONE* **2010**, *5*, e15546.
238. Moncini, S.; Salvi, A.; Zuccotti, P.; Viero, G.; Quattrone, A.; Barlati, S.; De Petro, G.; Venturin, M.; Riva, P. The role of miR-103 and miR-107 in regulation of CDK5R1 expression and in cellular migration. *PLoS ONE* **2011**, *6*, e20038.
239. Rademakers, R.; Sleegers, K.; Theuns, J.; Van den Broeck, M.; Bel Kacem, S.; Nilsson, L.-G.; Adolfsson, R.; van Duijn, C.M.; Van Broeckhoven, C.; Cruts, M. Association of cyclin-dependent kinase 5 and neuronal activators p35 and p39 complex in early-onset Alzheimer's disease. *Neurobiol. Aging* **2005**, *26*, 1145–1151.
240. Augustin, R.; Endres, K.; Reinhardt, S.; Kuhn, P.H.; Lichtenthaler, S.F.; Hansen, J.; Wurst, W.; Trümbach, D. Computational identification and experimental validation of microRNAs binding to the Alzheimer-related gene ADAM10. *BMC Med. Genet.* **2012**, *13*, 35.
241. Hansen, K.F.; Sakamoto, K.; Aten, S.; Snider, K.H.; Loeser, J.; Hesse, A.M.; Page, C.E.; Pelz, C.; Arthur, J.S.; Impey, S.; et al. Targeted deletion of miR-132/-212 impairs memory and alters the hippocampal transcriptome. *Learn. Mem.* **2016**, *23*, 61–71.
242. Lukiw, W.J. NF-κB-regulated micro RNAs (miRNAs) in primary human brain cells. *Exp. Neurol.* **2012**, *235*, 484–490.
243. Li, Y.Y.; Cui, J.G.; Dua, P.; Pogue, A.I.; Bhattacharjee, S.; Lukiw, W.J. Differential expression of miRNA-146a-regulated inflammatory genes in human primary neural, astroglial and microglial cells. *Neurosci. Lett.* **2011**, *499*, 109–113.
244. Fernandes, A.; Ribeiro, A.R.; Monteiro, M.; Garcia, G.; Vaz, A.R.; Brites, D. Secretome from SH-SY5Y APPSwe cells trigger time-dependent CHME3 microglia activation phenotypes, ultimately leading to miR-21 exosome shuttling. *Biochimie* **2018**, *155*, 67–82.
245. Li, J.J.; Wang, B.; Kodali, M.C.; Chen, C.; Kim, E.; Patters, B.J.; Lan, L.; Kumar, S.; Wang, X.; Yue, J.; et al. In vivo evidence for the contribution of peripheral circulating inflammatory exosomes to neuroinflammation. *J. Neuroinflamm.* **2018**, *15*, 8.
246. Song, J.; Lee, J.E. miR-155 is involved in Alzheimer's disease by regulating T lymphocyte function. *Front. Aging Neurosci.* **2015**, *7*, 61.
247. Schonrock, N.; Ke, Y.D.; Humphreys, D.; Staufenbiel, M.; Ittner, L.M.; Preiss, T.; Götz, J. Neuronal microRNA deregulation in response to Alzheimer's disease amyloid-beta. *PLoS ONE* **2010**, *5*, e11070.
248. Schonrock, N.; Humphreys, D.T.; Preiss, T.; Götz, J. Target gene repression mediated by miRNAs miR-181c and miR-9 both of which are down-regulated by amyloid-β. *J. Mol. Neurosci.* **2012**, *46*, 324–335.
249. Hutchison, E.R.; Kawamoto, E.M.; Taub, D.D.; Lal, A.; Abdelmohsen, K.; Zhang, Y.; Wood, W.H., 3rd.; Lehrmann, E.; Camandola, S.; Becker, K.G.; et al. Evidence for miR-181 involvement in neuroinflammatory responses of astrocytes. *Glia* **2013**, *61*, 1018–1028.
250. Lee, S.T.; Chu, K.; Jung, K.H.; Kim, J.H.; Huh, J.Y.; Yoon, H.; Park, D.-K.; Lim, J.-Y.; Kim, J.-M.; Daejong, J.; et al. miR-206 regulates brain-derived neurotrophic factor in Alzheimer disease model. *Ann. Neurol.* **2012**, *72*, 269–277.

251. Tian, N.; Cao, Z.; Zhang, Y. MiR-206 decreases brain-derived neurotrophic factor levels in a transgenic mouse model of Alzheimer's disease. *Neurosci. Bull.* **2014**, *30*, 191–197.
252. Wang, C.N.; Wang, Y.J.; Wang, H.; Song, L.; Chen, Y.; Wang, J.L.; Ye, Y.; Jiang, B. The anti-dementia effects of donepezil involve miR-206-3p in the hippocampus and cortex. *Biol. Pharm Bull.* **2017**, *40*, 465–472.
253. Chuang, D.-M.; Leng, Y.; Marinova, Z.; Kim, H.-J.; Chiu, C.-T. Multiple roles of HDAC inhibition in neurodegenerative conditions. *Trends Neurosci.* **2009**, *32*, 591–601.
254. Cuadrado-Tejedor, M.; Oyarzabal, J.; Pascual Lucas, M.; Franco, R.; Garcia-Osta, A. Epigenetic drugs in Alzheimer's disease. *Biomol. Concepts* **2013**, *4*, 433–445.
255. Esposito, M.; Sherr, G.L. Epigenetic modifications in Alzheimer's neuropathology and therapeutics. *Front. Neurosci.* **2019**, *13*, 476.
256. Seo, S.B.; McNamara, P.; Heo, S.; Turner, A.; Lane, W.S.; Chakravarti, D. Regulation of histone acetylation and transcription by INHAT, a human cellular complex containing the set oncoprotein. *Cell* **2001**, *104*, 119–130.
257. Tsujio, I.; Zaidi, T.; Xu, J.; Kotula, L.; Grundke-Iqbal, I.; Iqbal, K. Inhibitors of protein phosphatase-2A from human brain structures, immunocytological localization and activities towards dephosphorylation of the Alzheimer type hyperphosphorylated tau. *FEBS Lett.* **2005**, *579*, 363–372.
258. Tanimukai, H.; Grundke-Iqbal, I.; Iqbal, K. Upregulation of inhibitors of protein phosphatase-2A in Alzheimer's disease. *Am. J. Pathol.* **2005**, *166*, 1761–1771.
259. Chai, G.S.; Feng, Q.; Wang, Z.H.; Hu, Y.; Sun, D.-S.; Li, X.-G.; Ke, D.; Li, H.-L.; Liu, G.-P.; Wang, J.-Z. Downregulating ANP32A rescues synapse and memory loss via chromatin remodeling in Alzheimer model. *Mol. Neurodegener.* **2017**, *12*, 34.
260. Hanna, J.; Hossain, G.S.; Kocerha, J. The potential for microRNA therapeutics and clinical research. *Front. Genet.* **2019**, *10*, 478.
261. Zhao, Y.; Alexandrov, P.N.; Lukiw, W.J. Anti-microRNAs as novel therapeutic agents in the clinical management of Alzheimer's disease. *Front. Neurosci.* **2016**, *10*, 59.
262. Paul, S.; Bravo Vázquez, L.A.; Pérez Uribe, S.; Roxana Reyes-Pérez, P.; Sharma, A. current status of microRNA-based therapeutic approaches in neurodegenerative disorders. *Cells* **2020**, *9*, 1698.
263. Kim, H.; Kim, J.S. A guide to genome engineering with programmable nucleases. *Nat. Rev. Genet.* **2014**, *15*, 321–334.
264. Barman, N.C.; Khan, N.M.; Islam, M.; Nain, Z.; Roy, R.K.; Haque, A.; Barman, S.K. CRISPR-Cas9: A promising genome editing therapeutic tool for Alzheimer's disease-a narrative review. *Neurol. Ther.* **2020**, *9*, 419–434.
265. Cota-Coronado, A.; Díaz-Martínez, N.F.; Padilla-Camberos, E.; Díaz-Martínez, N.E. Editing the central nervous system through CRISPR/Cas9 systems. *Front. Mol. Neurosci.* **2019**, *1*, 110.
266. Rohn, T.T.; Kim, N.; Isho, N.F.; & Mack, J.M. The potential of CRISPR/Cas9 gene editing as a treatment strategy for Alzheimer's disease. *J. Alzheimers Dis. Parkinsonism* **2018**, *8*, 439.
267. Jinek, M.; Chylinski, K.; Fonfara, I.; Hauer, M.; Doudna, J.A.; Charpentier, E. A programmable dual-RNA-guided DNA endonuclease in adaptive bacterial immunity. *Science* **2012**, *337*, 816–821.
268. Dominguez, A.A.; Lim, W.A.; and Qi, L.S. Beyond editing: Repurposing CRISPR-Cas9 for precision genome regulation and interrogation. *Nat. Rev. Mol. Cell Biol.* **2016**, *17*, 5–15.
269. Kearns, N.A.; Pham, H.; Tabak, B.; Genga, R.M.; Silverstein, N.J.; Garber, M.; Maehr, R. Functional annotation of native enhancers with a Cas9-histone demethylase fusion. *Nat. Methods* **2015**, *12*, 401–403.
270. Hilton, I.B.; D'Ippolito, A.M.; Vockley, C.M.; Thakore, P.I.; Crawford, G.E.; Reddy, T.E.; Gersbach, C.A. Epigenome editing by a CRISPR-Cas9-based acetyltransferase activates genes from promoters and enhancers. *Nat. Biotechnol.* **2015**, *33*, 510–517.
271. Cano-Rodriguez, D.; Gjaltema, R.A.; Jilderda, L.J.; Jellema, P.; Dokter-Fokkens, J.; Ruiters, M.H.; Rots, M.G. Writing of H3K4Me3 overcomes epigenetic silencing in a sustained but context-dependent manner. *Nat. Commun.* **2016**, *7*, 12284.
272. Kwon, D.Y.; Zhao, Y.T.; Lamonica, J.M.; Zhou, Z. Locus-specific histone deacetylation using a synthetic CRISPR-Cas9-based HDAC. *Nat. Commun.* **2017**, *8*, 15315.
273. Lei, Y.; Zhang, X.; Su, J.; Jeong, M.; Gundry, M.C.; Huang, Y.H.; Zhou, Y.; Li, W.; Goodell, M.A. Targeted DNA methylation in vivo using an engineered dCas9-MQ1 fusion protein. *Nat. Commun.* **2017**, *8*, 16026.
274. Liu, X.S.; Wu, H.; Ji, X.; Stelzer, Y.; Wu, X.; Czauderna, S.; Shu, J.; Dadon, D.; Young, R.A.; Jaenisch, R. Editing DNA Methylation in the Mammalian Genome. *Cell* **2016**, *167*, 233–247 e217.
275. McDonald, J.I.; Celik, H.; Rois, L.E.; Fishberger, G.; Fowler, T.; Rees, R.; Kramer, A.; Martens, A.; Edwards, J.R.; Challen, G.A. Reprogrammable CRISPR/Cas9-based system for inducing site-specific DNA methylation. *Biol. Open* **2016**, *5*, 866–874.

276. Vojta, A.; Dobrinic, P.; Tadic, V.; Bockor, L.; Korac, P.; Julg, B.; Klasic, M.; Zoldos, V. Repurposing the CRISPR-Cas9 system for targeted DNA methylation. *Nucleic Acids Res.* **2016**, *44*, 5615–5628.
277. Xiong, T.; Meister, G.E.; Workman, R.E.; Kato, N.C.; Spellberg, M.J.; Turker, F.; Timp, W.; Ostermeier, M.; Novina, C.D. Targeted DNA methylation in human cells using engineered dCas9-methyltransferases. *Sci. Rep.* **2017**, *7*, 6732.
278. Choudhury, S.R.; Cui, Y.; Lubecka, K.; Stefanska, B.; Irudayaraj, J. CRISPR-dCas9 mediated TET1 targeting for selective DNA demethylation at BRCA1 promoter. *Oncotarget* **2016**, *7*, 46545–46556.
279. Morita, S.; Noguchi, H.; Horii, T.; Nakabayashi, K.; Kimura, M.; Okamura, K.; Sakai, A.; Nakashima, H.; Hata, K.; Nakashima, K.; et al. Targeted DNA demethylation in vivo using dCas9-peptide repeat and scFv-TET1 catalytic domain fusions. *Nat. Biotechnol.* **2016**, *34*, 1060–1065.
280. Xu, X.; Tao, Y.; Gao, X.; Zhang, L.; Li, X.; Zou, W.; Ruan, K.; Wang, F.; Xu, G.L.; Hu, R. A CRISPR based approach for targeted DNA demethylation. *Cell Discov.* **2016**, *2*, 16009.
281. Shechner, D.M.; Hacisuleyman, E.; Younger, S.T.; Rinn, J.L. Multiplexable, locus-specific targeting of long RNAs with CRISPR-Display. *Nat. Methods* **2015**, *12*, 664–670.
282. Manev, H.; Dzitoyeva, S. Progress in mitochondrial epigenetics. *Biomol. Concepts* **2013**, *4*, 381–389.
283. Mattsson, N. CSF biomarkers in neurodegenerative diseases. *Clin. Chem. Lab. Med.* **2011**, *49*, 345–352.
284. Johansson, P.; Mattsson, N.; Hansson, O.; Wallin, A.; Johansson, J.O.; Andreasson, U.; Zetterberg, H.; Blennow, K.; Svensson, J. Cerebrospinal fluid biomarkers for Alzheimer's disease: Diagnostic performance in a homogeneous mono-center population. *J. Alzheimers Dis.* **2011**, *24*, 537–546.
285. Toraño, E.G.; García, M.G.; Fernández-Morera, J.L.; Niño-García, P.; Fernández, A.F. The impact of external factors on the epigenome: In utero and over lifetime. *Biomed. Res. Int.* **2016**, 2568635.
286. Kurdyukov, S.; Bullock, M. DNA methylation analysis: Choosing the right method. *Biology* **2016**, *5*, 3.
287. Zhang, Y.H.; Bai, S.F.; Yan, J.Q. Blood circulating miRNAs as biomarkers of Alzheimer's disease: A systematic review and meta-analysis. *Biomark Med.* **2019**, *13*, 1045–1054.
288. Podlesniy, P.; Llorens, F.; Golanska, E.; Sikorska, B.; Liberski, P.; Zerr, I.; Trullas, R. Mitochondrial DNA differentiates Alzheimer's disease from Creutzfeldt-Jakob disease. *Alzheimers Dement.* **2016**, *12*, 546–555.
289. Sheng, B.; Wang, X.; Su, B.; Lee, H.G.; Casadesus, G.; Perry, G.; Zhu, X. Impaired mitochondrial biogenesis contributes to mitochondrial dysfunction in Alzheimer's disease. *J. Neurochem.* **2012**, *120*, 419–429.
290. Liu, Y.; He, X.; Li, Y.; Wang, T. Cerebrospinal fluid CD4+ T lymphocyte-derived miRNA-let-7b can enhances the diagnostic performance of Alzheimer's disease biomarkers. *Biochem. Biophys. Res. Commun.* **2018**, *495*, 1144–1150.
291. Kumar, P.; Dezso, Z.; MacKenzie, C.; Oestreicher, J.; Agoulnik, S.; Byrne, M.; Bernier, F.; Yanagimachi, M.; Aoshima, K.; Oda, Y. Circulating miRNA biomarkers for Alzheimer's disease. *PLoS ONE* **2013**, *8*, e69807.
292. Lee, B.K.; Kim, M.H.; Lee, S.Y.; Son, S.J.; Hong, C.H.; Jung, Y.S. Downregulated platelet miR-1233-5p in patients with Alzheimer's pathologic change with mild cognitive impairment is associated with Aβ-induced platelet activation via P-Selectin. *J. Clin. Med.* **2020**, *9*, 1624.
293. Cheng, L.; Doecke, J.D.; Sharples, R.A.; Villemagne, V.L.; Fowler, C.J.; Rembach, A.; Martins, R.N.; Rowe, C.C.; Macaulay, S.L.; Masters, C.L.; et al. Prognostic serum miRNA biomarkers associated with Alzheimer's disease shows concordance with neuropsychological and neuroimaging assessment. *Mol. Psychiatry* **2015**, *20*, 1188–1196.
294. Guo, R.; Fan, G.; Zhang, J.; Wu, C.; Du, Y.; Ye, H.; Li, Z.; Wang, L.; Zhang, Z.; Zhang, L.; et al. A 9-microRNA signature in serum serves as a noninvasive biomarker in early diagnosis of Alzheimer's disease. *J. Alzheimers Dis.* **2017**, *60*, 1365–1377.
295. Sala Frigerio, C.; Lau, P.; Salta, E.; Tournoy, J.; Bossers, K.; Vandenberghe, R.; Wallin, A.; Bjerke, M.; Zetterberg, H.; Blennow, K.; et al. Reduced expression of hsa-miR-27a-3p in CSF of patients with Alzheimer disease. *Neurology* **2013**, *81*, 2103–2106.
296. Bhatnagar, S.; Chertkow, H.; Schipper, H.M.; Yuan, Z.; Shetty, V.; Jenkins, S.; Jones, T.; Wang, E. Increased microRNA-34c abundance in Alzheimer's disease circulating blood plasma. *Front. Mol. Neurosci.* **2014**, *7*, 2.
297. Shi, Z.; Zhang, K.; Zhou, H.; Jiang, L.; Xie, B.; Wang, R.; Xia, W.; Yin, Y.; Gao, Z.; Cui, D.; et al. Increased miR-34c mediates synaptic deficits by targeting synaptotagmin 1 through ROS-JNK-p53 pathway in Alzheimer's disease. *Aging Cell* **2020**, *19*, e13125.
298. Nagaraj, S.; Laskowska-Kaszub, K.; Dębski, K.J.; Wojsiat, J.; Dąbrowski, M.; Gabryelewicz, T.; Kuźnicki, J.; Wojda, U. Profile of 6 microRNA in blood plasma distinguish early-stage Alzheimer's disease patients from non-demented subjects. *Oncotarget* **2017**, *8*, 16122–16143.
299. Madadi, S.; Saidijam, M.; Yavari, B.; Soleimani, M. Downregulation of serum miR-106b: A potential biomarker for Alzheimer disease. *Arch. Physiol. Biochem.* **2020**, *6*, 1–5.

300. Piscopo, P.; Grasso, M.; Puopolo, M.; D'Acunto, E.; Talarico, G.; Crestini, A.; Gasparini, M.; Campopiano, R.; Gambardella, S.; Castellano, A.E.; et al. Circulating miR-127-3p as a potential biomarker for differential diagnosis in frontotemporal dementia. *J. Alzheimers Dis.* **2018**, *65*. 455–464.
301. Culpan, D.; Kehoe, P.G.; Love, S. Tumour necrosis factoralpha (TNF-alpha) andmiRNA expression in frontal and temporal neocortex in Alzheimer's disease and the effect of TNF-alpha on miRNA expression in vitro. *Int. J. Mol. Epidemiol Genet.* **2011**, *2*, 156–162.
302. Yang, Q.; Zhao, Q.; Yin, Y. miR-133b is a potential diagnostic biomarker for Alzheimer's disease and has a neuroprotective role. *Exp. Ther. Med.* **2019**, *18*, 2711–2718.
303. Liu, C.G.; Wang, J.L.; Li, L.; Xue, L.X.; Zhang, Y.Q.; Wang, P.C. MicroRNA-135a and -200b, potential biomarkers for Alzheimer's disease, regulate β secretase and amyloid precursor protein. *Brain Res.* **2014**, *1583*, 55–64.
304. Yang, T.T.; Liu, C.G.; Gao, S.C.; Zhang, Y.; Wang, P.C. The serum exosome derived MicroRNA-135a, -193b, and -384 were potential Alzheimer's disease biomarkers. *Biomed. Environ. Sci.* **2018**, *31*, 87–96.
305. Cao, F.; Liu, Z.; Sun, G. Diagnostic value of miR-193a-3p in Alzheimer's disease and miR-193a-3p attenuates amyloid-beta induced neurotoxicity by targeting PTEN. *Exp. Gerontol.* **2020**, *130*, 110814.
306. Wang, L.; Liu, J.; Wang, Q.; Jiang, H.; Zeng, L.; Li, Z.; Liu, R. MicroRNA-200a-3p mediates neuroprotection in Alzheimer-related deficits and attenuates amyloid-beta overproduction and tau hyperphosphorylation via coregulating BACE1 and PRKACB. *Front. Pharmacol.* **2019**, *10*, 806.
307. Zeng, Q.; Zou, L.; Qian, L.; Zhou, F.; Nie, H.; Yu, S.; Jiang, J.; Zhuang, A.; Wang, C.; Zhang, H. Expression of microRNA-222 in serum of patients with Alzheimer's disease. *Mol. Med. Rep.* **2017**, *16*, 5575–5579.
308. Marchegiani, F.; Matacchione, G.; Ramini, D.; Marcheselli, F.; Recchioni, R.; Casoli, T.; Mercuri, E.; Lazzarini, M.; Giorgetti, B.; Cameriere, V.; et al. Diagnostic performance of new and classic CSF biomarkers in age-related dementias. *Aging* **2019**, *11*, 2420–2429.
309. Jia, L.H.; Liu, Y.N. Downregulated serum miR-223 servers as biomarker in Alzheimer's disease. *Cell Biochem. Funct.* **2016**, *34*, 233–237.
310. Wei, H.; Xu, Y.; Xu, W.; Zhou, Q.; Chen, Q.; Yang, M.; Feng, F.; Liu, Y.; Zhu, X.; Yu, M.; et al. Serum exosomal miR-223 serves as a potential diagnostic and prognostic biomarker for dementia. *Neuroscience* **2018**, *379*, 167–176.
311. Ren, R.J.; Zhang, Y.F.; Dammer, E.B.; Zhou, Y.; Wang, L.L.; Liu, X.H.; Feng, B.L.; Jiang, G.X.; Chen, S.D.; Wang, G.; et al. Peripheral blood microRNA expression profiles in Alzheimer's disease: Screening, validation, association with clinical phenotype and implications for molecular mechanism. *Mol. Neurobiol.* **2016**, *53*, 5772–5781.
312. Kumar, S.; Vijayan, M.; Reddy, P.H. MicroRNA-455-3p as a potential peripheral biomarker for Alzheimer's disease. *Hum. Mol. Genet.* **2017**, *26*, 3808–3822.
313. Chen, Y.; Wang, X. miRDB: An online database for prediction of functional microRNA targets. *Nucleic Acids Re*s **2020**, *48*, D127–D131.
314. Liu, W.; Wang, X. Prediction of functional microRNA targets by integrative modeling of microRNA binding and target expression data. *Genome Biol.* **2019**, *20*, 18.
